# Complexome profiling of the Chlamydomonas *psb28* mutant reveals TEF5 as an early PSII assembly factor

**DOI:** 10.1093/plcell/koaf055

**Published:** 2025-03-17

**Authors:** Julia Lang, Katharina König, Benedikt Venn, Saskia Zeilfelder, Matthias Ostermeier, Benjamin Spaniol, Lara Spaniol, Frederik Sommer, Matthieu Mustas, Stefan Geimer, Torben Fürtges, Pawel Brzezowski, Jure Zabret, Francis-André Wollman, Marc M Nowacyzk, David Scheuring, Till Rudack, Timo Mühlhaus, Yves Choquet, Michael Schroda

**Affiliations:** Molecular Biotechnology & Systems Biology, RPTU Kaiserslautern-Landau, D-67663 Kaiserslautern, Germany; Molecular Biotechnology & Systems Biology, RPTU Kaiserslautern-Landau, D-67663 Kaiserslautern, Germany; Computational Systems Biology, RPTU Kaiserslautern-Landau, D-67663 Kaiserslautern, Germany; Molecular Biotechnology & Systems Biology, RPTU Kaiserslautern-Landau, D-67663 Kaiserslautern, Germany; Molecular Plant Science, LMU Munich, 82152 Planegg-Martinsried, Germany; Molecular Biotechnology & Systems Biology, RPTU Kaiserslautern-Landau, D-67663 Kaiserslautern, Germany; Molecular Biotechnology & Systems Biology, RPTU Kaiserslautern-Landau, D-67663 Kaiserslautern, Germany; Molecular Biotechnology & Systems Biology, RPTU Kaiserslautern-Landau, D-67663 Kaiserslautern, Germany; Biologie du Chloroplaste et Perception de la Lumière chez les Microalgues, Institut de Biologie Physico-Chimique, UMR CNRS/UPMC 7141, F-75005 Paris, France; Zellbiologie/Elektronenmikroskopie, Universität Bayreuth, D-95440 Bayreuth, Germany; Structural Bioinformatics, Regensburg Center for Biochemistry, Regensburg Center for Ultrafast Nanoscopy, University of Regensburg, D-93053 Regensburg, Germany; Faculty of Biochemistry, Biophysics and Biotechnology, Department of Plant Physiology and Biochemistry, Jagiellonian University in Cracow, 30-387 Cracow, Poland; Department of Plant Biochemistry, Faculty of Biology and Biotechnology, Ruhr University Bochum, D-44801 Bochum, Germany; Biologie du Chloroplaste et Perception de la Lumière chez les Microalgues, Institut de Biologie Physico-Chimique, UMR CNRS/UPMC 7141, F-75005 Paris, France; Department of Biochemistry, Institute of Biosciences, University of Rostock, D-18059 Rostock, Germany; Plant Pathology, RPTU Kaiserslautern-Landau, D-67663 Kaiserslautern, Germany; Structural Bioinformatics, Regensburg Center for Biochemistry, Regensburg Center for Ultrafast Nanoscopy, University of Regensburg, D-93053 Regensburg, Germany; Computational Systems Biology, RPTU Kaiserslautern-Landau, D-67663 Kaiserslautern, Germany; Biologie du Chloroplaste et Perception de la Lumière chez les Microalgues, Institut de Biologie Physico-Chimique, UMR CNRS/UPMC 7141, F-75005 Paris, France; Molecular Biotechnology & Systems Biology, RPTU Kaiserslautern-Landau, D-67663 Kaiserslautern, Germany

## Abstract

PSII assembly requires auxiliary factors, including Psb28. Although the absence of Psb28 in cyanobacteria has little effect on PSII assembly, we show here that the Chlamydomonas (*Chlamydomonas reinhardtii*) *psb28* null mutant is severely impaired in PSII assembly, showing drastically reduced PSII supercomplexes, dimers, and monomers, while overaccumulating early PSII assembly intermediates reaction center II (RCII), CP43_mod_, and D1_mod_. The mutant had less PSI and more cytochrome *b_6_f* complex, its thylakoids were organized mainly as monolayers, and it had a distorted chloroplast morphology. Complexome profiling of the *psb28* mutant revealed that THYLAKOID ENRICHED FRACTION 5 (TEF5), the homolog of Arabidopsis (*Arabidopsis thaliana*) PHOTOSYSTEM B PROTEIN 33/LIGHT HARVESTING-LIKE 8, comigrated particularly with RCII. TEF5 also interacted with PSI. A Chlamydomonas *tef5* null mutant was severely impaired in PSII assembly and overaccumulated RCII and CP43_mod_. RC47 was not detectable in the light-grown *tef5* mutant. Our data suggest a possible role for TEF5 in RCII photoprotection or maturation. Both the *psb28* and *tef5* mutants exhibited decreased synthesis of CP47 and PsbH, suggesting negative feedback regulation possibly exerted by the accumulation of RCII and/or CP43_mod_ in both mutants. The strong effects of missing auxiliary factors on PSII assembly in Chlamydomonas suggest a more effective protein quality control system in this alga than in land plants and cyanobacteria.

## Teaser

Complexome profiling of the Chlamydomonas *psb28* mutant identified THYLAKOID ENRICHED FRACTION 5 as a factor involved in early steps of PSII assembly.

## Introduction

PSII is a light-driven water-plastoquinone oxidoreductase in the thylakoid membranes of cyanobacteria and chloroplasts. Structural analyses of the PSII core complex from spinach and pea revealed 4 large intrinsic subunits, D1 (PsbA), D2 (PsbD), CP43 (PsbC), and CP47 (PsbB), as well as 12 small membrane-spanning subunits, PsbE, PsbF, PsbH, PsbI, PsbJ, PsbK, PsbL, PsbM, PsbTc, PsbW, PsbX, and PsbZ. Moreover, there were 4 extrinsic subunits on the luminal side, including oxygen-evolving complex proteins (PsbO, PsbP, and PsbQ) and PsbTn (Wei et al. 2016; Su et al. 2017). Structural analyses of PSII from *Chlamydomonas reinhardtii* (Chlamydomonas) revealed the same subunits as found in the PSII core from land plants, but 2 more peripheral subunits were detected, Psb30 and PsbR, while PsbTn was absent. Moreover, 2 new densities referred to as unidentified stromal protein and small luminal protein were detected (Sheng et al. 2019, 2021). PSII core monomers assemble into dimers, to which peripheral antennae bind on both sides to form PSII supercomplexes. In land plants, a PSII dimer binds 2 copies each of the monomeric minor antenna CP24 (LHCB6), CP26 (LHCB5), and CP29 (LHCB4) as well as up to 4 major LIGHT HARVESTING COMPLEX II (LHCII) heterotrimers (Caffarri et al. 2009; Kouril et al. 2011; Su et al. 2017). In Chlamydomonas, which lacks CP24, a PSII dimer binds 2 each of the CP26 and CP29 monomers as well as up to 6 large LHCII heterotrimers (Tokutsu et al. 2012; Sheng et al. 2019, 2021).

Based largely on the seminal work on cyanobacterial PSII, the steps leading to the formation of PSII core complexes from 4 preassembled modules termed D1_mod_, D2_mod_, CP47_mod_, and CP43_mod_ have been revealed (Komenda et al. 2024): PSII assembly starts with the synthesis of the α- and β-subunits (PsbE and PsbF) of cytochrome (Cyt) *b_559_*, which accumulates in the membrane and interacts with newly made D2 to form the D2_mod_ (Morais et al. 1998; Muller and Eichacker 1999; Komenda et al. 2004). In parallel, the newly synthesized D1 precursor interacts with PsbI that has already been produced. PsbI-D1 (D1_mod_) is then combined with the D2_mod_ to form the reaction center (RCII) (Dobakova et al. 2007; Zhao et al. 2023). This is followed by proteolytic processing of the D1 precursor at its C-terminus (Anbudurai et al. 1994). With the low molecular mass subunits PsbH, PsbL, PsbM, PsbR, and PsbTc, CP47 forms the CP47_mod_ that combines with RCII to form the RC47 intermediate, which also contains PsbX and PsbY (Rokka et al. 2005; Boehm et al. 2012). CP43 interacts with the small subunits PsbK, PsbZ, and Psb30 and forms the CP43_mod_, which finally combines with RC47 to form PSII monomers (Sugimoto and Takahashi 2003; Rokka et al. 2005; Boehm et al. 2011). During photoactivation in chloroplasts, the Mn_4_CaO_5_ cluster is attached to the luminal side of the PSII monomers, followed by the proteins PsbO, PsbP, and PsbQ (Bricker et al. 2012). After dimerization and attachment of LHCII, the assembly is complete and the supercomplex is transferred from stroma-exposed membranes to grana stacks (Tokutsu et al. 2012; van Bezouwen et al. 2017).

The assembly of PSII is facilitated by auxiliary factors that temporarily bind to discrete assembly intermediates, but they are not constituents of the final complex. Many, but not all of these auxiliary factors are conserved between cyanobacteria and chloroplasts (Nixon et al. 2010; Nickelsen and Rengstl 2013; Lu 2016; Komenda et al. 2024). For example, auxiliary factors HIGH CHLOROPHYLL FLUORESCENCE 136 (HCF136) (hypothetical chloroplast open reading frame 48 [Ycf48] in *Synechocystis*), PsbN, PHOTOSYNTHESIS AFFECTED MUTANT 68 (PAM68), PSB28, HCF244 (Ycf39 in *Synechocystis*), and rubredoxin 1 (RBD1) (RubA in *Synechocystis*) are conserved between chloroplasts and cyanobacteria, while factors such as Psb34 and Psb35 exist only in cyanobacteria and factors such as LOW PSII ACCUMULATION 2 (LPA2) exist only in chloroplasts. In *Synechocystis*, the conserved assembly factor Psb28-1 is peripherally associated at the cytoplasmic side mainly with RC47 and less with PSII monomers (Kashino et al. 2002; Dobakova et al. 2009; Sakata et al. 2013). Since Psb28 interacts with RC47 and PSII monomers only transiently, PSII complexes with Psb28 can be enriched in cyanobacterial mutants that accumulate PSII assembly intermediates, such as deletion mutants of *psbC* (Boehm et al. 2012), *psbJ* (Nowaczyk et al. 2012; Zabret et al. 2021), or *psbV* (Xiao et al. 2021). PSII assembles completely in the *Synechocystis psb28-1* mutant and is photochemically fully active (Dobakova et al. 2009; Sakata et al. 2013; Beckova et al. 2017). Accordingly, *psb28-1* showed no growth phenotype at various light intensities at 30 °C, but a growth defect was observed at 38 °C and light intensities of 30 *µ*mol photons m^−2^ s^−1^ or higher (Sakata et al. 2013). Moreover, the *psb28-1* mutant was more sensitive to fluctuating light (Beckova et al. 2017). The isolation of tagged Psb28 from the cyanobacterial *psbJ* and *psbV* mutants allowed determining the cryo-EM structures of Psb28 bound to RC47 and PSII monomers (Xiao et al. 2021; Zabret et al. 2021). The Psb28-RC47 complex contained PSII subunits D1, D2, CP47, PsbE, PsbF, PsbH, PsbI, PsbL, PsbM, PsbT, and PsbX, while the Psb28-PSII monomer complex also contained the CP43_mod_ bound in a premature conformation. Psb28 was found to associate with D1, D2, and CP47 directly at the cytosolic surface of PSII. Psb28 binding induces the formation of an extended β-hairpin structure that incorporates Psb28's central antiparallel β-sheet, the C-terminus of CP47, and the D–E loop of D1. Psb28 binding causes large structural changes at the D–E loop regions of D1 and D2 when compared with native PSII, which affects the environment of the Q_A/B_ binding sites and the nonhaem iron, potentially changing the Q_A_/$Q_{A}^{-}$ redox potential to reduce singlet oxygen production and thus prevent photodamage (Xiao et al. 2021; Zabret et al. 2021).

The function of some PSII auxiliary factors is less clear. An example is PHOTOSYSTEM B PROTEIN 33 (PSB33)/LIGHT HARVESTING-LIKE 8 (LIL8), which interacts with RC47 and larger PSII assembly states, but mainly with PSII monomers, and locates to stroma lamellae and grana margins in *Arabidopsis thaliana* (Arabidopsis) (Fristedt et al. 2015, 2017; Kato et al. 2017; Nilsson et al. 2020). Arabidopsis *psb33/lil8* mutants give rise to an “emergent” PSII phenotype that was only observed during a suite of varying light treatments over 5 d (Cruz et al. 2016), possibly explaining the very different mutant phenotypes reported. The “emergent” PSII phenotype was attributed to the formation of a fraction of PSII centers defective in $Q_{A}^{-}$ reoxidation, possibly related to damage to the PSII Q_B_ site, which comes along with a more oxidized PQ pool reported by Kato et al. (2017).

Complexome profiling (CP) is based on the analysis of membrane protein complexes in gel bands of blue-native (BN) gels by MS and can reveal assembly factors based on their comigration with assembly intermediates (Heide et al. 2012; Heide and Wittig 2013). We have previously employed CP on the Chlamydomonas *lpa2* mutant, which allowed us to identify factors involved in PSII assembly steps beyond RCII (Spaniol et al. 2022). We found PSB28 to comigrate with RC47 and PSII monomers in the *lpa2* mutant but not in wild type (WT), suggesting a conserved role of PSB28 in PSII assembly. Since PSB28 was not studied yet in molecular detail in chloroplasts, we characterized the Chlamydomonas *psb28* mutant in this study. Unexpectedly, we found that the *psb28* mutant is strongly impaired in accumulating PSII assemblies beyond RCII, very much in contrast to cyanobacterial *psb28* mutants. We used CP on *psb28* and we found THYLAKOID ENRICHED FRACTION 5 (TEF5), the homolog of Arabidopsis PSB33/LIL8, to comigrate with early PSII assembly intermediates, particularly RCII. We characterized the Chlamydomonas *tef5* mutant and we found that it is strongly affected in the accumulation of PSII, with hardly detectable RC47, which is mitigated in the dark. This suggests a role of TEF5 in photoprotection or maturation of RCII in early PSII assembly steps.

## Results

### The Chlamydomonas *psb28* mutant accumulates less PSII and PSI subunits and shows impaired growth in high light and under photoautotrophic conditions

**Figure 1.**
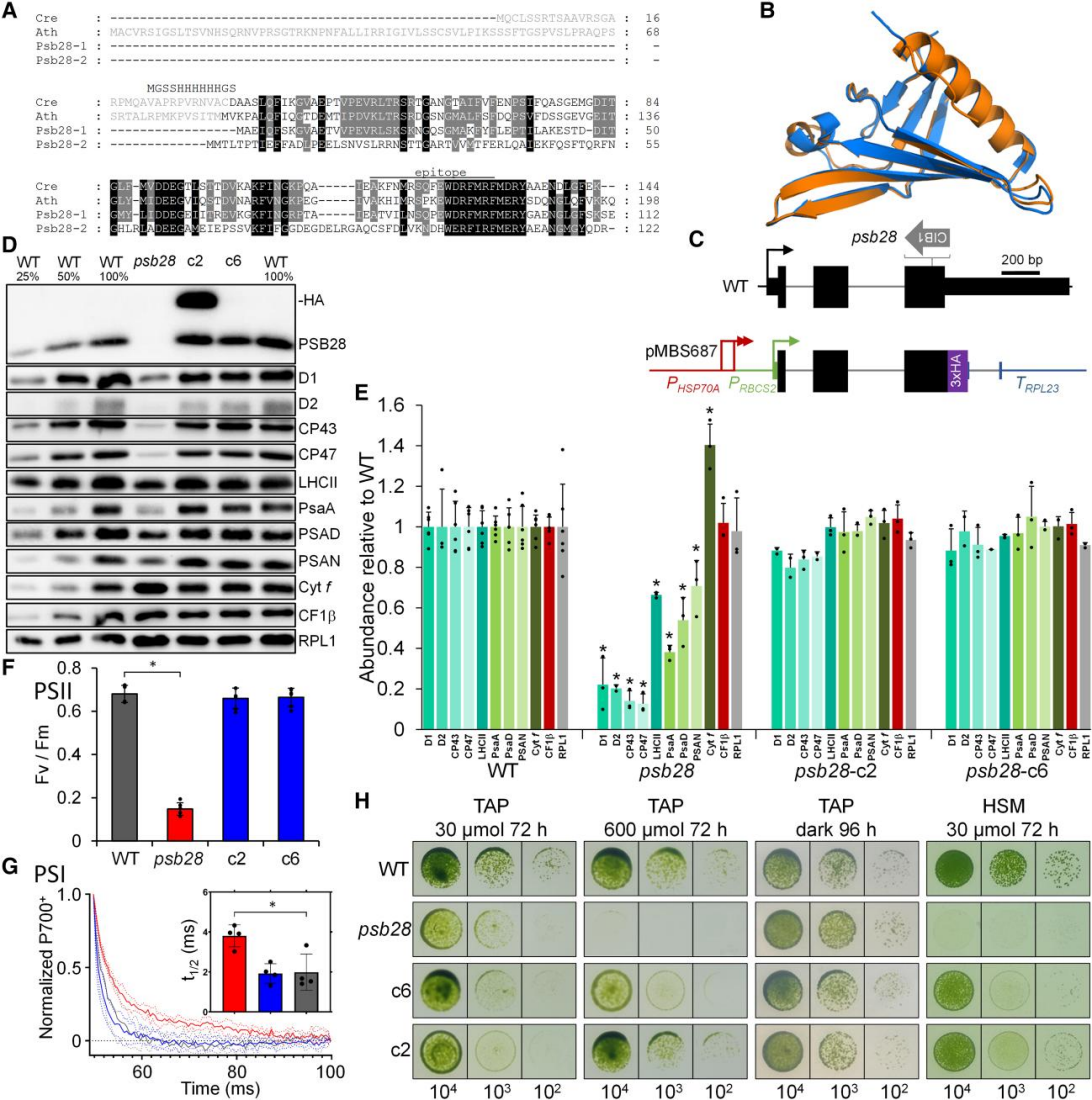
Phenotypes of the *psb28* mutant compared to WT and complemented lines. **A)** Alignment of PSB28 amino acid sequences from Chlamydomonas, Arabidopsis, and *Synechocystis*. Residues highlighted in black and gray are conserved in 4 and 3 of the sequences, respectively. Predicted chloroplast transit peptides are shown in gray font. The sequence with a hexahistidine tag replacing the transit peptide for production of recombinant Chlamydomonas PSB28 is shown. The peptide from Chlamydomonas PSB28 used for antibody production is indicated by a horizontal line. Ath, *A.thaliana* (AT4G28660); Cre, *C. reinhardtii* (Cre10.g440450); Psb28-1, *Synechocystis* sp. PCC 6803 variant 1 (Sll1398); Psb28-2, *Synechocystis* sp. variant 2 (Slr1739). **B)** Pairwise structure alignment of the crystal structure of Psb28 from *T. elongatus* (3ZPN, orange) with the AlphaFold structure of Chlamydomonas PSB28 lacking the chloroplast transit peptide (blue). **C)** Structure of the Chlamydomonas *PSB28* gene, insertion site of the CIB1 cassette (thick gray arrow) in the *psb28* mutant, and construct for complementation. Protein coding regions are drawn as black and purple boxes, UTRs as bars, and introns and promoter regions as thin lines. *HSP70A* and *RBCS2* promoters and 5′ UTRs are drawn in red and green, respectively. *RPL23* terminator sequences are drawn in blue. Thin arrows indicate transcriptional start sites. **D)** Immunoblot analysis of the accumulation of PSB28 and of subunits of the major thylakoid membrane protein complexes using antisera against PSB28 and the indicated subunits, respectively. c2 and c6 are lines complemented with the construct shown in **C)**. HA indicates the position of PSB28-3xHA. PSII, D1, D2, CP43, CP47, and LHCII; PSI, PsaA, PSAD and PSAN; Cyt *b_6_f* complex, Cyt *f*; and ATP synthase, CF1β. Ribosomal protein RPL1 served as loading control. Ten micrograms of whole-cell proteins (100%) were analyzed. **E)** Quantification of the immunoblot analysis shown in **D)**. Values are means from 3 independent experiments (6 for the WT) normalized first by the median of all signals obtained with a particular antiserum in the same experiment and then by the mean signal of the WT. Error bars represent Sd. Asterisks indicate significant differences with respect to the WT (2-tailed, unpaired *t*-test with Bonferroni–Holm correction, *P* < 0.05). The absence of an asterisk means that there were no significant differences. **F)**  *F*_v_/*F*_m_ values of the *psb28* mutant versus WT and complemented lines. Shown are averages from 6 independent experiments. Error bars represent Sd. The asterisk indicates significant differences between WT and *psb28* mutant/complemented lines (2-tailed, unpaired *t*-test with Bonferroni–Holm correction, *P* < 0.001). **G)** PSI reduction kinetics of WT (gray), *psb28* mutant (red) and a complemented line (blue). Shown are averages from 4 independent experiments fitted with single exponential functions. Sd is shown as dotted lines. The asterisk indicates significant differences between WT and *psb28* mutant (1-way ANOVA, *P* < 0.01). **H)** Analysis of the growth of 10^4^ to 10^2^ spotted cells under the conditions indicated.

*Synechocystis* sp. (strain *PCC 6803*) is equipped with 2 functionally distinct Psb28 homologs, Psb28-1 and Psb28-2 (Dobakova et al. 2009; Sakata et al. 2013; Beckova et al. 2017), while only single PSB28 proteins exist in Arabidopsis and Chlamydomonas. As shown in Fig. 1A, Chlamydomonas and Arabidopsis PSB28 proteins are more closely related to *Synechocystis* Psb28-1 (58/71% and 45/69% identical/similar amino acid residues, respectively) than to Psb28-2 (28/45% and 34/54% identical/similar amino acid residues, respectively) and harbor predicted chloroplast transit peptides. The structure of Chlamydomonas PSB28 predicted by AlphaFold is very similar to the crystal structure of *Thermosynechococcus elongatus* (Bialek et al. 2013) (RMSD = 1.27 Å; template modeling [TM] score = 0.93) (Fig. 1B).

The Chlamydomonas *psb28* mutant (Li et al. 2016) contains the CIB1 mutagenesis cassette within the third exon of the *PSB28* gene (Fig. 1C). While we were able to amplify *PSB28* sequences flanking the cassette on the 5′ side by PCR, no PCR product was obtained on the 3′ side, presumably because flanking sequences were deleted, or a large piece of junk DNA had integrated between *PSB28* sequences and the CIB1 cassette (Supplementary Fig. S1, A and B). An antibody raised against a peptide from the C-terminal part of the Chlamydomonas PSB28 protein (Fig. 1A) specifically detected a protein band at the expected molecular mass of ∼12.5 kDa in the WT, which was absent in the *psb28* Chlamydomonas Library Project (CLiP) mutant (Fig. 1D; Supplementary Fig. S1C). In the *psb28* mutant, PSII core subunits accumulated at most to 22% and LHCIIs to ∼66% of WT levels (Fig. 1, D and E). PSI core subunits accumulated to between 38% and 71% of WT levels. While the abundance of ATP synthase subunit CF1β was unaltered between mutant and WT, Cyt *f* of the Cyt *b_6_f* complex was 1.4-fold more abundant in the mutant compared with the WT. We amplified the genomic *PSB28* coding sequence, fused it with a sequence encoding a C-terminal 3xhemagglutinin (HA) tag and placed it under control of the constitutive *HEAT SHOCK PROTEIN 70A–RIBULOSE-1,5-BISPHOSPHATE CARBOXYLASE/OXYGENASE SMALL SUBUNIT 2* (*HSP70A-RBCS2*) promoter and the *RIBOSOMAL PROTEIN L23* (*RPL23*) terminator using modular cloning (Fig. 1C) (Schroda et al. 2000; Crozet et al. 2018). We combined the *PSB28* transcription unit with the *aadA* cassette (Meslet-Cladiere and Vallon 2011) and transformed it into the *psb28* mutant. Seven picked spectinomycin-resistant transformants that showed a greener appearance than the *psb28* mutant accumulated D1 roughly at WT levels and, in all but 2, the HA tag was detected (Supplementary Fig. S2; Fig. 1D). Further analysis with the PSB28 peptide antibody of 2 transformants with (*psb28*-c2) and without (*psb28*-c6) detectable HA signal revealed that in both lines, PSB28 accumulated to the WT level with the molecular mass corresponding to the WT protein (Fig. 1D). In line *psb28*-c2, PSB28 with 3xHA tag accumulated additionally. These findings point to a processing of the 3xHA tag and to a controlled accumulation of the processed WT protein. The reduced accumulation of photosystem core subunits and LHCII and the increased accumulation of Cyt *f* were fully reversed in both complemented lines (Fig. 1, D and E). In accordance with the reduced accumulation of PSII core subunits, PSII maximum quantum yield, as indicated by the *F*_v_/*F*_m_ value, was strongly reduced in the *psb28* mutant (0.15) versus WT and complemented lines (0.66 to 0.68) (Fig. 1F). The half-life of P700^+^ reduction was about twice as high in the *psb28* mutant compared to WT and a complemented line, indicating reduced electron flow through PSI in the mutant (Fig. 1G). While the *psb28* mutant could grow under heterotrophic conditions and under mixotrophic conditions in low light (30 *µ*mol photons m^−2^ s^−1^), it failed to grow under mixotrophic conditions in high light (600 *µ*mol photons m^−2^ s^−1^) and under photoautotrophic conditions in low light (Fig. 1H).

A *Synechocystis psb28-1* mutant was reported to accumulate magnesium protoporphyrin IX monomethyl ester and to contain a decreased level of protochlorophyllide, indicating inhibition of chlorophyll (Chl) biosynthesis at the cyclization step and suggesting a role of Psb28-1 in regulating Chl biosynthesis (Dobakova et al. 2009). Later work indicated that this phenotype was due to a defect in the strain background used for mutant construction (Beckova et al. 2017). To investigate whether PSB28 might regulate a specific step in Chl biosynthesis in Chlamydomonas, we measured the content of Chl a and b, of several Chl precursors, and of Chl breakdown product pheophorbide by HPLC in the WT and the *psb28* mutant grown in low light or in the dark for 65 h. All analyzed pigments accumulated to lower levels in the mutant when compared with the WT under both growth conditions, except for Chl a and Chl b, which accumulated to similarly low levels in the WT and the mutant grown in the dark (Supplementary Fig. S3). This was observed also in spot tests of dark-grown cells (Fig. 1H). Overall, the reduced levels of Chl and of all its precursors in the mutant versus the WT point to an overall reduced Chl synthesis in the mutant rather than to a specific block at a particular synthesis step.

### PSB28 is localized to the chloroplast, where its absence results in severe defects of chloroplast morphology and thylakoid ultrastructure

**Figure 2.**
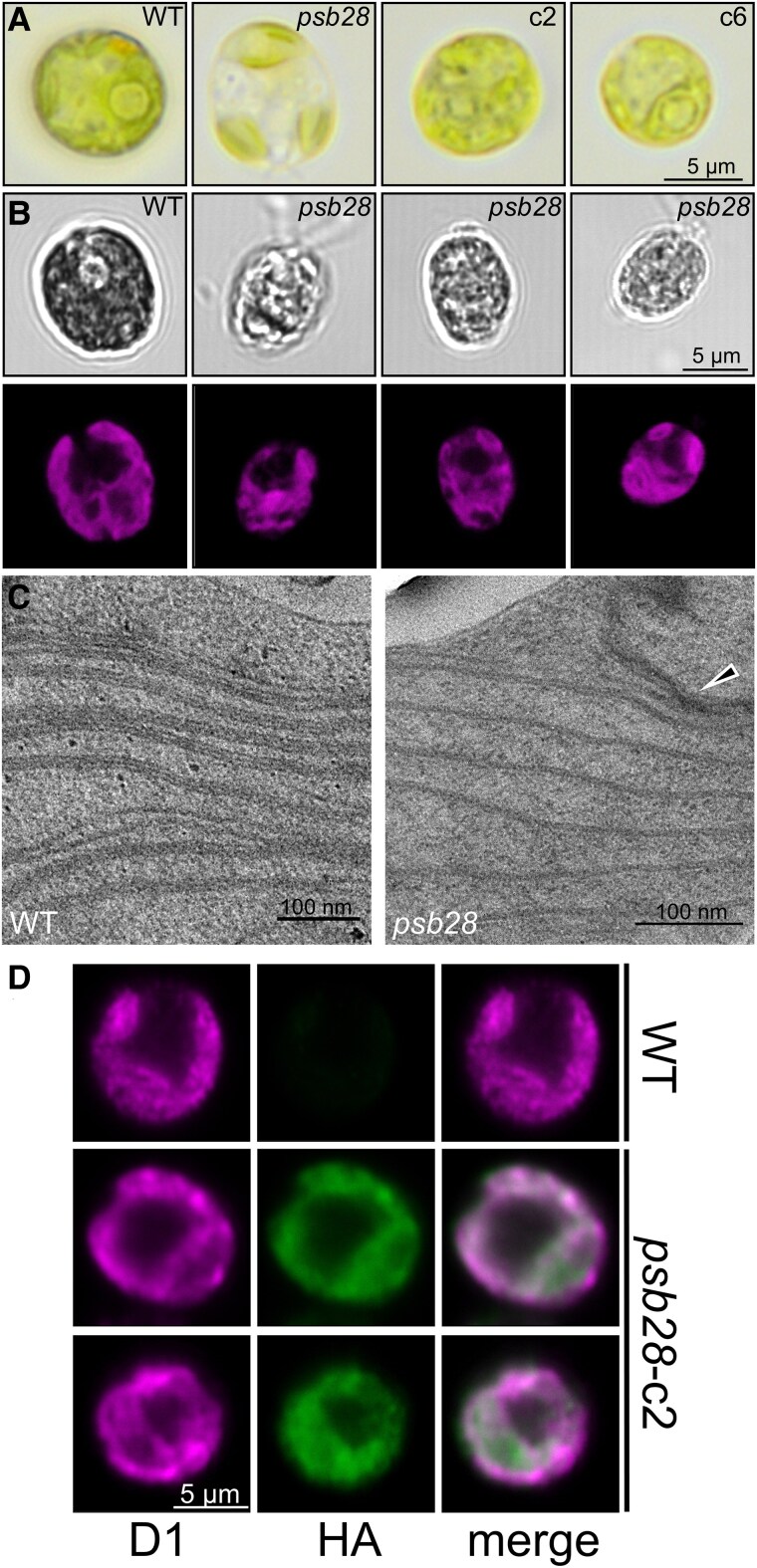
Light, fluorescence, and electron microscopy of the *psb28* mutant and localization of PSB28 by immunofluorescence. **A)** Light microscopy images of WT, *psb28* mutant, and complemented lines grown under mixotrophic conditions in low light (30 *µ*mol photons m^−2^ s^−1^). More images are shown in Supplementary Fig. S4A. **B)** Confocal laser-scanning fluorescence microscopy of WT and 3 *psb28* cells. Shown are bright-field images (top) and Chl autofluorescence (bottom). More images are shown in Supplementary Fig. S4B. **C)** Electron microscopy images of WT and *psb28* mutant. The black triangle points to a rarely occurring thylakoid membrane stack in the mutant. More images are shown in Supplementary Fig. S5. **D)** Immunofluorescence localization of the D1 protein (magenta) and HA-tagged PSB28 (green) in a WT cell and 2 complemented *psb28* mutant cells (*psb28*-c2). The shown localization pattern was observed in 5 different *psb28*-c2 cells. A single scale bar applies to all images displayed in that panel.

To investigate whether the absence of PSB28 affected cell morphology and thylakoid ultrastructure, we used light, fluorescence, and transmission electron microscopy (TEM), respectively. Light microscopy and fluorescence microscopy, recording Chl autofluorescence, revealed an abnormal chloroplast in the *psb28* mutant with reduced green or fluorescing areas (Fig. 2, A and B; Supplementary Fig. S4). This phenotype was restored in the complemented lines. TEM revealed that the thylakoid membranes in the mutant are almost exclusively present as monolayers, with stacks occurring only occasionally, while in the WT thylakoid membranes are commonly found in stacks (Engel et al. 2015) (Fig. 2C; Supplementary Fig. S5). Using a mouse antibody against the HA tag and a rabbit antibody against D1, we determined the intracellular localization of PSB28-3xHA in the complemented line *psb28*-c2 by immunofluorescence. As shown in Fig. 2D, PSB28-3xHA was detected in the cup-shaped chloroplast and colocalized with D1 in most areas, but there were also areas where PSB28-3xHA was present but not D1, particularly around the pyrenoid. Notice that only the fraction of PSB28 was detected, which still contained a C-terminal 3xHA tag.

### The synthesis of subunits of both photosystems is impaired in the *psb28* mutant

**Figure 3.**
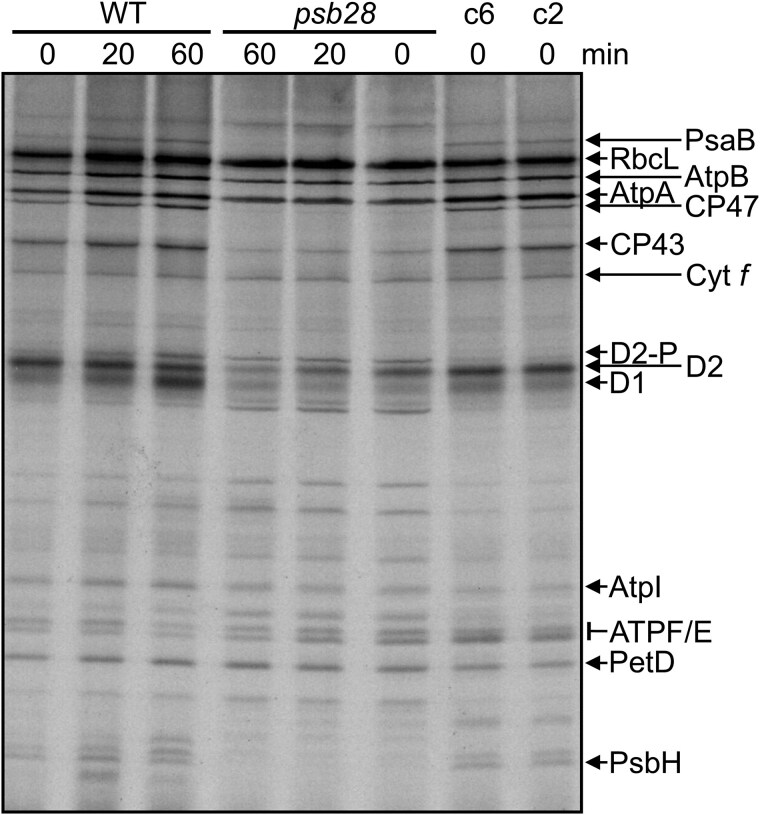
Analysis of synthesis and stability of thylakoid membrane proteins in the *psb28* mutant by pulse-chase labeling. WT, *psb28* mutant, and complemented lines c6 and c2 were labeled with ^14^C-acetate in low light (20 *µ*mol photons m^−2^ s^−1^) for 7 min in the presence of cytosolic translation inhibitor cycloheximide (0) and chased with unlabeled acetate for 20 and 60 min. Proteins were separated on a 12% to 18% SDS-urea gel and visualized by autoradiography. The assignment of the protein bands is based on mutant analyses (de Vitry et al. 1989; Girard-Bascou et al. 1992; Minai et al. 2006).

To investigate the effects of the lack of PSB28 on the synthesis and stability of newly made chloroplast-encoded photosynthetic proteins, we performed pulse-chase analyses with ^14^C-acetate on the WT, the *psb28* mutant, and complemented lines *psb28*-c2 and *psb28*-c6. Comparing *psb28* with the WT, we defined no and reduced radioactivity in a protein after the 7-min pulse as indicative for severely impaired and reduced protein synthesis, respectively, and reduced radioactivity during the 60-min chase as indicative for reduced protein stability. Based on these definitions, the synthesis of PsaB, CP47, and PsbH was severely impaired in the *psb28* mutant (Fig. 3). Synthesis and stability of D1, D2, and CP43 were reduced in the mutant, whereas synthesis and stability of RbcL and of subunits of the Cyt *b_6_f* complex and the ATP synthase were not affected when compared with the WT. These defects in the *psb28* mutant were fully restored in both complemented lines.

### PSII assembly states beyond RCII are severely reduced in the *psb28* mutant

**Figure 4.**
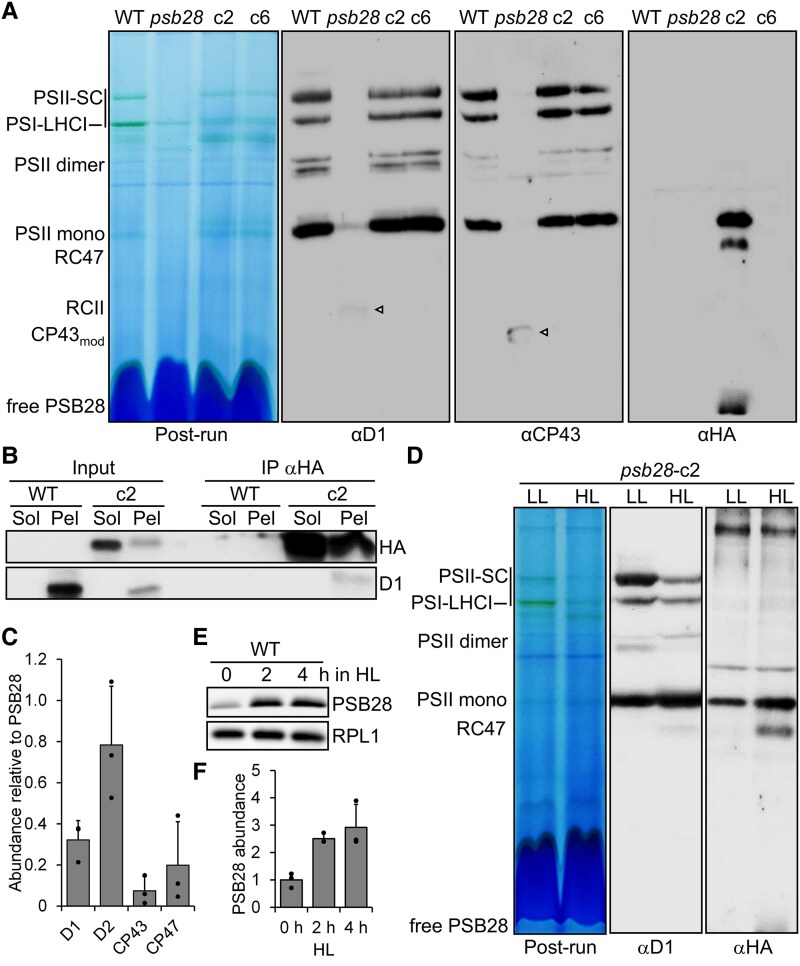
Analysis of protein complexes in the *psb28* mutant and of PSB28 interaction partners. **A)** BN–PAGE analysis of proteins from cells grown in LL (30 *µ*mol photons m^−2^ s^−1^). Fifty micrograms of whole-cell proteins from WT, *psb28* mutant, and complemented lines *psb28*-c2 and *psb28*-c6 were solubilized with 1% β-DDM and separated on a 4% to 15% BN gel. Shown is a picture of the gel after the run and an immunoblot detected with antibodies against D1, CP43, and the HA epitope. Arrowheads point to faint bands likely representing RC47 and CP43_mod_ in the *psb28* mutant. **B)** Immunoprecipitation of PSB28. Cells from complemented line *psb28*-c2 were fractionated via freeze–thaw cycles and centrifugation. HA-tagged PSB28 was then immunoprecipitated (IP) from soluble (Sol) and membrane-enriched (Pel) fractions with an HA antibody. 1% of the input and 10% of the precipitate were analyzed by SDS–PAGE and immunoblotting using antibodies against the HA epitope and D1. **C)** MS-based quantification of proteins coprecipitated from solubilized membrane fractions with HA-tagged PSB28. IBAQ values for each PSII core subunit were normalized by the IBAQ value for PSB28. Shown are mean values from 3 independent experiments. Error bars represent Sd. **D)** BN–PAGE analysis of proteins from cells grown in LL (30 *µ*mol photons m^−2^ s^−1^) and then exposed to HL (1,200 *µ*mol photons m^−2^ s^−1^) for 4 h. Whole-cell proteins from complemented line *psb28*-c2 were solubilized with 1% β-DDM and separated on a 4% to 15% BN gel. Shown is a picture of the gel after the run and an immunoblot detected with antibodies against D1 and the HA epitope. **E)** Analysis of PSB28 accumulation in HL. WT was exposed to 1,200 *µ*mol photons m^−2^ s^−1^ for 4 h, and samples taken prior, 2, and 4 h after the treatment were analyzed by immunoblotting using the peptide antibody against PSB28 and an antibody against RPL1 as loading control. **F)** Quantification of the immunoblot analysis shown in **E)**. Values are means from 3 independent experiments. Normalization was done as described for Fig. 1D. Error bars represent Sd. HL, high light; LL, low light; SC, supercomplexes.

To assess how the lack of PSB28 affects the PSII assembly states, we analyzed whole-cell protein extracts from the low light-grown WT, the *psb28* mutant, and the complemented lines by BN–PAGE and immunoblotting. While PSII supercomplexes, dimers, and monomers were detected with similar intensities in the WT and the complemented lines with antibodies against D1 and CP43, only a very faint signal for PSII monomers was detected in the *psb28* mutant with the D1 antibody (Fig. 4A). However, we could detect RCII and CP43_mod_ in the mutant, which were not detectable in the WT and the complemented lines.

We wondered whether the reduced accumulation of PSII and PSI core subunits and the reduced accumulation of PSII assembly states, beyond the RCII in the *psb28* mutant, were due to light-induced damage. To test this, we compared protein complexes in solubilized whole-cell extracts from the WT and the *psb28* mutant grown in low light and in the dark for 45 h. We observed an equally impaired accumulation of PSII and PSI complexes in the *psb28* mutant under both growth conditions (Supplementary Fig. S6A). As determined by SDS–PAGE and immunoblotting, the accumulation of D1, CP43, and PsaA in the mutant was similarly affected in low light and in the dark (Supplementary Fig. S6B). Nevertheless, the *F*_v_/*F*_m_ value in the *psb28* mutant was slightly higher in dark-grown versus light-grown cells (0.2 vs 0.12, *P* = 0.014), while the opposite was observed for the WT (0.66 vs. 0.53, *P* = 0.001) (Supplementary Fig. S6C). We conclude that the reduced accumulation of photosystems in the *psb28* mutant is not caused by damage inflicted by light.

### PSB28 interacts with complexes containing D2, D1, CP47, and CP43

Detection with the HA antibody revealed that PSB28-3xHA comigrated with PSII monomers and, to a lesser extent, with RC47 in the *psb28*-c2 line (Fig. 4A) (we did not use the antibody against PSB28 because it showed many cross-reactions with other proteins; Supplementary Fig. S1C). This suggests that excess PSB28-3xHA contributes to the pool of functional PSB28 in this line (nonfunctional binding of PSB28-3xHA to RC47 would likely block further assembly of PSII and cause a dominant-negative phenotype, which we did not see). A substantial fraction of free PSB28-3xHA was detected, as well. To rule out that PSB28 forms oligomers that comigrate with PSII monomers and RC47 by chance, we analyzed migration properties of recombinantly produced PSB28 on BN gels. Recombinant PSB28 migrated entirely below the ∼25-kDa monomeric nucleotide exchange factor CHLOPLAST GRPE HOMOLOG 1 (CGE1) (Willmund et al. 2007), indicating that Chlamydomonas PSB28 forms at most dimers but no higher oligomers (Supplementary Fig. S7A). We also used recombinant PSB28 to estimate its abundance in WT cells by quantitative immunoblotting and found that PSB28 constitutes 0.0034 ± 0.001% of the total protein content in the WT cell (Supplementary Fig. S7B). Assuming ∼25 pg of total protein/cell, PSB28 would make up to 0.07 attomol/cell. Compared with an estimated 5.2 attomol PSII/cell, PSB28 would be ∼78-fold less abundant than PSII in the WT (Hammel et al. 2018, 2020).

To verify the interaction of PSB28 with RC47/PSII monomers that was implied from their comigration in BN–PAGE, we used the HA antibody to immunoprecipitate PSB28-3xHA from soluble and membrane-enriched fractions prepared from the complemented *psb28*-c2 line. Prior to immunoprecipitation, complexes were stabilized by in vivo cross-linking with 0.37% formaldehyde. Immunoblot analyses showed that more HA-tagged PSB28 was immunoprecipitated from the soluble fraction than from the membrane fraction (Fig. 4B). Moreover, D1 was detected only in PSB28 precipitates generated from membrane fractions. To identify and quantify all the proteins interacting with PSB28, we analyzed the PSB28 immunopreciptates by LC-MS/MS (Supplementary Data Set S1). In precipitates from soluble fractions, PSB28 was the only protein detected in all 3 replicates. In precipitates from the membrane fractions, only D1, D2, CP43, and CP47 were detected in all 3 replicates, in addition to PSB28. Intensity-based absolute quantification (IBAQ) normalized to PSB28 revealed that D2 was the most abundant protein in the precipitate, followed by D1, CP47, and CP43 (Fig. 4C).

### PSB28 abundance and its association with PSII increase in high light

*Synechocystis* PSB28-1 and 2 were found in PSII-PSI supercomplexes particularly under high light intensities (Beckova et al. 2017). To test whether this is true also for Chlamydomonas PSB28, we exposed the complemented line *psb28*-c2 to low and high light intensities, solubilized whole-cell proteins, separated them by BN–PAGE, and detected D1 and HA-tagged PSB28 by immunoblotting (Fig. 4D). Based on the D1 signals, more RC47 and PSII monomers accumulated at the expense of PSII supercomplexes in high versus low light. More PSB28-3xHA was associated with RC47 and PSII monomers in high versus low light, but we did not observe an increased association of PSB28-3xHA with larger complexes. However, we found a ∼2.9-fold accumulation of PSB28 protein in WT exposed to 1,200 *µ*mol photons m^−2^ s^−1^ for 4 h, pointing to a potential role of PSB28 in PSII repair (Fig. 4, E and F).

To investigate the susceptibility of PSII in the *psb28* mutant to high light and its capability to recover functional PSII, we exposed WT, *psb28* mutant, and the complemented lines to high light (1,800 *µ*mol photons m^−2^ s^−1^) in the presence of translation inhibitor chloramphenicol (CAP) for 1 h and allowed the cells to recover in the presence and absence of CAP at low light (30 *µ*mol photons m^−2^ s^−1^). All 4 lines recovered full initial PSII activity (and D1 protein levels) at similar rates within 5 h in a protein synthesis-dependent manner (Supplementary Fig. S8). We also monitored kinetics of PSII degradation and resynthesis in sulfur-depleted and sulfur-repleted cultures, respectively (Malnoe et al. 2014). Here, the *psb28* mutant lost PSII activity upon sulfur depletion faster than the WT and the complemented lines but recovered initial PSII activity (and D1 levels) with similar rates as the other lines (Supplementary Fig. S9). In summary, the very low levels of PSII in *psb28* are susceptible to photoinhibition and degradation upon sulfur deprivation but can be fully recovered at WT rates.

### Psb28-1 from *Synechocystis* partially complements the Chlamydomonas *psb28* mutant

**Figure 5.**
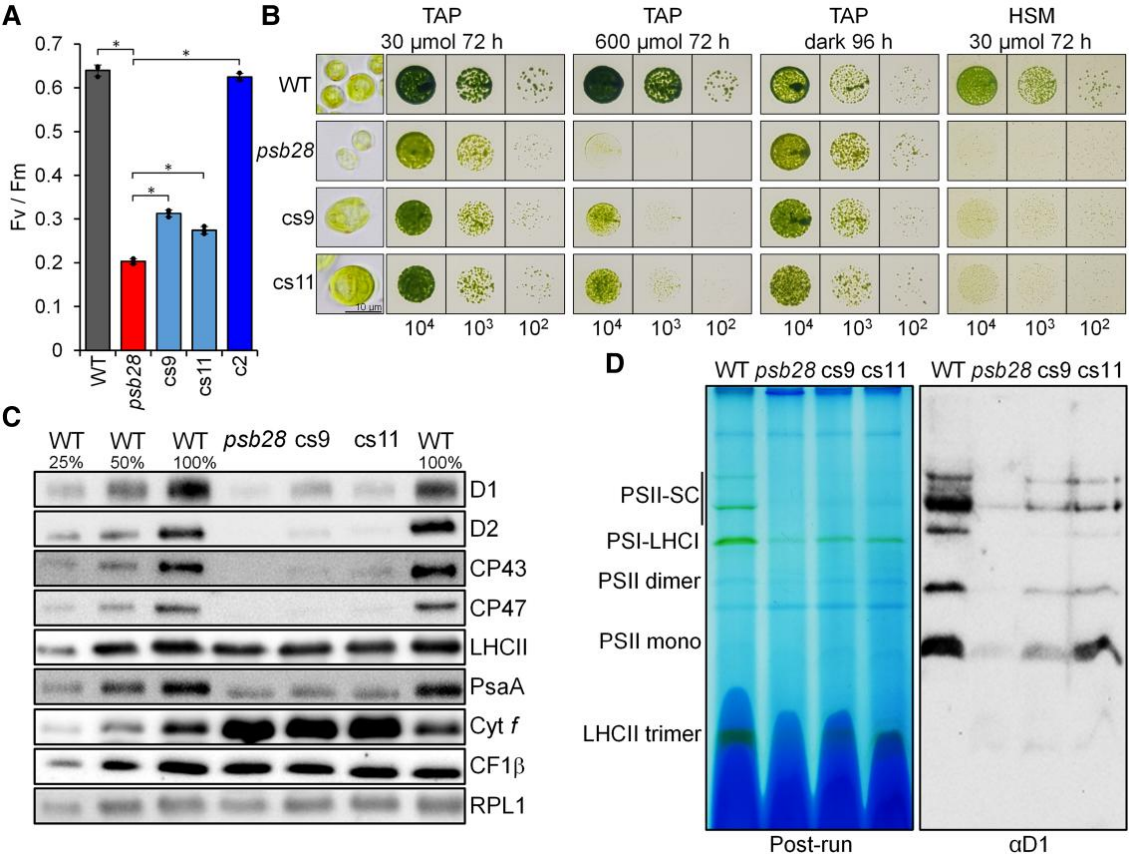
Complementation of the Chlamydomonas *psb28* mutant with *Synechocystis* Psb28-1. **A)**  *F*_v_/*F*_m_ values of the *psb28* mutant versus WT and lines complemented with Chlamydomonas PSB28 (c2) and *Synechocystis* Psb28-1 (cs9, cs11). Shown are averages from 3 independent experiments. Error bars represent Sd. Asterisks indicate significant differences with respect to the *psb28* mutant (2-tailed, unpaired *t*-test with Bonferroni–Holm correction, *P* < 0.001). **B)** Light microscopy (left) and growth analysis of 10^4^ to 10^2^ spotted cells under the conditions indicated. The scale bar shown applies to all cell images in the left panel. **C)** Immunoblot analysis of the accumulation of subunits of the major thylakoid membrane protein complexes. PSII, D1, D2, CP43, CP47, and LHCII; PSI, PsaA; Cyt *b_6_f* complex, Cyt *f*; and ATP synthase, CF1b. Ribosomal protein RPL1 served as loading control. Ten micrograms of whole-cell proteins (100%) were analyzed. **D)** BN–PAGE analysis. Cells of WT, *psb28* mutant, and complemented lines cs9 and cs11 were grown in low light (30 *µ*mol photons m^−2^ s^−1^) and solubilized with 1% β-DDM. Sixty micrograms of protein per lane were separated on a 4% to 15% BN gel. Shown is a picture of the gel after the run and an immunoblot detected with an antibody against D1.

Given the similarity between PSB28 from Chlamydomonas and Psb28-1 from *Synechocystis* (Fig. 1, A and B), we attempted to complement the Chlamydomonas *psb28* mutant with *Synechocystis* Psb28-1. For this, we synthesized the coding sequence for Psb28-1 with optimal Chlamydomonas codon usage and inserted *RBCS2* intron 1 into the coding sequence to enhance gene expression (Baier et al. 2018; Schroda 2019). We then fused the *psb28-1* gene with sequences encoding the CHLOROPLAST DNAJ HOMOLOG (CDJ1) chloroplast transit peptide (Niemeyer et al. 2021) as well as a C-terminal 3xHA tag and placed it under control of the constitutive *HSP70A-RBCS2* promoter and the *RPL23* terminator using modular cloning (Supplementary Fig. S10A). We combined the *psb28-1* transcription unit with the *aadA* cassette and transformed it into the *psb28* mutant. Twelve spectinomycin-resistant transformants (cs, complemented with *Synechocystis* Psb28-1) were analyzed for the production of the recombinant protein by immunoblotting using an HA antibody, but specific signals could not be detected. We then monitored *F*_v_/*F*_m_ values in liquid cultures and found that 7 transformants had *F*_v_/*F*_m_ values around or even below that of the *psb28* mutant but 5 had values that were significantly higher (*P* < 0.05) (Supplementary Fig. S10B). The 2 transformants with the highest *F*_v_/*F*_m_ values were cs9 and cs11 with values of 0.31 and 0.27, respectively, versus 0.2 for the *psb28* mutant (Fig. 5A). Light microscopy revealed that transformants with *F*_v_/*F*_m_ values below that of the *psb28* mutant showed the same defect in chloroplast morphology as the *psb28* mutant. However, transformants cs9 and cs11 had a WT chloroplast morphology (Fig. 5B; Supplementary Fig. S10C). Compared with the *psb28* mutant, both cs9 and cs11 showed improved growth under mixotrophic and photoautotrophic conditions in low light (30 *µ*mol photons m^−2^ s^−1^) and were less sensitive to high light intensities (600 *µ*mol photons m^−2^ s^−1^) but still fell far short of WT performance (Fig. 5B). Immunoblot analyses revealed slightly higher levels of D1, D2, CP43, CP47, and PsaA in cs9 and cs11 than in the *psb28* mutant while levels of Cyt *f* remained high (Fig. 5C). Accordingly, as revealed by BN–PAGE and immunoblotting, PSII monomers, dimers, and supercomplexes as well as PSI-LHCI were clearly more abundant in cs9 and cs11 than in the *psb28* mutant (Fig. 5D).

In summary, *Synechocystis* Psb28-1 complements the Chlamydomonas *psb28* mutant, but with low efficiency. This could be due to its very low abundance, presumably caused by the instability of the protein, as we failed to detect the HA-tagged protein. Alternatively, as observed for Chlamydomonas PSB28, the HA tag could have been cleaved off and recombinant Psb28-1 accumulated in sufficient amounts but cannot fully complement the lack of the native PSB28.

### CP of the *psb28* mutant reveals severe defects in PSII assembly beyond RCII

**Figure 6.**
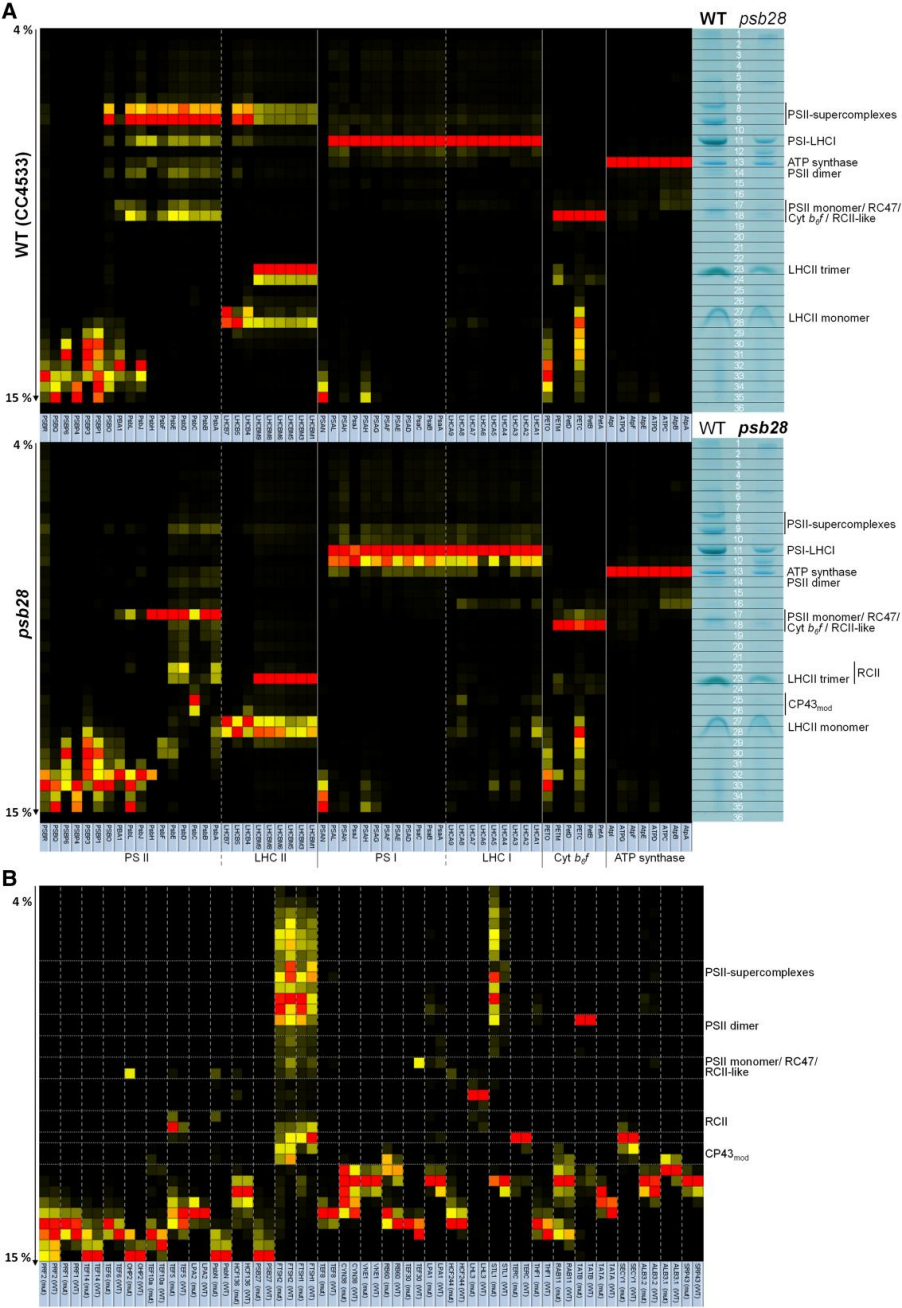
CP on WT and *psb28* mutant. **A)** Heat map showing the BN–PAGE migration profiles of subunits of the major thylakoid membrane protein complexes of WT (top panel) and *psb28* mutant (bottom panel). Values for each protein are derived from averaged peptide ion intensities from 3 biological replicates and are normalized to the gel slice with the highest intensities. The BN–PAGE lane of 1 replicate from WT and *psb28* mutant is shown with the excised band corresponding to the heat map row. The underlying data and the migration profiles for each protein are accessible in Supplementary Data Set S2. **B)** Heat map showing the BN–PAGE migration profiles of known and putative auxiliary factors involved in PSII biogenesis, repair, and the regulation of PSII complex dynamics in WT and *psb28* mutant (Supplementary Table S1).

The accumulation of early PSII assembly intermediates in the *psb28* mutant prompted us to employ CP (Heide et al. 2012; Spaniol et al. 2022) to identify early PSII assembly factors by their comigration with early PSII assembly intermediates. The analyses were performed on isolated thylakoid membranes from WT and *psb28* grown in low light (∼30 *µ*mol photons m^−2^ s^−1^) in 3 biological replicates. Thylakoid membranes were solubilized with n-dodecyl α-D-maltoside (α-DDM), and protein complexes were separated on a 4% to 15% BN gel (Supplementary Fig. S11). Each gel lane was cut into 36 slices, and the resulting 216 slices were subjected to tryptic in-gel digestion and LC-MS/MS analysis. In total, 962 proteins were identified. Summed extracted ion chromatograms of all peptides measured for a protein were used for protein quantification. To account for unequal loading, a normalization step was required. Thylakoid membranes from the *psb28* mutant lack most of PSII and part of LHCII and PSI (Fig. 1, D and E). Moreover, they form almost exclusively monolayers (Fig. 2C) and might behave differently from WT thylakoids during the extraction. Hence, normalization based on total ion intensities per lane, as done previously for CP on the *lpa2* mutant (Spaniol et al. 2022), appeared inappropriate. We therefore decided to normalize on the summed ion intensities of the 8 identified ATP synthase subunits, as the abundance of the ATP synthase appeared unaffected when whole-cell proteins from *psb28* and WT were compared (Fig. 1, D and E). Ion intensity profiles for each protein and all contributing peptides from all replicates along the BN gel run can be displayed from the interactive Excel table in Supplementary Data Set S2. The table also provides *P*-values for differences in protein abundance between WT and *psb28* as well as the distributions of peptide identification scores and peptide ion intensities. The migration profiles of all identified proteins of WT and *psb28* mutant, clustered according to their migration behavior, are shown in Supplementary Data Set S3 as heat maps. The profiles for proteins belonging to the major thylakoid membrane complexes from WT and *psb28* are shown as heat maps in Fig. 6A. Missing subunits, such as PsbI, did not give rise to detectable peptides because peptides are too small, too large, too hydrophobic or contain posttranslational modifications other than methionine oxidation or N-acetylation.

Eight subunits of the ATP synthase and 6 subunits of the Cyt *b_6_f* complex were identified. The median abundance of the Cyt *b_6_f* complex was ∼1.87-fold higher in the *psb28* mutant than in the WT (Table 1). Nevertheless, there were no differences in the migration patterns of ATP synthase and Cyt *b_6_f* complex subunits in WT and *psb28* mutant (Fig. 6A). As reported previously, PHOTOSYNTHETIC ELECTRON TRANSPORT O (PETO) did not interact stably with other subunits of the Cyt *b_6_f* complex (Takahashi et al. 2016) and a substantial fraction of the Rieske iron-sulfur protein migrated as unassembled protein (Spaniol et al. 2022).

**Table 1.** Ratio of subunit abundance between *psb28* mutant and WT

ATP synthase		Cyt *b_6_f*		PSII		PSI	
Subunit	Ratio	Subunit	Ratio	Subunit	Ratio	Subunit	Ratio
AtpA	1.03	PetA	1.97	PsbA (D1)	0.15	PsaA	1.03
AtpB	1.07	PetB	1.86	PsbB (CP47)	0.08	PsaB	0.80
ATPC	0.91	PETC	1.99	PsbC (CP43)	0.21	PsaC	0.70
ATPD	1.08	PetD	1.69	PsbD (D2)	0.21	PSAD	0.95
AtpE	0.86	PETM	1.88	PsbE	0.15	PSAE	0.57
AtpF	1.06	PETO	0.81	PsbF	0.10	PSAF	0.96
ATPG	1.03	**Median**	**1.87**	PsbH	0.005	PSAG	1.06
AtpI	0.82			PsbJ	0.42	PSAH	0.88
**Median**	**1.03**			PsbL	0.19	PsaJ	0.30
				PBA1	1.26	PSAK	0.75
				**Median**	**0.15**	PSAL	0.90
				PSBO	0.26	PSAN	0.50
				PSBP1	0.45	**Median**	**0.84**
				PSBP3	14.83	LHCA1	0.81
				PSBP4	1.46	LHCA2	0.60
				PSBP6	1.96	LHCA3	0.89
				PSBQ	0.35	LHCA4	0.35
				PSBR	0.27	LHCA5	0.89
				**Median**	**0.45**	LHCA6	0.42
				LHCB4	0.61	LHCA7	1.12
				LHCB5	1.24	LHCA8	1.26
				LHCB7	5.13	LHCA9	0.62
				LHCBM1	0.58	**Median**	**0.81**
				LHCBM3	0.47		
				LHCBM5	0.55		
				LHCBM6	0.46		
				LHCBM8	0.44		
				LHCBM9	0.43		
				**Median**	**0.55**		

Values are based on the summed ion intensities in all gel bands of 3 biological replicates each of WT and mutant.

Median values are shown in bold.

In contrast to the ATP synthase and the Cyt *b_6_f* complex, the composition of PSI differed between WT and mutant. While, in WT, only a single PSI-LHCI complex with 11 detected core subunits and 9 LHCAs was observed, the mutant showed 2 prominent PSI-LHCI complexes that differed by the presence or absence of LHCA4 and LHCA6, which accumulated to less than half of the median levels of the other LHCAs (Fig. 6A; Table 1). As observed previously (Spaniol et al. 2022), some PSAH and all PSAN accumulated as unassembled subunits in both, *psb28* mutant and WT, presumably because they lost connection to the PSI core during sample preparation or electrophoresis. The median abundance of PSI core subunits and LHCI antennae was ∼16% and 19% lower in the mutant compared with the WT (Table 1).

The most dramatic change between *psb28* and WT was at the level of larger PSII complexes, with supercomplexes, dimers, and monomers/RC47 accumulating in the mutant only to 1%, 6%, and 27%, respectively, of WT levels, as judged from the median abundance of the core subunits in the complexes (Table 2; Fig. 6A; Supplementary Fig. S12). In contrast, D1 and D2 in RCII accumulated to more than 30-fold and CP43 in the CP43_mod_ to 10.5-fold higher levels in the mutant compared with the WT. D1 in D1_mod_ and PsbE in unassembled PsbE/F also accumulated 2.2- and 15.5-fold in the mutant, respectively. Overall, the median abundance of PSII core subunits in the *psb28* mutant was only ∼15% of that in the WT (Table 1). Even less CP47 and PsbH (∼8% and ∼0.5%, respectively, of WT levels) accumulated in the mutant, in line with their substantially lower synthesis rates (Fig. 3). In contrast, the previously identified PUTATIVELY PHOTOSYSTEM B ASSOCIATED 1 (PBA1) (Spaniol et al. 2022) accumulated to 1.26-fold higher levels in the mutant compared to the WT, indicating that its abundance is not coregulated with the canonical PSII core subunits. Except for PSBO, all other subunits involved in stabilizing/shielding the Mn_4_CaO_5_ cluster were found to migrate as unassembled subunits in both, *psb28* mutant and WT, presumably because they got detached from PSII during sample preparation or electrophoresis (Fig. 6A). The median abundance of all subunits of the water-splitting complex reached ∼45% of WT levels (Table 1). Only PSBP3, 4, and 6 behaved differently and were 14.83-, 1.46-, and 1.96-fold more abundant, respectively, in the mutant than in the WT. Hence, like PBA1, these 3 proteins are not coregulated with the other PSII subunits. PSBP3 is homologous to CyanoP and PSBP-LIKE PROTEIN 1 (PPL1) in *Synechocystis* and Arabidopsis, respectively, where these proteins were shown to facilitate PSII assembly (Knoppová et al. 2016; Che et al. 2020). The mean abundance of LHCII proteins in the mutant reached only ∼55% of the values of the WT (Table 1), and since hardly any larger PSII complexes were made in the mutant, it must contain a large pool of unassembled LHCII trimers and monomers. Only LHCB5 (CP26) and the recently identified LHCB7 protein (Klimmek et al. 2006) accumulated ∼1.24- and 5.13-fold, respectively, in the mutant when compared with the WT. In contrast to LHCB4 (CP29) and LHCB5, LHCB7 accumulated in the WT only in the unassembled form (Fig. 6A), as observed previously (Spaniol et al. 2022).

**Table 2.** Ratio of subunit abundance in various PSII assembly states between *psb28* mutant and WT

Subunit	SC	Dimers	Monomers/ RC47	RCII	CP43_mod_	D1_mod_/PsbE/F
PsbA (D1)	0.01	0.09	0.32	32.2	ND	2.2
PsbB (CP47)	0.01	0.07	0.28	ND	ND	nd/ND
PsbC (CP43)	0.02	0.09	0.24	ND	10.5	ND
PsbD (D2)	0.03	0.12	0.4	77.1	ND	ND
PsbE	0.02	0.05	0.26	ND	ND	15.5
PsbF	nd	nd	0.5	nd/ND	nd/ND	ND
PsbH	nd	nd	0.11	nd/ND	nd/ND	nd/ND
PsbJ	0.01	0.15	0.14	nd/ND	nd/ND	nd/ND
PsbL	nd	0.01	0.09	ND	nd/ND	nd/ND
PSBO	0.01	0.02	nd	nd	nd	0.6
PBA1	nd	nd/ND	0.83	nd/ND	nd/ND	1.3
**Median**	**0.01**	**0.06**	**0.27**			
Gel bands	7–11	13–15	17/18	22/23	25/26	28–30

Values are based on summed ion intensities in the bands indicated.

Median values are shown in bold.

ND, nd, not detected in WT, mutant (ion intensity < 0.05% of total intensity in respective strain); RCII, reaction centers; SC, supercomplexes.

When comparing MS data of isolated thylakoids (Table 1) with whole cell immunoblot data (Fig. 1, D and E), we detected relatively more Cyt *b_6_f* and PSI in the *psb28* mutant than in the WT, but less PSII and LHCII. While we cannot exclude the possibility that the growth conditions used for the 2 data sets varied slightly (e.g. culture volume and perceived light), these differences could also be due to an unequal extractability of thylakoid membranes caused by the differences in thylakoid structure and composition between the mutant and the WT (Fig. 2C).

### The migration patterns of several known PSII auxiliary factors differ between the *psb28* mutant and the WT

**Figure 7.**
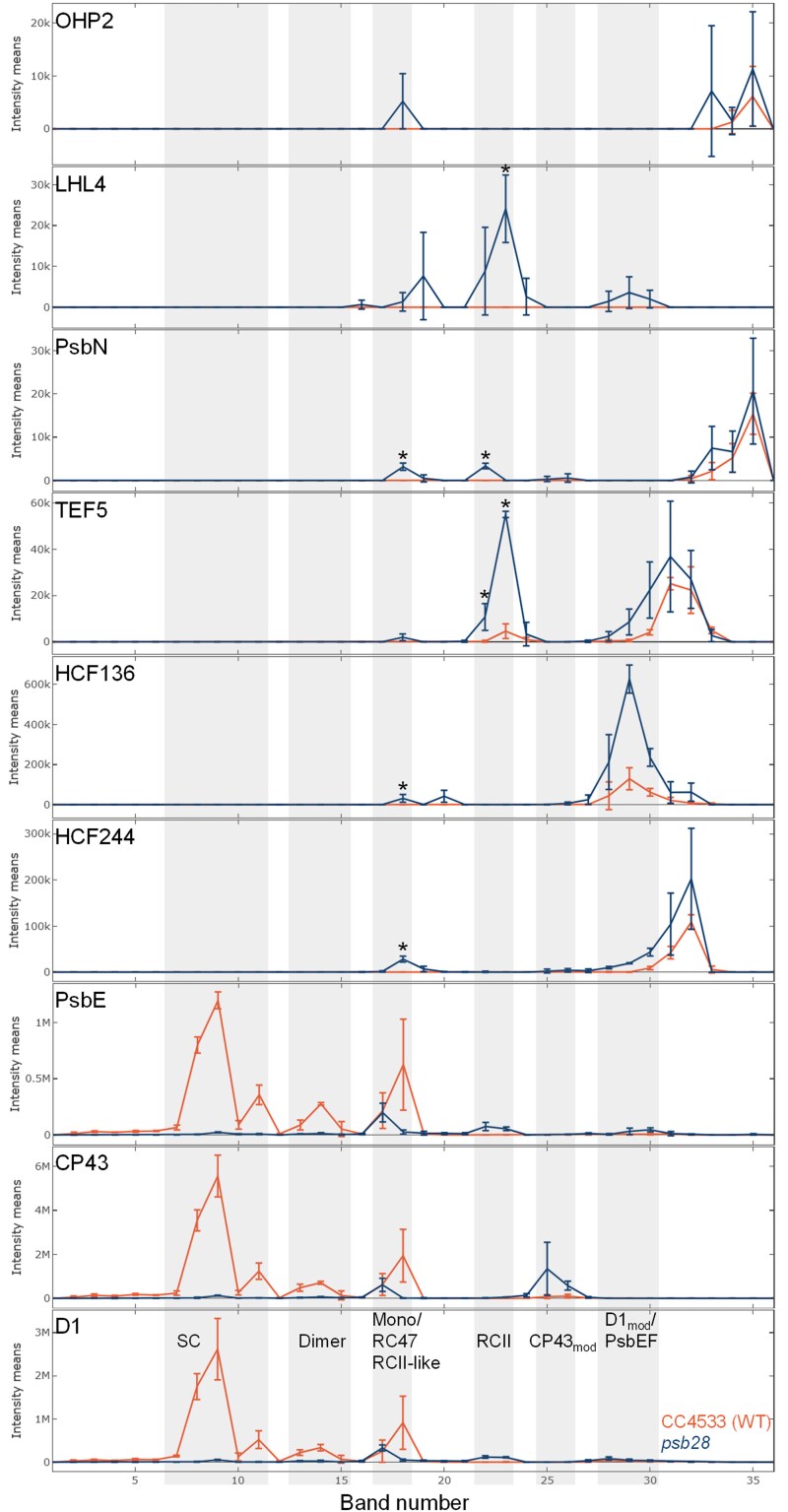
BN–PAGE migration profiles of PSII core subunits and PSII-associated proteins. Values for each protein are derived from averaged peptide ion intensities from 3 biological replicates. Error bars represent Sd. Individual profiles from each replicate before and after normalization and statistical analyses can be accessed in Supplementary Data Set S2. Asterisks indicate significant differences in ion intensities between WT (red) and *psb28* mutant (blue) in bands containing complexes larger than CP43_mod_ (2-tailed unpaired *t*-test, *P* < 0.05). SC, supercomplexes.

We next asked whether known PSII auxiliary factors would accumulate in complex with the accumulating early PSII assembly intermediates in the *psb28* mutant. To investigate this, we started out from a list of PSII auxiliary factors compiled by Lu (2016) for Arabidopsis. We also included PSII auxiliary factors identified later in chloroplasts and cyanobacteria, i.e. STROMAL SUPPRESSOR OF TIC40 PROTEIN 2 (Stolle et al. 2024), DECREASED ELECTRON TRANSPORT AT PSII/FACILITATOR OF PSBB BIOGENESIS 1 (Keller et al. 2023; Zhang et al. 2024), RESISTANCE TO PHYTOPHTORA 1 (Che et al. 2024), THYLAKOID RHODANESE-LIKE 2 (Li et al. 2023), ONE-HELIX PROTEIN (OHP) 1 and 2 (Beck et al. 2017), RBD1/RubA (Garcia-Cerdan et al. 2019; Kiss et al. 2019; Che et al. 2022), ACCLIMATION OF PHOTOSYNTHESIS TO THE ENVIRONMENT 1 (APE1)/Slr0575 (Myouga et al. 2018; Knoppova et al. 2022), Psb35 (Pascual-Aznar et al. 2021) as well as TEF14, and PHOTOSYSTEM II REPAIR FACTOR (PRF) 1 and 2 (Li et al. 2024). With this extended list of factors, we searched for Chlamydomonas homologs that were detected with 3 replicates each in the *psb28* mutant and the WT in our CP data set. This resulted in 31 factors, all of which overaccumulated in *psb28* compared to the WT, except for SIGNAL RECOGNITION PARTICLE 43, ALBINO 3.1, TEF30, LPA2, and PRF2 (Supplementary Table S1). The heat map of the migration profiles in Fig. 6B shows that most of the 31 factors migrated in the low molecular mass region below CP43_mod_. Of the factors found in assemblies migrating above CP43_mod_, we found 9 to display significant differences in at least 1 gel band (*P* < 0.05) between mutant and WT (Figs. 6B and 7; Supplementary Fig. S13): STATE TRANSITION 7-LIKE 1 (STL1), FTSH1, FTSH2, TEF30, HCF244, HCF136, TEF5, OHP2, and PsbN. TEF30 mediates the assembly of CP43 into RC47 and stabilizes PSII monomers in an immature intermediate state to prevent the premature association of peripheral antennae (Bhuiyan et al. 2015; Muranaka et al. 2016; Liu et al. 2024). We did not find TEF30 migrating with PSII monomers in the *psb28* mutant, suggesting that there are no PSII monomers capable of binding TEF30 (Fig. 6B). STL1 and FTSH1/2 accumulated in the *psb28* mutant above WT levels in several gel bands with higher molecular mass complexes (Supplementary Fig. S13). STL1 is homologous to STATE TRANSITION 8 in Arabidopsis, which phosphorylates PSII core subunits as well as PROTON GRADIENT REGULATOR 1A to regulate cyclic electron flow (CEF) (Bonardi et al. 2005; Reiland et al. 2011). Although STL1 has not been characterized in Chlamydomonas, a role in CEF regulation might be conserved (Longoni and Goldschmidt-Clermont 2021). The very similar migration pattern in large molecular mass complexes of FTSH1 and FTSH2 (Fig. 6B; Supplementary Fig. S13) confirms their presence in heterooligomers and their higher abundance in the *psb28* mutant points to a role of this thylakoid membrane protease in the degradation of misassembled PSII complexes (Malnoe et al. 2014).

In contrast to STL1 and FTSH1/2, all other PSII auxiliary factors accumulating in complexes above CP43_mod_ at significantly higher levels in the mutant compared with the WT comigrated with early PSII assembly intermediates: HCF244, HCF136, TEF5, OHP2, and PsbN in Band 18 with PSII monomers/RC47/RCII-like, and TEF5 and PsbN in Bands 22/23 with RCII (Fig. 7). No such peaks were observed for any of the 5 factors in the *lpa2* mutant (Supplementary Fig. S14). There might be some comigration of PsbN and HCF244 with CP43_mod_ and of HCF136, HCF244, and TEF5 with very small assemblies of D1 and PsbE/F. HCF244 (Ycf39), HCF136 (Ycf48), OHP2, and PsbN have been found in early PSII assembly intermediates with roles in PSII assembly in cyanobacteria and plants (Meurer et al. 1998; Plucken et al. 2002; Komenda et al. 2008; Link et al. 2012; Knoppova et al. 2014, 2022; Torabi et al. 2014; Beck et al. 2017; Hey and Grimm 2018; Li et al. 2018; Myouga et al. 2018; Maeda et al. 2022; Wang et al. 2023). In contrast, Arabidopsis PSB33/LIL8 (the TEF5 homolog in land plants) has been found to comigrate only with RC47 and larger PSII assemblies (especially monomers) (Fristedt et al. 2015, 2017; Kato et al. 2017; Nilsson et al. 2020).

### Identification of proteins potentially involved in early PSII assembly steps

To identify factors associated with early PSII assemblies, we searched in our CP data set for chloroplast proteins with similar migration properties as the 5 known PSII auxiliary factors HCF244, HCF136, TEF5, OHP2, and PsbN, i.e. proteins specifically accumulating in Bands 17/18 (PSII monomers/RC47/RCII-like) and/or 22/23 (RCII) in *psb28* but not in the WT. Four proteins met these criteria: Cre03.g154600 (3 peptides) and Cre01.g007700 (3 peptides) accumulated in Bands 17/18, and LHC-LIKE 4 (LHL4) (4 peptides) and Cre10.g450500 (3 peptides) accumulated both, in Bands 18 and 22/23 (Fig. 7; Supplementary Fig. S14). Cre01.g007700 and LHL4 were not detected in the *lpa2* CP data set. In that data set, Cre10.g450500 comigrated with PSII monomers and RC47, while Cre03.g154600 migrated between them, thus disqualifying Cre03.g154600 as a PSII-associated protein. LHL4 is closely related to PSBS and uniquely found in green microalgae (Dannay et al. 2025). Cre01.g007700 encodes an aminopeptidase and Cre10.g450500 has a starch-binding domain; both are yet uncharacterized in Chlamydomonas.

### The *tef5* mutant has a lower PSII content than the WT and shows impaired growth in high light and under photoautotrophic conditions

**Figure 8.**
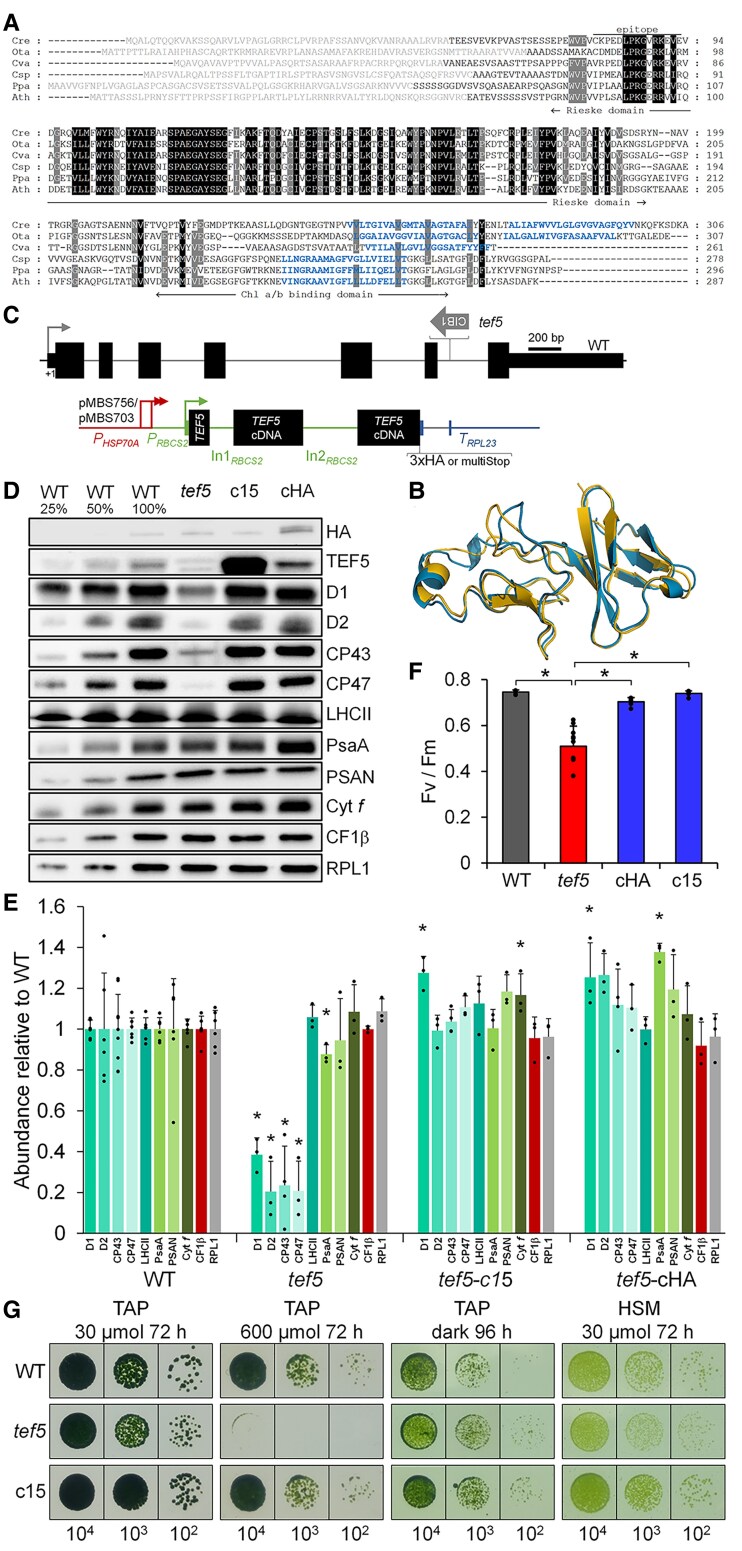
Phenotypes of the *tef5* mutant compared to WT and complemented lines. **A)** Alignment of amino acid sequences of algal and land plant homologs of TEF5/PSB33/LIL8. Residues highlighted in black and gray are conserved in 6 and 5 of the sequences, respectively. Predicted chloroplast transit peptides are shown in gray font and predicted transmembrane helices in blue. The epitope from Chlamydomonas TEF5 used for antibody production is indicated by a horizontal line. Cre, *C. reinhardtii* (Cre09.g411200); Ota, *O. tauri* (XP_003078526); Cva, *C. variabilis* (XP_005846469); Csp, *Closterium* sp. (CAI5958768); Ppa, *Physcomitrium patens* (XP_024377109); Ath, *A. thaliana* (AT1G71500). **B)** Pairwise structure alignment of the AlphaFold structures of the Rieske-like domains from Arabidopsis PSB33 (gold) and Chlamydomonas TEF5 (blue). **C)** Structure of the Chlamydomonas *TEF5* gene, insertion site of the CIB1 cassette (thick gray arrow) in the *tef5* mutant, and constructs for complementation. Protein coding regions are drawn as black boxes, UTRs as bars, and introns and promoter regions as thin lines. Sequences of the *HSP70A* promoter are drawn in red and sequences of the *RBCS2* promoter, 5′ UTR, and introns (In1 and In2) are drawn in green. *RPL23* terminator sequences are drawn in blue. Thin arrows indicate transcriptional start sites. pMBS756 contains sequences encoding for a 3xHA tag whereas pMBS703 contains sequences encoding for 3 consecutive stop codons (MultiStop). **D)** Immunoblot analysis of the accumulation of TEF5 and of subunits of the major thylakoid membrane protein complexes. c15 and cHA are lines complemented with constructs pMBS703 and pMBS756, respectively, shown in **C)**. PSII, D1, D2, CP43, CP47, and LHCII; PSI, PsaA and PSAN; Cyt *b_6_f* complex, Cyt *f*; ATP synthase, CF1b. Ribosomal protein RPL1 served as loading control. Ten micrograms of whole-cell proteins (100%) were analysed. **E)** Quantification of the immunoblot analysis shown in **D)**. Values are means from 3 independent experiments normalized first by the median of all signals obtained with a particular antiserum in the same experiment and then by the mean signal of the WT. Error bars represent Sd. Asterisks indicate significant differences with respect to the WT (2-tailed, unpaired *t*-test with Bonferroni–Holm correction, *P* < 0.05). The absence of an asterisk means that there were no significant differences. **F)**  *F*_v_/*F*_m_ values of the *tef5* mutant versus WT and complemented lines. Shown are averages from 10 and 6 independent experiments for *tef5* and the other lines, respectively, each measured with 3 technical replicates. Error bars represent Sd. Asterisks indicate significant differences with respect to *tef5* (2-tailed, unpaired *t*-test with Bonferroni–Holm correction, *P* < 0.001). **G)** Analysis of the growth of 10^4^ to 10^2^ spotted cells under the conditions indicated.

The comigration of a large portion of TEF5 with RCII and its comigration with HCF136, HCF244, OHP2, and PsbN in the *psb28* mutant suggested a possible role of TEF5 in PSII biogenesis, which was considered unlikely for its homolog PSB33/LIL8 in Arabidopsis (Fristedt et al. 2017; Kato et al. 2017). TEF5/PSB33/LIL8 are conserved in the green lineage, and there are no orthologs in cyanobacteria. They contain chloroplast transit peptides and share a Rieske-like domain lacking the residues required for the binding of mononuclear iron or an iron-sulfur cluster (Fristedt et al. 2015) (Fig. 8A). Moreover, they share 1 to 2 C-terminal transmembrane helices, where the loss of 1 transmembrane helix appears to have occurred before the evolution of land plants (Chlamydomonas and *Ostreococcus tauri* contain 2, while *Chlorella variabilis* and members of the Streptophytes contain only a single transmembrane helix; Fig. 8A). Although the Rieske-like domains of Chlamydomonas TEF5 and Arabidopsis PSB33/LIL8 share only 54% identical residues, their structures predicted by AlphaFold are very similar (RMSD = 1.08 Å, TM score = 0.93; Fig. 8B). To investigate a possible role of TEF5 in early steps of PSII assembly, we selected a *tef5* mutant from the CLiP collection (Li et al. 2016) that had the CIB1 mutagenesis cassette integrated into the sixth intron of the *TEF5* gene (Fig. 8C). Since we could amplify *TEF5* sequences of the expected sizes from both sides of the CIB1 cassette by PCR, there appear to be no larger deletions/rearrangements (Supplementary Fig. S15, A and B). Reverse transcription quantitative PCR (RT-qPCR) analysis revealed a ∼147-fold reduced abundance of *TEF5* transcript in the *tef5* mutant compared with the WT (Supplementary Fig. S15C). An antibody raised against a peptide from the N-terminal part of the TEF5 protein (Fig. 8A) specifically detected a protein band at the expected molecular mass of ∼27.5 kDa in the WT, which was absent in the *tef5* CLiP mutant (Fig. 8D; Supplementary Fig. S15D). In the *tef5* mutant, PSII core subunits accumulated to between 20% and 40% of WT levels while there was no or little change in the abundance of LHCII, PSI core subunits, ATP synthase subunit CF1β, and Cyt *f* (Fig. 8, D and E). We synthesized the *TEF5* cDNA sequence interrupted by the first 2 Chlamydomonas *RBCS2* introns, fused it with sequences encoding a C-terminal 3xHA tag or multiple stop codons, and placed it under control of the constitutive *HSP70A-RBCS2* promoter and the *RPL23* terminator using modular cloning (Fig. 8C). We combined the *TEF5* transcription unit with an *aadA* cassette and transformed it into the *tef5* mutant. Spectinomycin-resistant transformants obtained with both constructs were then screened by immunoblotting for the accumulation of HA-tagged TEF5 and/or for enhanced D1 accumulation (Supplementary Fig. S16). Five transformants accumulated HA-tagged TEF5 and all accumulated D1 to WT levels. One transformant (*tef5*-cHA) accumulated TEF5 transcripts to ∼73-fold higher levels than WT but TEF5 protein levels were not much higher than those in the WT (Fig. 8D; Supplementary Fig. S15C and D). Since the band detected with the TEF5 antibody in cHA had the same size as in WT, we assume that the 3xHA tag was removed from part of the protein, as was observed with PSB28-3xHA. A transformant generated with the construct encoding nontagged TEF5 (*tef5*-c15) accumulated TEF5 to much higher levels than the WT (Fig. 8D). Both HA-tagged and untagged transformants accumulated PSII subunits to WT levels and perhaps even beyond (Fig. 8, D and E; Supplementary Fig. S16, B and C). This came along with the restoration of WT *F*_v_/*F*_m_ values (Fig. 8F). The *tef5* mutant showed slightly reduced growth under photoautotrophic and mixotrophic conditions in low light (30 *µ*mol photons m^−2^ s^−1^) and a severe growth defect under mixotrophic conditions in high light (600 *µ*mol photons m^−2^ s^−1^), while growth under heterotrophic conditions in the dark was like WT (Fig. 8G). These growth phenotypes were fully restored in the *tef5*-c15 transformant.

### Chloroplast morphology is intact in the *tef5* mutant, but thylakoid membranes unstack more frequently

**Figure 9.**
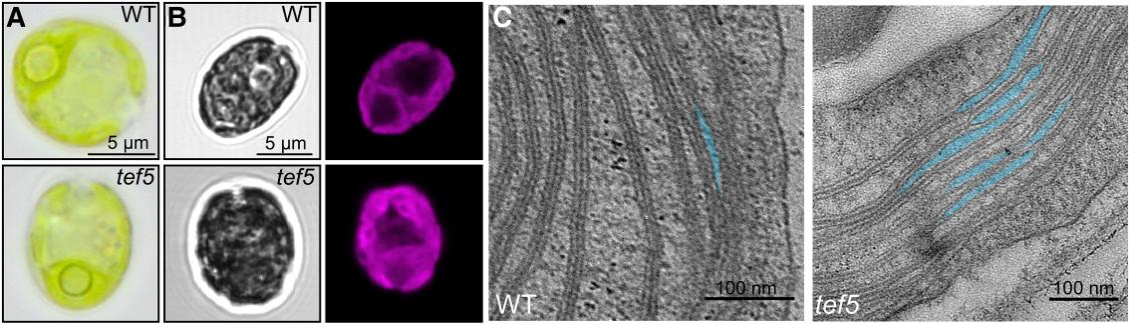
Light, fluorescence, and electron microscopy of the *tef5* mutant. **A)** Light microscopy images of WT and *tef5* mutant grown under mixotrophic conditions in low light (30 *µ*mol photons m^−2^ s^−1^). More images are shown in Supplementary Fig. S4A. **B)** Confocal laser-scanning fluorescence microscopy of a WT and *tef5* cell. Shown are bright-field images (left) and Chl autofluorescence (right). More images are shown in Supplementary Fig. S4B. **C)** Electron microscopy pictures of WT (left) and *tef5* mutant (right). Blue areas indicate regions where thylakoid stacking is interrupted. More images are shown in Supplementary Fig. S5. A single scale bar applies to all images displayed in that panel.

Light, fluorescence, and TEM were used to analyze possible changes in cell morphology and thylakoid ultrastructure in the *tef5* mutant. Light and fluorescence microscopy revealed no visible change in chloroplast morphology in the *tef5* mutant (Fig. 9, A and B; Supplementary Fig. S4). TEM revealed that thylakoid membranes in the mutant detached from stacks more frequently than the WT resulting in more regions with interrupted thylakoid stacking (shown in blue in Fig. 9C and Supplementary Fig. S5). We counted these regions in 10 cells each of WT and *tef5* mutant and found them to occur on average 3 ± 1 and 10 ± 5 times per *µ*m^2^ in WT and *tef5* cells, respectively, i.e. significantly more frequently in *tef5* (*P* < 0.001).

### The synthesis of CP47 and PsbH is reduced in the *tef5* mutant

**Figure 10.**
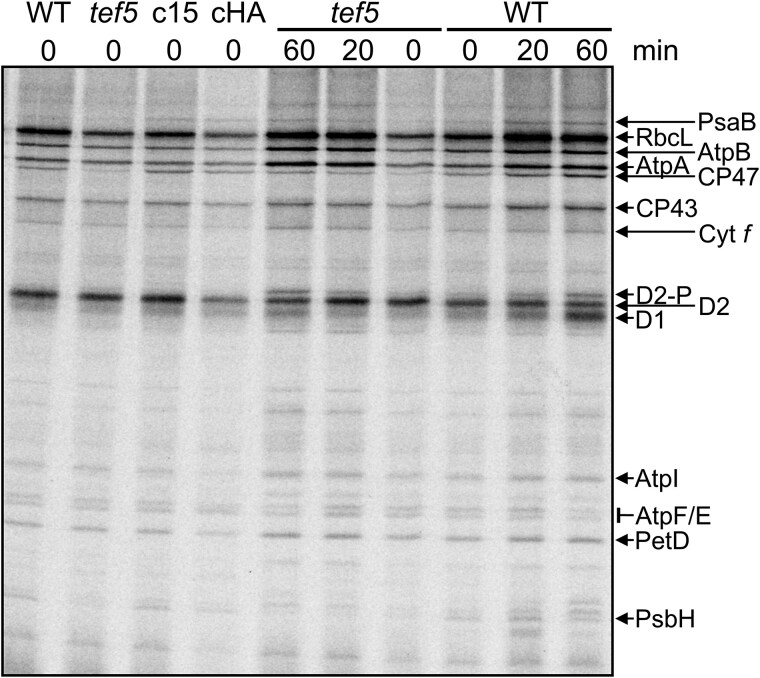
Pulse-chase analysis of synthesis and stability of thylakoid membrane proteins in the *tef5* mutant. WT, *tef5* mutant, and complemented lines c15 and cHA were labeled with ^14^C-acetate in low light (20 *µ*mol photons m^−2^ s^−1^) for 7 min in the presence of cytosolic translation inhibitor cycloheximide (0) and chased with unlabeled acetate for 20 and 60 min. Proteins were separated on a 12% to 18% SDS-urea gel and visualized by autoradiography.

To investigate whether the absence of TEF5 affects the synthesis and stability of PSII core subunits in the *tef5* mutant, we performed pulse-chase analyses with ^14^C-acetate. Comparing *tef5* with the WT, we defined reduced radioactivity in a protein after the 7-min pulse as indicative for reduced protein synthesis and reduced radioactivity during the 60-min chase as indicative for reduced protein stability. Based on these definitions, the synthesis of CP47 and PsbH and the stability of D1 were reduced in *tef5* compared with the WT (Fig. 10). CP47 and PsbH synthesis was restored to WT rates in the complemented lines.

### PSII assembly is impaired in the *tef5* mutant in the light but to a lesser extent in the dark

**Figure 11.**
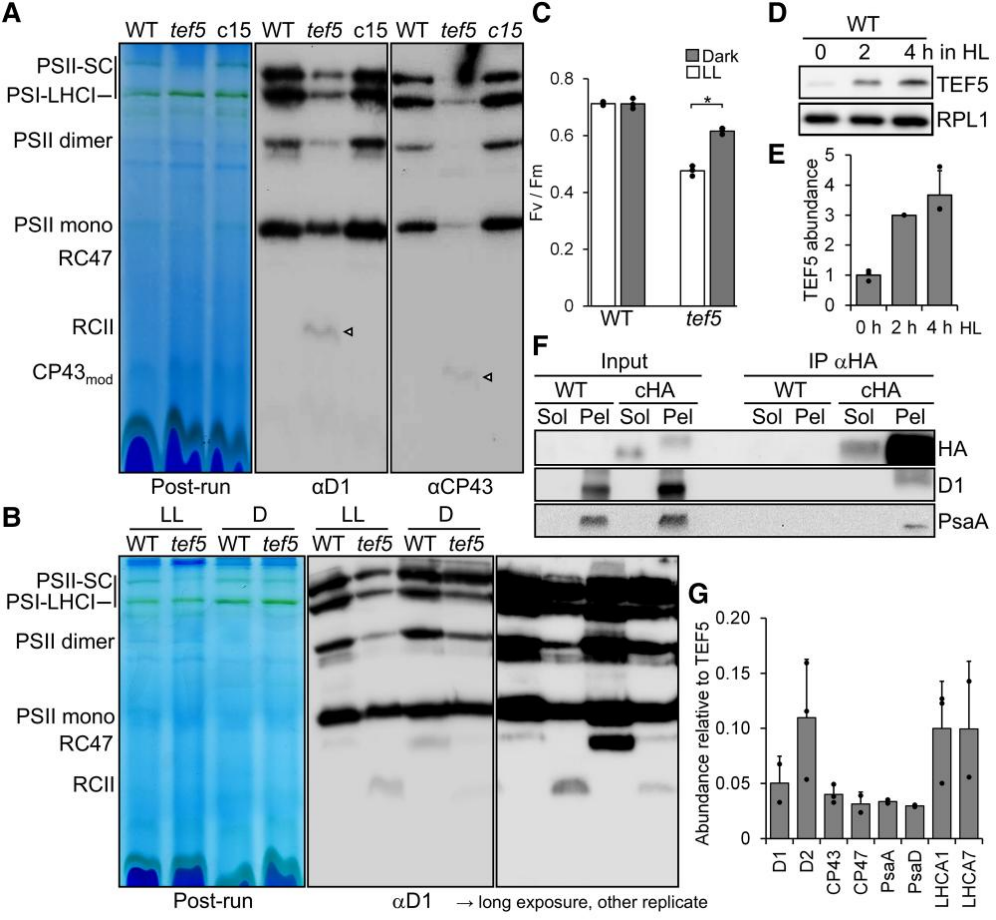
Analysis of protein complexes in the *tef5* mutant and of proteins interacting with TEF5. **A)** BN–PAGE analysis of proteins from cells grown in LL (30 *µ*mol photons m^−2^ s^−1^). Sixty micrograms of whole-cell proteins from WT, *tef5* mutant, and complemented line *tef5*-c15 were solubilized with 1% β-DDM and separated on a 4% to 15% BN gel. Shown is a picture of the gel after the run and an immunoblot detected with antibodies against D1 and CP43. Arrowheads point to faint bands likely representing RCII and CP43_mod_ in the *tef5* mutant. **B)** BN–PAGE analysis of proteins from WT and *tef5* mutant grown in LL (30 *µ*mol photons m^−2^ s^−1^) and in the dark (D) for 72 h. Whole-cell proteins were solubilized with 1% β-DDM and separated on a 4% to 15% BN gel. Shown is a picture of the gel after the run and an immunoblot detected with an antibody against D1 accompanied by a longer exposure of an independent replicate. **C)**  *F*_v_/*F*_m_ values of the *tef5* mutant versus WT grown in LL (30 *µ*mol photons m^−2^ s^−1^) and in the dark for 72 h. Shown are averages from 3 independent experiments. Error bars represent Sd. The asterisk indicates a significant difference between low-light versus dark-grown *tef5* cells (2-tailed, unpaired *t*-test, *P* < 0.001). **D)** Analysis of TEF5 accumulation in HL. WT was exposed to 1,200 *µ*mol photons m^−2^ s^−1^ for 4 h, and samples taken prior, 2, and 4 h after the treatment were analyzed by immunoblotting using the peptide antibody against TEF5 and an antibody against RPL1 as loading control. **E)** Quantification of the immunoblot analysis shown in **D)**. Values are means from 3 independent experiments. Normalization was done as described for Fig. 1D. Error bars represent Sd. **F)** Immunoprecipitation of TEF5. Cells from complemented line *tef5*-cHA were fractionated via freeze–thaw cycles and centrifugation. HA-tagged TEF5 was then immunoprecipitated (IP) from soluble (Sol) and membrane-enriched (Pel) fractions with an HA antibody. 1% of the input and 10% of the precipitate were analyzed by SDS–PAGE and immunoblotting using antibodies against HA, D1, and PsaA. **G)** MS-based quantification of PSI and PSII subunits coprecipitated from solubilized membrane fractions with HA-tagged TEF5. IBAQ values for each protein were normalized by the IBAQ value for TEF5. Shown are mean values from 2 to 3 independent replicates. Error bars represent Sd. HL, high light; LL, low light; SC, supercomplexes.

To assess how the reduced synthesis and accumulation of PSII core subunits in the *tef5* mutant affects PSII complex assembly, we analyzed whole-cell proteins from the low light-grown WT, the *tef5* mutant, and the complemented line *tef5*-c15 by BN–PAGE and immunoblotting using antibodies against D1 and CP43. As shown in Fig. 11A, we found much weaker signals for PSII supercomplexes, dimers, and monomers in the mutant when compared with WT and the complemented line. In contrast, the *tef5* mutant accumulated RCII and CP43_mod_, which was not the case in the WT and the complemented line.

To investigate whether the impaired assembly of PSII in the *tef5* mutant was due to an effect of light, we compared protein complexes in solubilized whole-cell extracts from the WT and the *tef5* mutant grown in low light and in the dark for 72 h by BN–PAGE and immunoblotting using a D1 antibody. We clearly observed stronger signals for PSII dimers and supercomplexes in the dark-grown versus the light-grown *tef5* mutant (Fig. 11B). Moreover, the mutant accumulated less RCII in the dark than in the light. Most interestingly, the mutant accumulated no RC47 in the light. In the dark, the WT accumulated large amounts of RC47 and the *tef5* mutant accumulated some. The partially rescued PSII assembly in the dark-grown mutant was also reflected at the level of *F*_v_/*F*_m_ values, which were significantly higher in the dark-grown versus low light-grown mutant (0.62 vs. 0.48, *P* < 0.001) but did not reach values obtained for the dark-grown WT (0.72) (Fig. 11C).

The potential role of TEF5 in PSII assembly at the RCII/RC47 level suggests that TEF5 may also be involved in the repair of photodamaged PSII. We therefore first tested whether TEF5 accumulates in cells exposed to high light and found a ∼3.7-fold increased abundance of TEF5 protein after 4-h exposure to 1,200 *µ*mol photons m^−2^ s^−1^ (Fig. 11, D and E). This was surprising, since PSB33/LIL8 was reported to be expressed constitutively (Kato et al. 2017). To investigate the susceptibility of PSII in the *tef5* mutant to high light and its capability to recover functional PSII, we exposed the WT, the *tef5* mutant, and the complemented lines to high light (1,800 *µ*mol photons m^−2^ s^−1^) in the presence of CAP for 1 h and allowed cells to recover in the presence and absence of CAP at low light (30 *µ*mol photons m^−2^ s^−1^). All 4 lines recovered 86% to 96% of initial PSII activity (and most of D1) at similar rates within 6.5 h in a protein synthesis-dependent manner (Supplementary Fig. S17). Like the *psb28* mutant, the *tef5* mutant lost PSII activity upon sulfur starvation faster than the WT and the complemented lines but recovered initial PSII activity (and D1 levels) with similar rates as the other lines (Supplementary Fig. S18). In summary, the *tef5* mutant is impaired in PSII assembly presumably at the step where the CP47_mod_ combines with RCII to RC47. As observed for the *psb28* mutant, the low levels of PSII made in the *tef5* mutant are susceptible to photoinhibition and degradation upon sulfur deprivation but can be fully recovered to these low levels at WT rates.

### TEF5 interacts with subunits of PSI and PSII

The comigration of TEF5 with early PSII assembly intermediates in the *psb28* mutant and its potential role in PSII assembly implies its direct interaction with PSII. To test this, we used the HA antibody to immunoprecipitate TEF5-3xHA from soluble and membrane-enriched fractions from the complemented *tef5*-cHA line. Prior to immunoprecipitation, complexes were stabilized by in vivo cross-linking with 0.37% formaldehyde. As shown in Fig. 11F, most of TEF5-3xHA was precipitated from the membrane-enriched fraction, but some was also precipitated from the soluble fraction. The different migration behavior of TEF5 in soluble and membrane-enriched fractions could be due to the presence of large amounts of LHCII at the same position in the gel only in the membrane-enriched fraction, which has been observed also for Arabidopsis PSB33/LIL8 (Kato et al. 2017). D1 and PsaA were coprecipitated with TEF5 only in the membrane-enriched fraction. To identify and quantify all proteins interacting with TEF5, we analyzed the TEF5 immunopreciptates by LC-MS/MS. In line with the immunoblot data, we detected ∼16 times more TEF5 in the membrane-enriched fraction than in the soluble fraction (Supplementary Data Set S4). Among the proteins detected with TEF5 in at least 2 replicates in the membrane fraction, we found PSII subunits D1, D2, CP43, and CP47 as well as PSI subunits PsaA, PsaD, LHCA1, and LHCA7 (Fig. 11G; Supplementary Data Set S4). We also detected RBCS2, a transporter and an ATP synthase subunit from mitochondria, and a putative transhydrogenase, guanylate cyclase, and nucleolar protein, which most likely are contaminants. IBAQ normalized to TEF5 revealed that D2 is the most prominent TEF5 interaction partner, followed by LHCA1/7, D1, CP43, PsaA, CP47, and PsaD (Fig. 11G).

To obtain a structural hypothesis of how TEF5/PSB33 could interact with the PSII core, we used AlphaFold3 (Abramson et al. 2024) with Chlamydomonas TEF5 and Arabidopsis PSB33 and PSII models constructed with D1, D2, PsbE, PsbF, and PsbI to represent RCII and with D1, D2, PsbE, PsbF, PsbI, CP47, CP43, and PsbH to represent a PSII monomer, albeit incomplete. While Alphafold3 failed to predict a stable interaction of TEF5/PSB33 with RCII, it predicted a strong interaction with CP47 in the PSII monomer, complemented with few interactions with PsbH (Supplementary Fig. S19). Here, the Rieske-like domain points into the stroma, consistent with what has been determined experimentally (Fristedt et al. 2015). To test if this model is biologically meaningful, we introduced point mutations into TEF5 encoded by our complementation vector pMBS703 (Fig. 8C) that would disrupt the interaction of TEF5 with CP47/PsbH (F170A, F216K, Y223A, W282A, and W282D, Supplementary Fig. S20). All transformants producing TEF5 with single point mutations, double mutations (Y223A/W282A, F170A/F216K), triple mutation (F170A/F216K/Y223A), or quadruple mutation (F170A/F216K/Y223A/W282A) assembled PSII normally, as assessed by the accumulation of D1, CP47, and CP43 to WT levels and WT values for *F*_v_/*F*_m_ (Supplementary Fig. S21). These results suggest either that the AlphaFold3 model is incorrect or that the interaction of TEF5/PSB33 with CP47/PsbH is not required for its function in PSII assembly.

## Discussion

### Psb28 is of much greater importance for PSII assembly in Chlamydomonas than in *Synechocystis*

Chlamydomonas PSB28 has several traits in common with cyanobacterial Psb28. Cyanobacterial Psb28 is substoichiometric to PSII and interacts only transiently with PSII, mainly with RC47 and less with PSII monomers, while most Psb28 is present as free protein (Kashino et al. 2002; Dobakova et al. 2009; Boehm et al. 2012; Nowaczyk et al. 2012; Sakata et al. 2013; Beckova et al. 2017; Xiao et al. 2021; Zabret et al. 2021). Similarly, Chlamydomonas PSB28 is ∼78-fold less abundant than PSII and in CP was only found as free protein in WT but comigrated mainly with RC47 and less with PSII monomers in the *lpa2* mutant, which overaccumulates RC47 (Spaniol et al. 2022). In a complemented line, HA-tagged PSB28 comigrated more with PSII monomers than with RC47 and a fraction of tagged PSB28 was present as free protein (Fig. 4A). Accordingly, immunoprecipitation of tagged PSB28 revealed D2 and D1 as the most prominent interaction partners, followed by CP47 and CP43 (Fig. 4, B and C). Functional similarity between Chlamydomonas and cyanobacterial Psb28 was indicated by the similarity of the predicted structure of Chlamydomonas PSB28 with cyanobacterial Psb28 (Fig. 1B) and by the ability of *Synechocystis* Psb28-1 to partially complement the Chlamydomonas *psb28* mutant (Fig. 5). Common is the reduced synthesis of CP47 and PSI/PsaB in *Synechocystis* and Chlamydomonas *psb28* mutants (Dobakova et al. 2009; Beckova et al. 2017); however, in Chlamydomonas, the synthesis of D1, D2, CP43, and PsbH was affected, too (Fig. 3). This points to control by epistasis of synthesis (CES) of PSII subunits in the *psb28* mutant (Minai et al. 2006), possibly by a negative feedback regulation effected by accumulating assembly intermediates such as RCII and CP43_mod_ (Figs. 4A and 6A; Table 2).

One difference between *Synechocystis* Psb28-1 and Chlamydomonas PSB28 is that the abundance of Psb28-1 did not increase under high light intensities (Beckova et al. 2017), whereas the abundance of PSB28 increased ∼2.9-fold (Fig. 4, E and F). Moreover, *Synechocystis* Psb28-1 and 2 were found in PSII-PSI supercomplexes particularly under high light intensities (Beckova et al. 2017), which we did not observe in Chlamydomonas (Fig. 4D). In Chlamydomonas, more PSB28 interacted with PSII monomers and particularly with RC47 in high versus low light (Fig. 4D), suggesting a role of PSB28 also in PSII repair in this alga.

Probably most surprising are the differences in the phenotypes of the *Synechocystis* and Chlamydomonas *psb28* mutants: the *Synechocystis psb28-1* mutant accumulated fully functional PSII, and growth phenotypes were observed only at higher temperatures and high or fluctuating light exposure (Dobakova et al. 2009; Sakata et al. 2013; Beckova et al. 2017). In contrast, the Chlamydomonas *psb28* mutant could not grow photoautotrophically (Fig. 1H) and accumulated PSII supercomplexes, dimers, and monomers only to 1%, 6%, and 27% of WT levels, respectively, while it overaccumulated RCII and CP43_mod_ (Table 2; Figs. 4A and 6A). PSII outer antennae were reduced by ∼45% and PSI/LHCI by 16% to 19%, compared with WT. Levels of the ATP synthase were unaltered in the mutant, while the Cyt *b_6_f* complex overaccumulated between 1.4- and 1.9-fold (Table 2; Fig. 1, D and E). These dramatic changes in the photosynthetic apparatus probably cause the strongly reduced thylakoid stacking and the distorted shape of the chloroplast (Fig. 2; Supplementary Figs. S4 and S5). The reduced levels of PSI, increased levels of Cyt *b_6_f*, and the distorted chloroplast shape are unusual phenotypes for PSII mutants in Chlamydomonas: the *ohp2* mutant, lacking PSII, accumulates PSI and Cyt *b_6_f* at WT levels (Wang et al. 2023) as do the *lpa2* and *tef5* mutants, with PSII levels reduced by about half and below 40% of the WT levels, respectively (Spaniol et al. 2022) (Fig. 8, D and E). Likewise, the *2pac* mutant, lacking RBD1 and accumulating CP43 and D2 to very low levels, exhibited WT levels of PSI (Garcia-Cerdan et al. 2019). While some changes in thylakoid structure were observed in the *lpa2* and *tef5* mutants, the morphology of the chloroplast was unaltered (Spaniol et al. 2022) (Fig. 9). The reason for these pleiotropic phenotypes of the *psb28* mutant could be an additional function of PSB28 besides that as a PSII assembly factor. Indeed, *Synechocystis* Psb28-1 was proposed to play a role in regulating Chl incorporation into CP47 and PSI (Beckova et al. 2017). Alternatively, PSII assembly intermediates specifically accumulating in the *psb28* mutant might act as regulators of other processes resembling CES (Choquet and Wollman 2023). It is also possible that PSII intermediates in the *psb28* mutant specifically bind assembly factors, chaperones or proteases, which are then not sufficiently available for other chloroplast processes.

The fact that PSII accumulation is much more affected in the Chlamydomonas *psb28* mutant than in the *Synechocystis psb28* mutant is probably due to the very efficient proteolytic degradation of nonassembled complex subunits and misassembled complexes in Chlamydomonas (Choquet and Wollman 2023). Here, the FTSH protease most likely is a key player, since Chlamydomonas *ccb* mutants, normally lacking the Cyt *b_6_f* complex, accumulated aberrant forms of Cyt *b_6_f* in the *ftsh1-1* mutant background (Malnoe et al. 2014). Moreover, the *2pac* mutant, presumably impaired in the delivery of the nonhaem iron to PSII and accumulating PSII core subunits at very low levels, accumulated PSII core subunits at much higher levels in the *ftsh1-1* background (Calderon et al. 2023). Accordingly, FTSH1/2 were ∼2.5-fold more abundant in *psb28* than in WT (Supplementary Table S1), and FTSH1/2 complexes were more abundant in membranes at higher molecular mass ranges (Fig. 6B; Supplementary Fig. S13). Another explanation for the impaired accumulation of larger PSII assemblies in the Chlamydomonas *psb28* mutant versus the *Synechocystis psb28* mutant is that the conformational changes introduced by Psb28 into the PSII core (Xiao et al. 2021; Zabret et al. 2021) are more important for correct assembly of the CP43_mod_ into RC47 in Chlamydomonas than in *Synechocystis*. It was proposed that the conformational changes introduced by Psb28 might also protect premature PSII from photodamage (Xiao et al. 2021; Zabret et al. 2021). Since the problem in PSII assembly prevailed in the dark-grown Chlamydomonas *psb28* mutant (Supplementary Fig. S6A), the impaired accumulation of larger PSII assemblies in the mutant is unlikely to be caused by enhanced photodamage to early PSII assemblies.

The dependence of PSII assembly on auxiliary factors generally appears to be stronger in chloroplasts than in cyanobacteria. Examples for this, in addition to PSB28, are HCF136 (Ycf48 in cyanobacteria), HCF244 (Ycf39 in cyanobacteria), PsbN, and PAM68. While the absence of these factors resulted in severe PSII assembly defects in Arabidopsis or tobacco, *Synechocystis* mutants lacking these factors could assemble functional PSII (Mayers et al. 1993; Meurer et al. 1998; Komenda et al. 2008; Armbruster et al. 2010; Link et al. 2012; Knoppova et al. 2014; Torabi et al. 2014). An exception to this trend is RBD1/RubA, whose absence results in severe PSII assembly defects in Arabidopsis, Chlamydomonas, and *Synechocystis* (Garcia-Cerdan et al. 2019; Kiss et al. 2019; Che et al. 2022).

### CP confirms PBA1 and CGLD16 as PSII-associated proteins

Previously, CP of thylakoid membranes of the WT and the *lpa2* mutant identified PBA1 and CONSERVED IN THE GREEN LINEAGE AND DIATOMS 16 (CGLD16) as PSII-associated proteins (Spaniol et al. 2022). Both contain predicted single transmembrane helices and chloroplast transit peptides and have predicted mature masses of 6.4 and 7.9 kDa, respectively. PBA1 is present only in members of the green algae, brown algae, diatoms, and Eustigmatophytes, while CGLD16 is conserved in the green lineage and diatoms. CGLD16 comigrated with PSII monomers and RC47 in the WT and the *lpa2* mutant (Spaniol et al. 2022), and we found the same migration pattern for CGLD16 also for the WT and the *psb28* mutant in this work (Supplementary Fig. S14). In the WT, PBA1 comigrated with PSII supercomplexes, dimers, monomers, and RC47 and its abundance in these complexes was reduced in the *lpa2* mutant, where the unassembled form was more abundant (Spaniol et al. 2022). In this work, we found PBA1 to comigrate with PSII supercomplexes only in the WT and with PSII monomers/RC47 in both WT and *psb28* (Fig. 6A; Supplementary Fig. S12). These data confirm that PBA1 and CGLD16 might be PSII-associated proteins. Indeed, PBA1 was recently identified in a cryo-EM structure of a PSII repair intermediate where it was termed PRF2′ and found to induce conformational changes at the DE-loop of D1 (Li et al. 2024).

### CP confirms previously identified PSII assembly factors and identifies factors with potential roles in early PSII assembly

**Figure 12.**
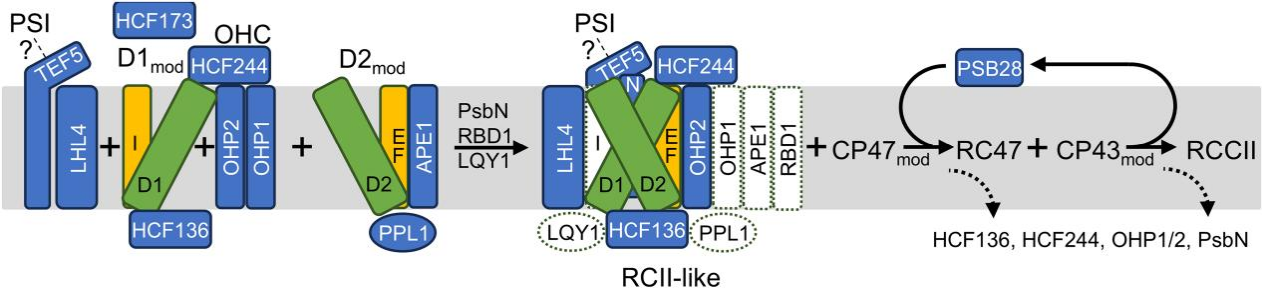
Working model for the place of action of TEF5 and PSB28 during PSII assembly. The formation of the RCII complex from D1_mod_ (D1, PsbI, and HCF136/Ycf48) and D2_mod_ (D2, PsbE, F, PPL1/CyanoP, and APE1/Slr0575) is facilitated by the OHC complex consisting of HCF244 and OHP1/2 in chloroplasts and Ycf39 and HliC/D in *Synechocystis* with roles in photoprotection and Chl delivery. TEF5, possibly with LHL4 and/or PSI, could play a similar role as the OHC in photoprotection of RCII and pigment delivery or introduce a conformational change required for the correct assembly of CP47. PsbN, RBD1/RubA, and LQY1 join the RCII-like complex for further maturation. The colored proteins in the RCII-like complex were found to comigrate in Band 18 of the CP experiment in the *psb28* mutant. The white proteins were shown to be part of RCII-like in other studies. The RCII-like complex fuses with the CP47_mod_ to form RC47. PSB28 and other factors further mature RC47 for fusion with the CP43_mod_ to form RCCII. Several assembly factors leave PSII during further assembly steps. Factors with roles in PSII assembly are shown in blue, Chl-containing core subunits are shown in green, and Chl-less core subunits in orange.

CP of the thylakoid membranes of WT and *psb28* revealed 31 PSII auxiliary factors in Chlamydomonas that were identified in all 3 replicates of WT and mutant and have been described in previous studies in cyanobacteria and land plants (Supplementary Table S1). Among these, 26 accumulated to higher levels in the mutant compared to WT, potentially to compensate for impaired PSII accumulation in the mutant. Six proteins were found to comigrate with early PSII assembly intermediates specifically in *psb28* but not in *lpa2* or WT (Figs. 6B and 7; Supplementary Fig. S14). These are PsbN, HCF136, HCF244, OHP2, TEF5, and LHL4. All 6 comigrated with D1, D2, and PsbE in Band 18, where some PSII monomers and RC47 were found as well. TEF5, LHL4, and PsbN also comigrated with RCII in Band 22 and TEF5, LHL4, HCF136, and HCF244 accumulated in Bands 28 to 30 with D1 and PsbE (Fig. 7). Most likely, the complex in Band 18 corresponds to the RCII-like complex (Fig. 12), which, in land plants was found to contain RCII (D1, D2, PsbI, E, F), tightly bound HCF244, OHP1 and OHP2, and more loosely bound HCF173, HCF136, RBD1, APE1, and LOW QUANTUM YIELD OF PHOTOSYNTHESIS 1 (LQY1) (Li et al. 2018; Myouga et al. 2018; Maeda et al. 2022). This RCII-like complex corresponds to RCII* in *Synechocystis*, which contains RCII, Ycf48 (HCF136), Ycf39 (HCF244), high-light-inducible proteins HliC/D, RubA (RBD1), PsbN, CyanoP (PPL1), and Slr0575 (APE1) (Knoppova et al. 2014, 2022).

Of the components of this RCII-like complex found in the Chlamydomonas *psb28* mutant (Figs. 7 and 12), HCF244 and OHP2 form the OHC complex together with OHP1 in land plants and green algae, where the absence of any subunit results in severe defects in PSII assembly (Link et al. 2012; Beck et al. 2017; Hey and Grimm 2018; Li et al. 2018; Myouga et al. 2018; Maeda et al. 2022; Wang et al. 2023). The OHC complex corresponds to Ycf39 and HliC/D in *Synechocystis* where these proteins were proposed to play roles in the quenching of excess excitation energy to protect early PSII assembly intermediates and in the delivery of Chl to D1 (Knoppova et al. 2014, 2022; Staleva et al. 2015).

Ycf48 (HCF136) binds at lumenal regions of D1 already in the D1_mod_ and facilitates RCII formation. It prevents premature binding of manganese and other ions and might coordinate the packing of newly synthesized transmembrane helices of D1 with the insertion of Chl and other cofactors and protect D1 from proteolytic attack (Plucken et al. 2002; Komenda et al. 2008; Zhao et al. 2023). PsbN plays an important role in PSII assembly in plants, but its precise role is not yet clear (Torabi et al. 2014; Komenda et al. 2024).

TEF5 and LHL4 have not been detected in the RCII-like complex in plants and algae before (Hey and Grimm 2018; Li et al. 2018; Myouga et al. 2018; Maeda et al. 2022; Wang et al. 2023), possibly because in those studies, the RCII-like complex was isolated in WT backgrounds where it might form too transiently. TEF5 and LHL4 have no orthologs in cyanobacteria, explaining their absence in RCII*. As possible additional components of the RCII-like complex, LHL4 and TEF5 are discussed in more detail below.

### LHL4 is an LHC-like protein sharing some similarity with HliA-D

LHL4 has no assigned role in PSII assembly. LHL4 is an LHC-like protein harboring 3 transmembrane domains of which the region around the first transmembrane helix shares high sequence similarity with the same region in PSBS and with cyanobacterial HliA-D (Supplementary Fig. S22) (Dannay et al. 2025). LHL4 is uniquely found in green microalgae, and in Chlamydomonas, the *LHL4* gene is induced upon UV-B and high light treatment (Teramoto et al. 2004, 2006; Dannay et al. 2025). LHL4 was found to interact with PSII monomers via CP43 and CP47 with a role in protecting PSII from photodamage. LHL4 is barely expressed under low light conditions and interacts with PSII only in the presence of UV-B light (Dannay et al. 2025). We were only able to detect LHL4 in thylakoid membranes of the *psb28* mutant, but not in membranes of the WT or the *lpa2* mutant (Figs. 6B and 7; Supplementary Fig. S14) (Spaniol et al. 2022). Hence, LHL4 present in low light conditions appears to specifically attach to accumulating early PSII assembly intermediates in *psb28*, such as the RCII-like complex, RCII, and D1_mod_ that did not accumulate in the *lpa2* mutant or WT. Alternatively, PSII assembly intermediates accumulating in the *psb28* mutant might trigger the upregulation of LHL4 (e.g. via enhanced production of reactive oxygen species) that then attaches to the present PSII assemblies. Most likely, LHL4 protects these early PSII assembly intermediates from photodamage or plays a role in binding Chl released from degrading early PSII assemblies, as was proposed for HliC/D in cyanobacteria (Knoppova et al. 2014, 2022; Staleva et al. 2015). Since PSII appears to accumulate normally in the *lhl4* mutant (Dannay et al. 2025), it is unlikely that LHL4 plays a direct role during PSII assembly.

### TEF5 is involved in PSII assembly in Chlamydomonas, possibly by aiding in RCII maturation for the correct incorporation of the CP47_mod_

Common features of TEF5 and PSB33/LIL8 are the similarity of the predicted structures of their Rieske-like domains (Fig. 8B), their attachment to the thylakoid membrane via transmembrane helices, and their ability to interact with PSII and PSI (Fristedt et al. 2015, 2017; Kato et al. 2017) (Fig. 11, F and G). Moreover, Chlamydomonas *tef5* and Arabidopsis *psb33/lil8* mutants share a PSII phenotype with reduced accumulation of PSII core subunits, which is constitutive in Chlamydomonas but emerges only under certain environmental conditions in Arabidopsis (Fristedt et al. 2015, 2017; Cruz et al. 2016; Nilsson et al. 2020) (Fig. 8, D, E, and G). Arabidopsis *psb33/lil8* showed swollen thylakoids in blue light potentially corresponding to the increased frequency of interrupted thylakoid stacking in the Chlamydomonas *tef5* mutant grown in low light (Fig. 9B) (Nilsson et al. 2020). In the Chlamydomonas *tef5* mutant, in low light, PSII subunits accumulated to 20% to 40% of WT levels (Fig. 8, D and E), with monomers, dimers, and supercomplexes accumulating at a lower level than WT and RC47 being undetectable, whereas RCII and CP43_mod_ overaccumulated (Fig. 11, A and B). The PSII phenotype was attenuated when the *tef5* mutant was grown in the dark, with PSII monomers, dimers, and supercomplexes accumulating at higher levels and RCII at a lower level than in the light-grown mutant, and RC47 became detectable (Fig. 11B).

We speculate that TEF5, possibly together with LHL4, associates already with the D1_mod_ and guides its assembly into RCII and the RCII-like complex, accounting for their comigration with these early PSII assemblies in Bands 28 to 30, 22, and 18 (Figs. 7 and 12). However, we cannot exclude that the smaller assemblies are breakdown products of the RCII-like complex. Here, TEF5 (together with LHL4 or PSI?) could play roles in cofactor delivery, photoprotection, or in introducing a conformational change in RCII that is required for the stable incorporation of CP47_mod_ for RC47 formation. RCII accumulating in the absence of TEF5 (Fig. 11, A and B) could represent an immature or damaged form. The observation that less RCII, more RC47, and more functional higher PSII assembly states accumulate in the dark-grown *tef5* mutant (Fig. 11, B and C) points to a role of TEF5 in photoprotection during PSII assembly. However, since the WT phenotype is not entirely restored in the dark, photoprotection cannot be the only function of TEF5. The strong accumulation of RC47 in dark- versus light-grown WT cells points to a slower PSII monomer assembly pace in the dark, which might facilitate correct CP47_mod_ incorporation into RCII even in the absence of TEF5 and could also explain the attenuated PSII phenotype in the dark-grown *tef5* mutant (Fig. 11B). In *Synechocystis*, an alternative pathway for photoprotection and Chl delivery during RCII assembly by Ycf39/Hlips was proposed to occur via PSI (Knoppova et al. 2014, 2022; Zhao et al. 2023). Here, it is interesting that TEF5 can bind PSI (Fig. 11G), possibly suggesting that TEF5 with LHL4 and/or PSI may represent such an alternative pathway to HCF244/OHPs respectively Ycf39/Hlips in RCII maturation. It is tempting to speculate that the frequently occurring suppressor mutations observed for Chlamydomonas *ohp2* mutants (Wang et al. 2023) somehow activate such an alternative pathway around TEF5.

We suspect that in the absence of TEF5/PSB33, a fraction of PSII is malformed, with defects such as a damaged Q_B_ site, as observed by Cruz et al. (2016). An even more effective protein quality control system in Chlamydomonas than in Arabidopsis could explain why such malformed PSII assemblies are cleared in Chlamydomonas, whereas they can persist in Arabidopsis. This could be similar in *psb28* mutants. The mild, pale-green phenotype of a rice *psb28* knockout line suggests that some functional PSII can be assembled in the absence of PSB28 (Jung et al. 2008), whereas in the Chlamydomonas *psb28* mutant, hardly any functional PSII is made.

AlphaFold3 failed to predict an interaction of correctly oriented TEF5/PSB33 with RCII. This could be explained by the binding of TEF5 to RCII via one of the other RCII-associated proteins, which we did not test (Fig. 12). However, AlphaFold3 predicted TEF5/PSB33 to interact strongly with CP47/PsbH in PSII (Supplementary Figs. S19 and S20), but the mutation of amino acids constituting the contact sites between TEF5 and CP47/PsbH did not abolish TEF5's function in PSII assembly (Supplementary Fig. S21). Possibly, the AlphaFold3 model is incorrect. Alternatively, TEF5 might interact with CP47/PsbH in PSII to exert another function in addition to that in PSII assembly, e.g. in balancing the excitation energy distribution between PSI and PSII (Kato et al. 2017).

The clearly reduced synthesis rates of CP47 and PsbH in the *psb28* and *tef5* mutants (Figs. 3 and 10) suggests that a similar CES-like negative feedback control is at work in both. As was proposed above, this might be directly or indirectly triggered by RCII and CP43_mod_ accumulating in both, *psb28* and *tef5*. RCII and CP43_mod_ did not accumulate in the Chlamydomonas *lpa2* mutant, where no reduced translation rates were observed for any PSII core subunit (Spaniol et al. 2022).

## Materials and methods

### Strains and culture conditions

*C. reinhardtii* WT CC-4533 and mutant strains LMJ.RY0402.193950 (*psb28*) and LMJ.RY0402.242855 (*tef5*) from the CLiP (Li et al. 2016) were obtained from the Chlamydomonas Resource Center. The *psb28* mutant was transformed with plasmid pMBS687 to generate complemented lines *psb28*-c2 and *psb28*-c6. The *tef5* mutant was transformed with plasmids pMBS703 and pMBS756 to generate complemented lines *tef5*-c15 and *tef5*-HA, respectively, and with plasmids pMBS1356-1371 to generate complemented lines with point mutations in TEF5. Transformation was done via agitation with glass beads (*psb28* mutant) (Kindle 1990) and electroporation (*tef5* mutant) (Shimogawara et al. 1998). Unless indicated otherwise, cultures were grown mixotrophically in TAP medium (Kropat et al. 2011) on a rotatory shaker at 25 °C and ∼30 *µ*mol photons m^−2^ s^−1^ provided by MASTER LEDtube HF 1,200 mm UO 16W830 T8 and 16W840 T8 (Philips). For high light exposure, cells were grown to a density of 2 ∼ 10^6^ cells mL^−1^, transferred to an open 1-L beaker, placed on an orbital shaker, and exposed to 1,200 to 1,800 *µ*mol photons m^−2^ s^−1^ provided by CF Grow (CXB3590-X4). Cell densities were determined using a Z2 Coulter Counter (Beckman Coulter). For spot tests, cells were grown to a density of 3 to 5 × 10^6^ cells mL^−1^ and diluted in TAP medium such that 10 *µ*L contained 10^4^, 10^3^, or 10^2^ cells. Ten microliters of each dilution were spotted onto agar plates containing TAP medium or HSM medium and incubated in low light (30 *µ*mol photons m^−2^ s^−1^) for 72 h, high light (600 *µ*mol photons m^−2^ s^−1^) for 72 h, or in the dark for 96 h. HSM was prepared according to Sueoka (1960), but using the trace solutions from Kropat et al. (2011).

### Cloning of constructs for complementing the *psb28 and tef5* mutants

The Chlamydomonas *PSB28* coding sequence, including both introns, was amplified by PCR from Chlamydomonas genomic DNA in 2 fragments of 715 and 204 bp to remove an internal BsaI site using primers PSB28-1/2 and PSB28-3/4, respectively (Supplementary Table S2). The PCR products were cloned into the recipient plasmid pAGM1287 (Weber et al. 2011) by restriction with BbsI and ligation with T4-DNA ligase, resulting in the Level 0 construct pMBS685 (Level 0 constructs contain gene parts). The *Synechocystis psb28-1* coding sequence, interrupted by the first *RBCS2* intron, was synthesized by BioCat (Heidelberg) with optimal Chlamydomonas codon usage and cloned into pAGM1287, yielding Level 0 construct pMBS695. The Chlamydomonas *TEF5* coding sequence, interrupted by the first 2 *RBCS2* introns, was synthesized by BioCat (Heidelberg) and cloned into pAGM1287, giving Level 0 construct pMBS701. The B3-B4 Level 0 parts with the coding sequences were then complemented with Level 0 parts (pCM) from the Chlamydomonas MoClo toolkit (Crozet et al. 2018; Niemeyer et al. 2021) to fill the respective positions in Level 1 modules (containing transcription units) as follows: A1-B2, pCM0-020 (*HSP70A-RBCS2* promoter + 5′ UTR); B5, pCM0-100 (3xHA) or pCM0-101 (MultiStop); and B6, pCM0-119 (*RPL23* 3′UTR). The Level 0 parts and destination vector pICH47742 (Weber et al. 2011) were directionally assembled into Level 1 modules pMBS686 (PSB28-3xHA), pMBS696 (SynPsb28-1-3xHA), pMBS702 (TEF5-MultiStop), and pMBS755 (TEF5-3xHA) with BsaI and T4-DNA ligase. Level 1 modules were then combined with pCM1-01 (Level 1 module with the *aadA* gene conferring resistance to spectinomycin), with plasmid pICH41744 containing the proper end-linker, and with destination vector pAGM4673 (Weber et al. 2011), digested with BbsI, and ligated to yield Level 2 devices pMBS687 (PSB28-3xHA), pMBS697 (SynPsb28-3xHA), pMBS703 (TEF5-MultiStop), and pMBS756 (TEF5-3xHA). Level 2 devices contain multiple transcription units.

To introduce point mutations into TEF5, site-directed mutagenesis was performed via PCR (Q5 Site-Directed Mutagenesis Kit, NEB) using pMBS703 as template and the primers listed in Supplementary Table S2. The PCR products were combined and/or circularized by restriction with BbsI and ligation with T4-DNA ligase resulting in Level 2 constructs pMBS1356 (W282A), pMBS1357 (Y223A), pMBS1358 (F216K), pMBS1359 (F170A), pMBS1360 (W282D), pMBS1361 (F170A/F216K), pMBS1363 (Y223A/W282A), and pMBS1364 (F170A/F216 K/Y223A). Additionally, the TEF5 coding sequence harboring the mutations for F170A/F216K/Y223A/W282A was synthesized by TWIST and cloned into pAGM1287 by restriction with BsaI and ligation with T4-DNA ligase, yielding Level 0 construct pMBS1368, and subsequently assembled together with the Level 0 parts listed above (*HSP70A-RBCS2* promoter + 5′ UTR, CDJ1 chloroplast transit peptide, MultiStop, and *RPL23* 3′UTR) into Level 2 destination vector pMBS807 (Niemeyer and Schroda 2022), yielding pMBS1371. Correct cloning was verified by Sanger sequencing. All MoClo constructs employed and generated are listed in Supplementary Table S3.

### Production of recombinant PSB28 in *Escherichia coli*

The PSB28 coding region lacking the predicted chloroplast transit peptide (Fig. 1A) was PCR-amplified from cDNA using oligonucleotides PSB28-Bam and PSB28-Hind (Supplementary Table S2). The resulting 438-bp PCR product was digested with BamHI and HindIII and cloned into the pETDuet vector (Novagen) (pMS1079), introducing an N-terminal 6xHis tag. Recombinant PSB28 was produced in *Escherichia coli* ER2566 and purified by Ni-NTA affinity chromatography. Recombinant CGE1 was produced and purified as described previously (Willmund et al. 2007).

### Genotyping

3 × 10^7^ Chlamydomonas cells were centrifuged at 3,500 × *g* for 5 min. The pellet was resuspended in 250-*μ*L water, followed by the addition of 250-*μ*L 100 mm Tris-HCl pH 8, 10 mm EDTA, 4% SDS, and incubation with proteinase K for 1 h at 55 °C. Subsequently, 80-*μ*L 5 m KCl and 70-*μ*L CTAB/NaCl (10%/4%) were added, followed by agitation at 65 °C for 10 min. DNA was extracted first with phenol/chloroform/isoamyl alcohol (25:24:1) and then with chloroform/isoamyl alcohol (24:1). DNA was then precipitated with isopropanol and washed with 70% EtOH. The dried DNA pellet was dissolved in TE buffer (10 mm Tris-HCl pH 8, 1 mm EDTA) containing RNase. For PCR, genomic DNA, KAPA GC reaction buffer and KAPA Hifi HotStart Polymerase (Roche), 1 m betaine, 0.2 mm deoxynucleotide triphosphates, and 0.3 mm of the respective primers were mixed, incubated at 95 °C for 3 min and subjected to 35 cycles of 98 °C for 20 s, 63 °C for 20 s, and 72 °C for 90 s, followed by 75 s at 72 °C.

### RT-qPCR

RNA extraction and RT-qPCR analysis were done as described previously for the *lpa2* mutant (Spaniol et al. 2022) using the primers for *TEF5* and *CBLP2* as housekeeping control listed in Supplementary Table S2.

### SDS–PAGE and immunoblot analyses

Cells were harvested by centrifugation and either frozen as pellets at −20 °C or directly processed. Frozen cell pellets were resuspended in sample buffer containing 62 mm Tris-HCl, pH 6.8, 2% (*w*/*v*) SDS, and 10% (*v*/*v*) glycerol, boiled for 1 min at 95 °C, cooled on ice for 2 min, and centrifuged at 18,500 × *g* and 25 °C. Samples were diluted with sample buffer containing 50 mm DTT and 0.01% bromophenol blue to 1 *μ*g protein *μ*L^−1^. Directly processed cells were resuspended in DTT-SDS-sucrose buffer (0.1 m Na_2_CO_3,_ 0.1 m DTT, 5% [*w*/*v*] SDS, 30% [*w*/*v*] sucrose), boiled for 2 min at 95 °C, and centrifuged at 12,000 × *g* and 25 °C for 5 min. Samples were subjected to SDS–PAGE and semidry western blotting based on 1 *µ*g protein *µ*L^−1^ or 1 to 2 *µ*g Chl. Antisera used were against D1 (Agrisera AS05 084), D2 (Agrisera AS06 146), CP43 (Agrisera AS11 1787), CP47 (Agrisera AS04 038), LHCBM9 (M. Schroda, unpublished data), PsaA (Agrisera AS06 172), PSAD (Agrisera 1136 AS09 461), PSAN (M. Schroda, unpublished data), Cyt *f* (Pierre and Popot 1993), CGE1 (Schroda et al. 2001), CF1β (Lemaire and Wollman 1989), RPL1 (Ries et al. 2017), and the HA-tag (Sigma-Aldrich H3663). Peptide antibodies against PSB28 and TEF5 were produced by Pineda (Berlin). Anti-rabbit-HRP (Sigma-Aldrich) was used as secondary antibody. Densitometric band quantifications after immunodetections were done with the FUSIONCapt software or the plug-in FiJi (Schindelin et al. 2012) of ImageJ. To identify differences in protein abundances, 2-tailed unpaired *t*-tests were performed, followed by Bonferroni–Holm correction for multiple testing when applicable (Supplementary Data Set S5).

### Pulse-chase labeling

Cells in the exponential growth phase (2 × 10^6^ cells mL^−1^) from a 100-mL culture were harvested by centrifugation, washed with minimum medium, and resuspended in 1/20th volume of minimum medium. Cells were allowed to recover and to deplete their intracellular carbon pool for 1.5 h under dim light (20 *µ*E m^−2^ s^−1^) and strong aeration at 25 °C; 10 *µ*m cycloheximide and 10 *µ*Ci mL^−1^ Na-^14^C acetate (PerkinElmer: 56.6 mCi mM^−1^) were then added to the culture for the 7-min pulse. Cell samples, collected immediately after centrifugation at 4 °C, were resuspended in ice-cold 0.1 m dithiothreitol and 0.1 m Na_2_CO_3_, frozen in liquid nitrogen, and kept at −80 °C until analysis. For chase experiments, pulse-labeled cells were diluted in 35 mL of TAP medium containing 50 mm nonradioactive acetate and 250 *μ*g mL^−1^ CAP at 25 °C and further incubated in this medium for 20 and 60 min. Cells were then collected by centrifugation at 4 °C and treated as above.

### BN–PAGE

BN–PAGE was performed with minor modifications according to Jarvi et al. (2011). For the analysis of whole-cell proteins, 2 × 10^8^ cells (or 60-*µ*g isolated thylakoids; see below) were centrifuged for 5 min at 4,400 × *g*, 4 °C, and resuspended in 750 *μ*L of TMK buffer (10 mm Tris-HCl pH 6.8, 10 mm MgCl_2_, 20 mm KCl). After a further centrifugation step for 2 min at 2,150 × *g*, 4 °C, the pellet was resuspended in 350-*µ*L ACA buffer (750 mm ɛ-aminocaproic acid, 50 mm bis-Tris/HCl pH 7.0, 0.5 mm EDTA), mixed with 4 *μ*L of 25-fold protease inhibitor (Roche), and frozen at 80 °C. The sample was then thawed on ice and sonicated for 30 s (output: 25%, cycle: 70%), followed by a 5-min centrifugation at 300 × *g* and 4 °C. The protein concentration of the supernatant was determined according to Bradford (1976), and the sample was diluted with ACA buffer to 1.2 *µ*g protein *µ*L^−1^. For solubilization, 225 *µ*L of the sample were mixed with 25 *µ*L 10% β-DDM and incubated on ice for 20 min. After a centrifugation for 10 min at 18,500 × *g* and 4 °C, 15-*μ*L loading buffer (250 mm ɛ-aminocaproic acid, 75% glycerol, 5% Coomassie Brilliant Blue 250 G) was added to the supernatant, and samples were centrifuged several times at 18,500 × *g* and 4 °C until insoluble material was no longer present. Samples were then loaded on 4% to 15% BN acrylamide gels. Gels were either stained with Coomassie Brilliant Blue, or the protein complexes were transferred to PVDF membranes. For the latter, the gel was incubated for 30 min in T2 buffer (25 mm Tris-HCl pH 10.4, 20% isopropanol) containing 0.1% SDS and then for a further 15 min in T2 buffer without SDS. The PVDF membrane (0.45 *μ*m) was soaked in methanol for 15 s and washed twice for 5 min with water. The membrane was then incubated in T2 buffer for 10 min. Proteins were transferred onto the membrane by semidry blotting using T1 buffer (25 mm Tris-HCl pH 9.8, 40 mm ɛ-aminocaproic acid, 20% isopropanol) containing 0.01% SDS.

For CP, WT and *psb28* mutant were grown in 3 independent cultures and thylakoids were isolated from harvested cells according to Chua and Bennoun (1975) with minor modifications. Briefly, 2 × 10^9^ cells were pelleted and washed with 25 mm HEPES-KOH, pH 7.5, 5 mm MgCl_2_, and 0.3 m sucrose, before resuspending in the same buffer supplemented with protease inhibitor (Roche). Cells were then lysed using a BioNebulizer (Glas-Col) with an operating N_2_ pressure of 1.5 bar. After centrifugation at 3,500 × *g* for 10 min, the pellet was washed with 5 mm HEPES-KOH, pH 7.5, 1 mm EDTA, and 0.3 m sucrose before resuspending in 5 mm HEPES-KOH, pH 7.5, 1 mm EDTA, and 1.8 m sucrose. After placing 1.3 and 0.5 m sucrose layers in the same buffer on top and centrifugation at 100,000 × *g* for 1 h, intact thylakoids, floating between the 1.3 m and 1.8 m layers, were collected and diluted with 5 mm HEPES-KOH, pH 7.5, and 1 mm EDTA.

### In-gel digestion and MS for CP

Coomassie-stained BN–PAGE gel pieces were destained by repeated cycles of washing with 40 mm NH_4_HCO_3_ for 5 min and incubating in 70% acetonitrile for 15 min, until they were colorless. They were then dehydrated completely by adding 100% acetonitrile for 5 min and dried under vacuum. Samples were then digested by covering the gel pieces in 10-ng/*µ*L trypsin in 40 mm NH_4_HCO_3_ and incubating them overnight at 37 °C, before first, hydrophilic peptides were extracted with 10% acetonitrile and 2% formic acid for 20 min, and afterward, all other tryptic peptides were extracted with 60% acetonitrile and 1% formic acid. Samples were combined and desalted according to Rappsilber et al. (2007). MS was performed as described previously (Hammel et al. 2018; Spaniol et al. 2022).

The analysis of MS runs was performed using MaxQuant version 1.6.0.16 (Cox and Mann 2008). Library generation for peptide spectrum matching was based on the *C. reinhardtii* genome release 5.5 (Merchant et al. 2007) including chloroplast and mitochondrial proteins. Oxidation of methionine and acetylation of the N-terminus were considered as peptide modifications. Maximal missed cleavages were set to 3 and peptide length to 6 amino acids, the maximal mass to 6,000 Da. Thresholds for peptide spectrum matching and protein identification were set by a false discovery rate of 0.01. The MS proteomic data have been deposited to the ProteomeXchange Consortium via the PRIDE (Perez-Riverol et al. 2019) partner repository with the data set identifier PXD023478. Total protein group intensities varied between samples. For sample normalization, the total ion intensity sum (TIS) of every protein and gel slice was calculated for each of the 6 samples (3× WT and 3× mutant). Sample normalization was performed by aligning protein group intensities of ATP synthase subunits ATPC, atpI, atpE, atpF, atpB, atpA, ATPD, and ATPG using the median of ratios method (Love et al. 2014). This resulted in a single correction factor for each sample. Subsequently, every intensity value was divided by its sample-specific correction factor, to equalize all TISs. For further analysis, proteins identified by nonproteotypic peptides were discarded. Protein identifiers were annotated with MapMan ontology terms, gene ontology terms, and proposed subcellular localization (https://doi.org/10.5281/zenodo.6340413). A Welch test was performed for each protein by considering the sums of all 36 normalized slice intensities for each sample and testing 3 WT sums against 3 mutant sums. The distance of the average migration profiles for every protein was calculated as the Euclidean distance between WT and mutant. To adjust for amplitude-introduced bias, each distance was divided by the maximal average intensity of WT or mutant, respectively. Data normalization and analysis were performed using FSharp.Stats (https://doi.org/10.5281/zenodo.6337056). The migration profiles were visualized using Plotly.NET (Schneider et al. 2022).

### Immunoprecipitation

Two hundred milliliters of culture was grown in HAP medium (TAP in which Tris was replaced by 20 mm HEPES-KOH pH 7.0) and supplied for 10 min with formaldehyde (0.37% final concentration) for in vivo cross-linking; 100 mm Tris-HCl pH 8.0 was added to the culture for quenching before cells were collected by centrifugation for 5 min at 2,500 × *g* and 4 °C. The cell pellet was resuspended in 1.5-mL TE buffer and frozen at −20 °C. After thawing at 23 °C, 20-*μ*L PMSF was added, and samples were frozen in liquid nitrogen. After 2 more cycles of thawing and freezing, 50 *μ*L were taken to determine the protein concentration, and samples were centrifuged for 30 min at 18,000 × *g* and 4 °C; 40 mm Tris-HCl pH 8, 150 mm NaCl, 1 mm MgCl_2_, 10 mm KCl, and 0.1% α-DDM were then added to the supernatant. The pellet was resuspended in TNMK buffer (50 mm Tris-HCl pH 8, 150 mm NaCl, 1 mm MgCl_2_, 10 mm KCl) containing 1% α-DDM. Samples were then mildly sonicated and centrifuged at 14,000 × *g* and 4 °C for 10 min after a 5-min incubation on ice. The supernatants were added to 20-*μ*L HA-coupled magnetic beads (Pierce), and the samples were incubated for 1.5 h at 4 °C. After 3 washing steps with TNMK buffer containing 0.05% Tween and 3 washing steps with TNMK buffer, 100 *μ*L of sample buffer (90 mm Tris-HCl, 20% glycerol, 2% SDS) were added, and the samples were boiled for 1 min. The eluate was removed from the magnetic beads, mixed with 50 mm DTT, and boiled for an additional 10 min. The eluates were then analyzed by SDS–PAGE and immunodetection or by MS.

### Chl fluorescence measurements

Chl fluorescence was measured using a pulse amplitude-modulated Mini-PAM fluorometer (Mini-PAM, H. Walz, Effeltrich, Germany) essentially according to the manufacturer's protocol after 3 min of dark adaptation (0.6-s saturating pulse of 5,500 *μ*mol photons m^−2^ s^−1^, gain = 1). To identify differences between the analyzed strains, 2-tailed unpaired *t*-tests were performed, followed by Bonferroni–Holm correction for multiple testing when applicable.

### Chl precursors

The tetrapyrrole biosynthesis intermediates and end-products were analyzed by HPLC, essentially as described previously (Brzezowski et al. 2014), on cultures grown in dark or in low light (30 *μ*mol photons m^−2^ s^−1^). In short, samples containing 1.2 × 10^8^ cells were centrifuged at 3,000 × *g* for 5 min at 4 °C, and the pellets were snap-frozen in liquid N_2_. Protoporphyrin IX, Mg-protoporphyrin IX, Mg-protoporphyrin IX monomethylester, protochlorophyllide, chlorophyllide, Chl a and b, and pheophorbide were extracted in 500-*µ*L cold (−20 °C) acetone/0.1 m NH_4_OH (9/1, *v*/*v*) with sonication, followed by a 3-step cycle of resuspension and centrifugation using the same solution. Heme extraction was performed on the remaining pellet using 100-*µ*L acetone/HCl/DMSO (10/0.5/2, *v*/*v*/*v*) in the same 3-step protocol. HPLC analyses were performed essentially as described in Czarnecki et al. (2011). Values were normalized to pmol/10^6^ cells.

### P700 decay kinetics

Measurements of P700+ reduction kinetics were conducted using a Dual-PAM-100 instrument from Heinz Walz (Effeltrich, Germany), with Chl concentrations set to 5 *µ*g/mL. To fully oxidize P700, a 50-ms multiple-turnover light pulse at an intensity of 10,000 *µ*mol photons m^−2^ s^−1^ was administered following a 2-min dark incubation period. For WT, *psb28*, and complemented lines, the reduction kinetics of P700+ were monitored without any additions and in the presence of 100 *µ*m DCMU. The average results from 4 separate experiments were fitted with single exponential functions and analyzed as described previously (Bernát et al. 2009). The presented values were calculated using 1-way ANOVA analysis with the GraphPad Prism software.

### Light, fluorescence, and TEM

Light microscopy was carried out with an Olympus BX53 microscope using a UPlanXApo 100× lense, and Chl autofluorescence WAS recorded with a Zeiss LSM 880 Axio Observer confocal microscope using a 63× oil objective. Fluorescent signals (excitation 633 nm, detection 647 to 721 nm) were acquired using the Zeiss software ZEN 2.3. Image processing was either conducted with ZEN 2.3 or ImageJ (https://imagej.net/ij/). For TEM, cells were harvested and initially fixed with 2.5% glutaraldehyde. High-pressure freezing was performed using an EM HPM100 (Leica Microsystems, Wetzlar, Germany), followed by freeze substitution with 0.2% osmium tetroxide, 0.1% uranyl acetate, and 9% water in pure acetone in an EM AFS2 (Leica Microsystems) for a duration of 42 h, as described by Peschke et al. (2013). The samples were then embedded in Epon 812 resin and polymerized at 63 °C for 48 h. Ultrathin sections (50-nm thickness) were prepared with a 35° diamond knife (DiATOME, Nidau, Switzerland) on an Ultracut E ultramicrotome (Leica Microsystems). These sections were mounted onto collodion-coated, 75-mesh copper grids (Science Services GmbH, Munich, Germany), poststained with 80 mm lead citrate in NaOH at pH 13, and analyzed using an EM 912 transmission electron microscope (Zeiss, Oberkochen, Germany) equipped with an integrated OMEGA energy filter operated in 0-loss mode at 80 kV. Image acquisition was performed with a 2k × 2k slow-scan CCD camera (Tröndle Restlichtverstärkersysteme, Moorenweis, Germany).

### Immunofluorescence microscopy

Formaldehyde was added to a final concentration of 4% to 1-mL Chlamydomonas cells grown to log phase, followed by an incubation at 4 °C for 1 h. Ten microliters of 0.1% poly-L-lysine were applied to a microscopy slide, and 40 *μ*L of fixed cells were added. The slide was then placed into ice-cold methanol for 6 min. Subsequently, the slide was washed 5 times with phosphate-buffered saline (PBS). For permeabilization, cells were incubated in PBS containing 2% Triton at 25 °C. After 5 more washing steps with PBS containing 5 mm MgCl_2_, the slide was incubated in PBS containing 1% BSA, and the primary antibody was added (rabbit anti-D1, Agrisera AS05 084, 1:10,000; mouse anti-HA, Pineda, 1:3,000), followed by an incubation overnight. After 5 washes with PBS containing 1% BSA, the secondary antibody (fluorescine-isothiocyanate-labeled goat anti-rabbit [Sigma-Aldrich]; Alexa Fluor 488 goat anti-mouse [Thermo Fisher Scientific], 1:500) was added followed by an incubation for 1.5 h. Five last washes with PBS followed before microscopy images were taken with the Zeiss LSM880 AxioObserver confocal laser scanning microscope equipped with a Zeiss C-Apochromat 40×/1,2 W AutoCorr M27 water-immersion objective. Fluorescent signals of FITC (excitation/emission 488 nm/493 to 553 nm) and Alexa Fluor 546 (excitation/emission 543 nm/553 to 669 nm) were processed using the Zeiss software ZEN 2.3 or Fiji software. For double labeling, images were acquired using sequential scan mode to avoid channel crosstalk.

### Structural modeling

The complexes formed by TEF5 and PSB33 with the respective PSII cores were predicted by AlphaFold2 (Jumper et al. 2021) and AlphaFold3 (Abramson et al. 2024). We used a local AlphaFold2 installation through ColabFold (Mirdita et al. 2022) to predict 5 different complexes. Models were ranked based on the ColabFold scoring tools IDDT (Mariani et al. 2013) and TM (Zhang and Skolnick 2004). The predicted structures were subsequently minimized utilizing the AMBER force field optimization (Weiner et al. 1984). Analogously, the complexes were predicted using the AlphaFold3web server. As AlphaFold2 and AlphaFold3 gave comparable results, only the best-ranked AlphaFold3 structures were used for further interaction evaluation. Structural similarities were evaluated using the Cα RMSD and TM score, calculated with TM-align (Zhang and Skolnick 2005). The contact network of the assembly factors TEF5 and PSB33 with RCII and the PSII monomer were analyzed with MAXIMOBY/MOBY (CHEOPS, Germany) and PyContact (Scheurer et al. 2018).

### Sequence alignments, motif search, and pairwise structure comparisons

Putative chloroplast transit peptides of PSB28 and TEF5 homologs were predicted with TargetP (Almagro Armenteros et al. 2019) and putative transmembrane helices with DeepTMHMM (Hallgren et al., 2022). Sequence motifs were searched by InterProScan (Jones et al. 2014). Sequence alignments were done with CLUSTALW (https://www.genome.jp/tools-bin/clustalw) and displayed with GeneDoc.

### Accession numbers

Sequence data from this article can be found in Phytozome under accession numbers Cre10.g440450 (PSB28) and Cre09.g411200 (TEF5). See also Supplementary Table S1.

## Supplementary Material

koaf055_Supplementary_Data

## Data Availability

The data underlying this article are available in the article and in its online Supplementary material.

## Supplementary data

The following materials are available in the online version of this article.

**Supplementary Figure S1.** Analysis of the CIB1 integration site in the *PSB28* gene by PCR and testing of the PSB28 peptide antibody.

**Supplementary Figure S2.** Screening for complemented *psb28* transformants.

**Supplementary Figure S3.** Analysis of Chl precursor contents in *psb28* mutant and WT.

**Supplementary Figure S4.** Collection of light microscopy and fluorescence microscopy images of WT, *tef5*, and *psb28*.

**Supplementary Figure S5.** Collection of electron micrographs from thylakoids of WT, *tef5*, and *psb28*.

**Supplementary Figure S6.** Analysis of PSII complex assembly, subunit accumulation, and functionality in dark-grown cells.

**Supplementary Figure S7.** Analysis of oligomerization capacity of recombinant PSB28 and quantification of cellular PSB28 abundance.

**Supplementary Figure S8.** Monitoring kinetics of PSII repair after photoinhibition in the *psb28* mutant.

**Supplementary Figure S9.** Monitoring kinetics of PSII resynthesis in the *psb28* mutant after sulfur starvation.

**Supplementary Figure S10.** Construct for the expression of *Synechocystis* Psb28-1 and analysis of transformants in the *psb28* mutant background.

**Supplementary Figure S11.** BN–PAGE for CP.

**Supplementary Figure S12.** Comparison of BN–PAGE migration profiles of PSII core subunits and of the PSII-associated protein PBA1.

**Supplementary Figure S13.** Comparison of BN–PAGE migration profiles of thylakoid membrane protease FTSH1/2 and kinase STL1.

**Supplementary Figure S14.** Comparison of BN–PAGE migration profiles of PSII core subunits, of known PSII auxiliary factors, and other auxiliary factors.

**Supplementary Figure S15.** Analysis of the CIB1 integration site in the *TEF5* gene by PCR and of TEF5 protein in the *tef5* mutant and in complemented lines.

**Supplementary Figure S16.** Screening for complemented *tef5* transformants.

**Supplementary Figure S17.** Monitoring kinetics of PSII repair after photoinhibition.

**Supplementary Figure S18.** Monitoring kinetics of PSII resynthesis in the *tef5* mutant after sulfur starvation.

**Supplementary Figure S19.** Predicted TEF5/PSB33-PSII core complexes.

**Supplementary Figure S20.** Predicted contact sites of TEF5 with CP47 and PsbH in Chlamydomonas PSII.

**Supplementary Figure S21.** Complementation analysis of the *tef5* mutant with TEF5 carrying amino acid exchanges at residues predicted to mediate its interaction with CP47.

**Supplementary Figure S22.** Alignment of N-terminal regions of green algal LHL4 and PSBS proteins with cyanobacterial HliA-D.

**Supplementary Table S1.** Proteins involved in PSII assembly, repair, or complex dynamics in Arabidopsis or *Synechocystis* that have clear homologs in Chlamydomonas and are present with 3 replicates each for WT and *psb28* mutant in the CP data set.

**Supplementary Table S2.** Primers used for genotyping, cloning, and RT-PCR.

**Supplementary Table S3.** MoClo constructs employed and generated.

**Supplementary Data Set S1.** LC-MS/MS analysis of PSB28 immunoprecipitates.

**Supplementary Data Set S2.** Interactive CP data set.

**Supplementary Data Set S3.** Heat maps of all proteins found in the CP data set in the WT and the *psb28* mutant.

**Supplementary Data Set S4.** LC-MS/MS analysis of TEF5 immunoprecipitates.

**Supplementary Data Set S5.** Compilation of statistical analyses.
