# Supplementary material for: Complexome profiling of the Chlamydomonas psb28 mutant reveals TEF5 as an early PSII assembly factor
Source: Plant Cell. 2025 Mar 17;37(6):koaf055. doi: 10.1093/plcell/koaf055 (PMC12164590; doi:10.1093/plcell/koaf055)
Supplement: koaf055_Supplementary_Data [file koaf055_supplementary_data.zip › Supplementary Data final.pdf]

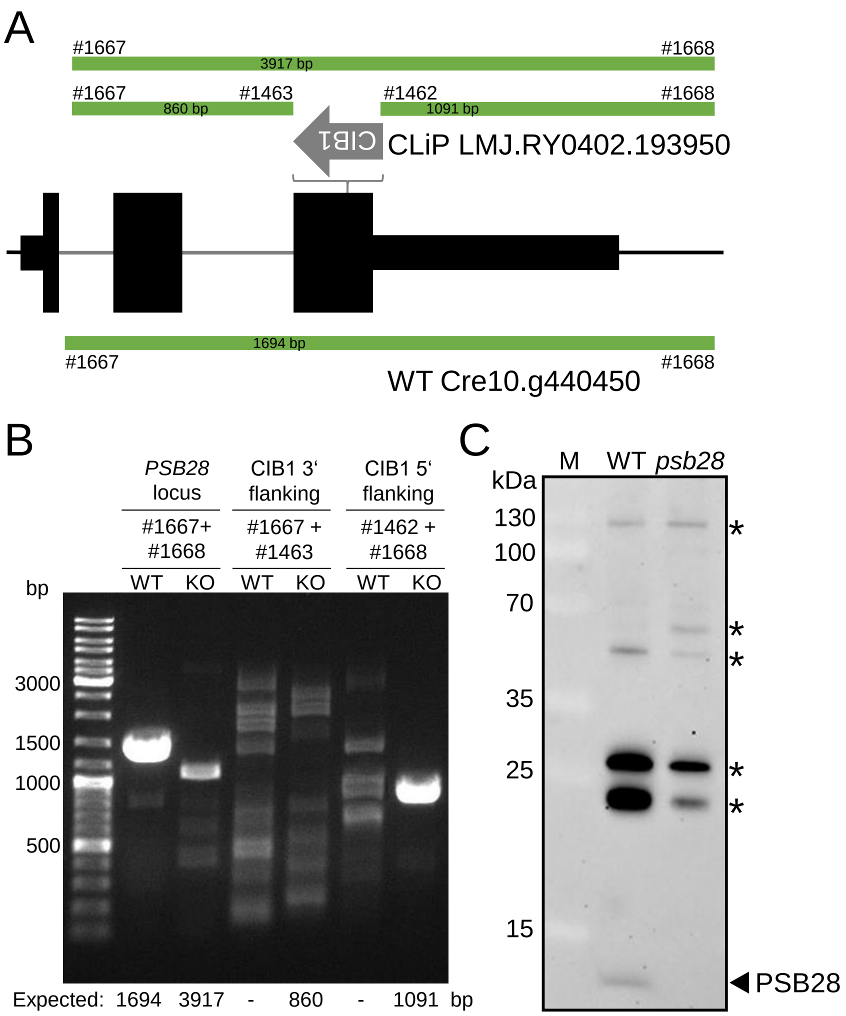


**Supplementary Figure S1. Analysis of the CIB1 integration site in the *PSB28* gene by PCR and testing of the PSB28 peptide antibody.**
**(A)** Gene model of the *PSB28* gene with exons shown as black boxes and introns as thin grey lines. The integration site of the CIB1 cassette (https://www.chlamylibrary.org/showCassette?cassette=CIB1) in the third exon is shown. Green bars indicate the expected PCR products on mutant DNA (CLiP, top) and wild-type DNA (WT, bottom). Numbers in the bars indicate their sizes. Numbers flanking the bars are the primer numbers (Supplementary Table 1).
**(B)** PCR products on genomic DNA from wild type (WT) and the *psb28* mutant from the CLiP collection (KO) were separated on an agarose gel and stained with Gel Red. Expected amplicon sizes are indicated below the gel.
**(C)** Immunoblot analysis to test the PSB28 peptide antibody. 10 µg of whole-cell proteins from wild type (WT) and *psb28* mutant were separated on a 12% SDS-polyacrylamide gel and analyzed by immunoblotting using a 1:500 dilution of the PSB28 antibody. Asterisks indicate cross-reactions, the arrowhead the expected position of the PSB28 protein band (at ~12.6 kDa).
Supports Figure 1.

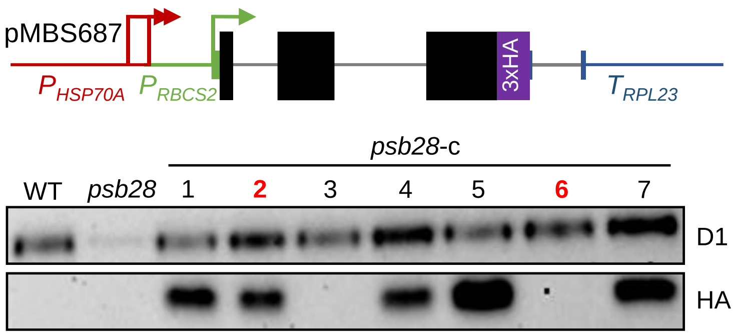


**Supplementary Figure S2. Screening for complemented *psb28* transformants.**
*psb28* mutant cells were transformed with construct pMBS687 containing an *aadA* resistance cassette (not shown) and the genomic *PSB28* gene fused to the sequence coding for a C-terminal 3xHA tag as well as the *HSP70A-RBCS2* promoter and *RPL23* terminator. Total protein from wild type (WT), seven green transformants and the *psb28* mutant were analyzed by SDS-PAGE and immunoblotting using antisera against the D1 protein and the HA epitope. The transformants indicated in red (c2 and c6) were used for further experiments.
Supports Figure 1.

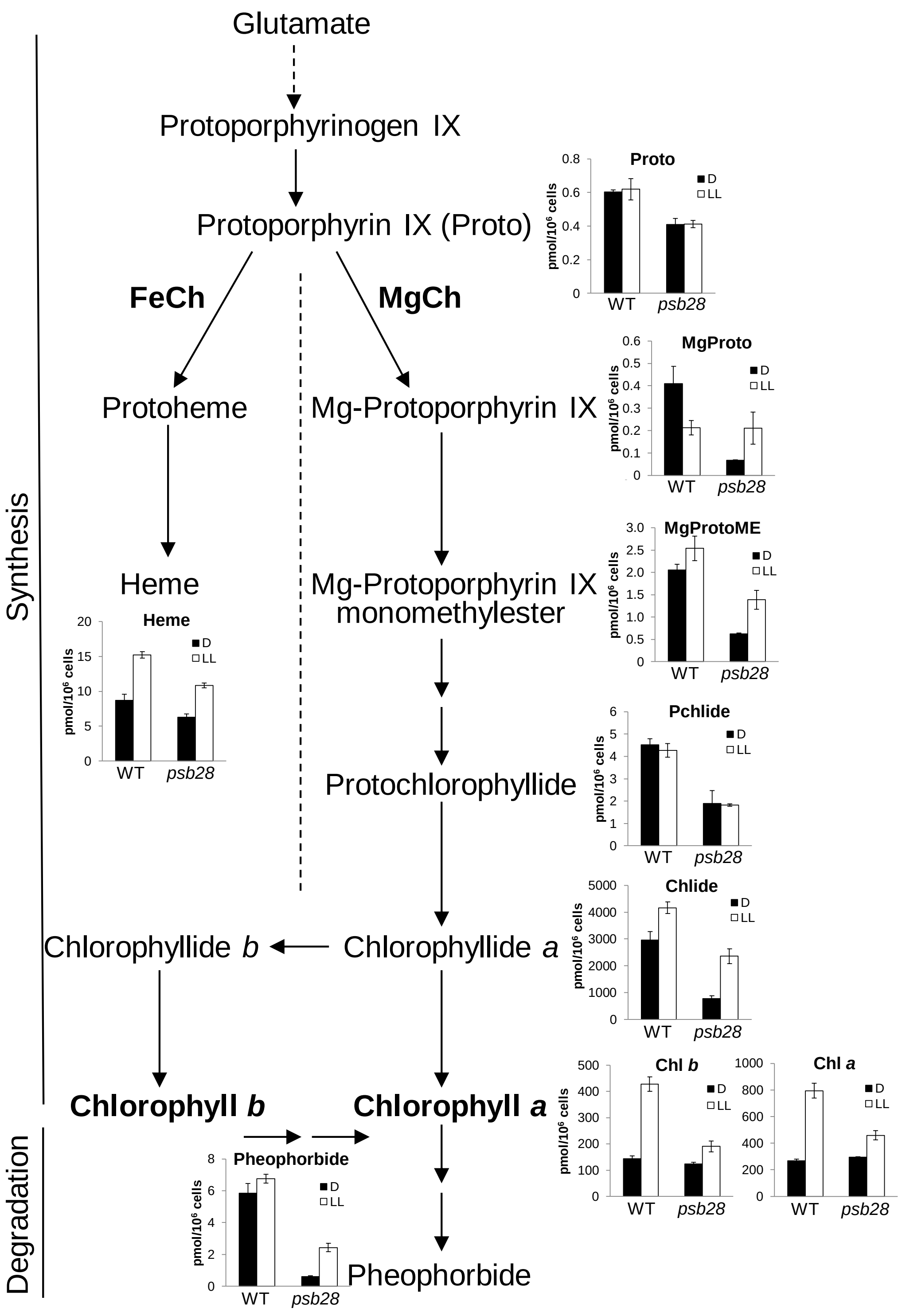


**Supplementary Figure S3. Analysis of chlorophyll precursor contents in psb28 mutant and WT.**

WT and *psb28* mutant were grown in low light (LL, 30 µmol photons $m^{-2}$ $s^{-1}$) or in the dark for 65 h (D) and pigments were extracted and analyzed by HPLC. Shown are mean values from three independent experiments, error bars represent standard deviation. Supports Figure 1.

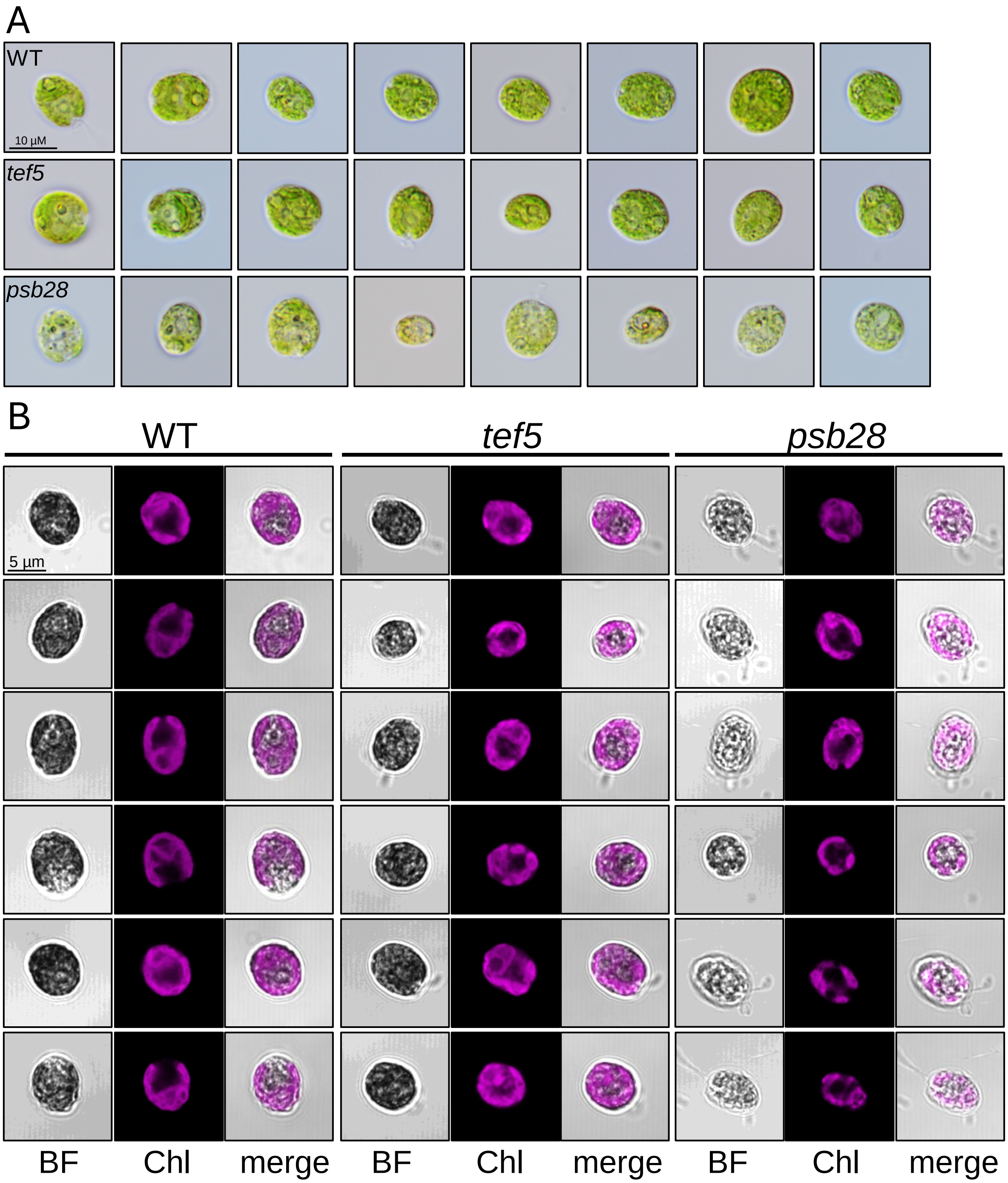


**Supplementary Figure S4. Collection of light microscopy and fluorescence microscopy images of WT, *tef5*, and *psb28*.**
**(A)** Light microscopy images.
**(B)** Confocal laser scanning microscopy showing bright field (BF), chlorophyll autofluorescence (Chl) and a merge of both.
Cells were grown under mixotrophic conditions in low light. The scale bars shown apply to all images of the respective panel. Supports Figures 2 and 9.

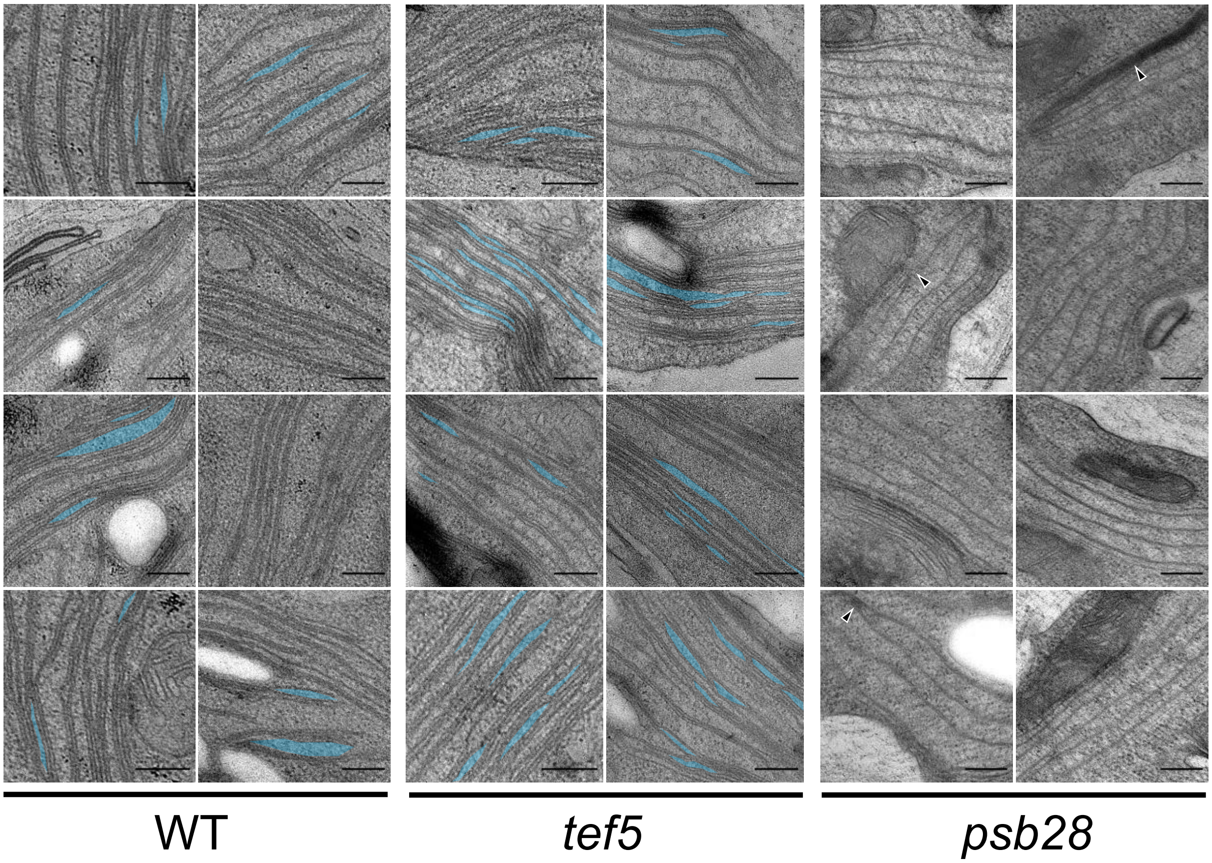


**Supplementary Figure S5. Collection of electron micrographs from thylakoids of WT, *tef5* and *psb28*.**

Cells were grown under mixotrophic conditions in low light. Blue areas indicate regions where thylakoid stacking is interrupted. Black triangles point to rarely occurring thylakoid membrane stacks in the *psb28* mutant. Scale bars correspond to 100 nm.

Supports Figures 2 and 9.

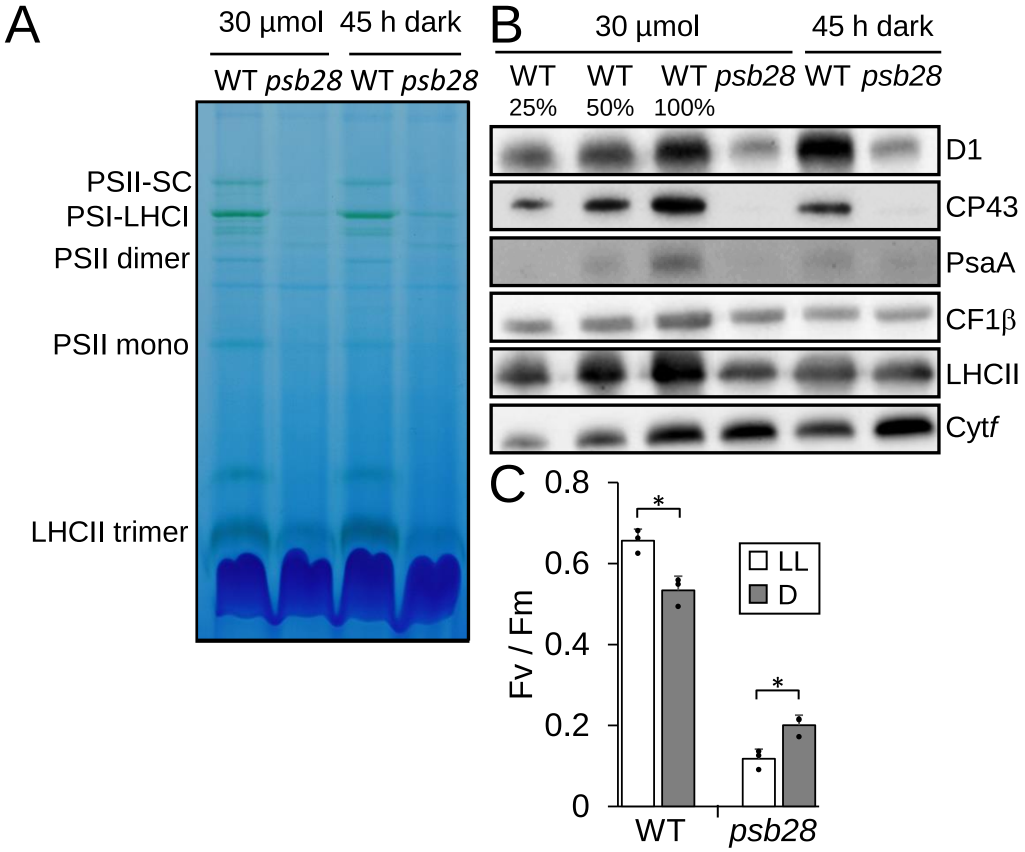


**Supplementary Figure S6. Analysis of PSII complex assembly, subunit accumulation, and functionality in dark-grown cells.**

**(A)** BN-PAGE analysis of cells grown in low light (30 µmol photons $m^{-2}s^{-1}$) and in darkness for 45 h. Whole-cell proteins from WT and *psb28* mutant were solubilized with 1% β-DDM. 50 µg of protein per lane were separated on a 4-15 % BN gel. Shown is a picture of the gel after the run.

**(B)** Immunoblot analysis of the accumulation of subunits of the major thylakoid membrane protein complexes in WT and *psb28* mutant grown in low light and in darkness for 45 h. PSII – D1, CP43, LHCII; PSI – PsaA; Cyt $b_6f$ complex – Cyt *f*; ATP synthase – CF1β. 10 µg of whole-cell proteins (100%) were loaded.

**(C)** $F_v/F_m$ values of the *psb28* mutant versus WT grown in low light (LL) and in darkness (D) for 45 h. Shown are averages from three independent experiments. Error bars represent standard deviation. Asterisks indicate significant differences between low light and dark (two-tailed, unpaired *t*-test, $P < 0.05$).

Supports Figure 4.

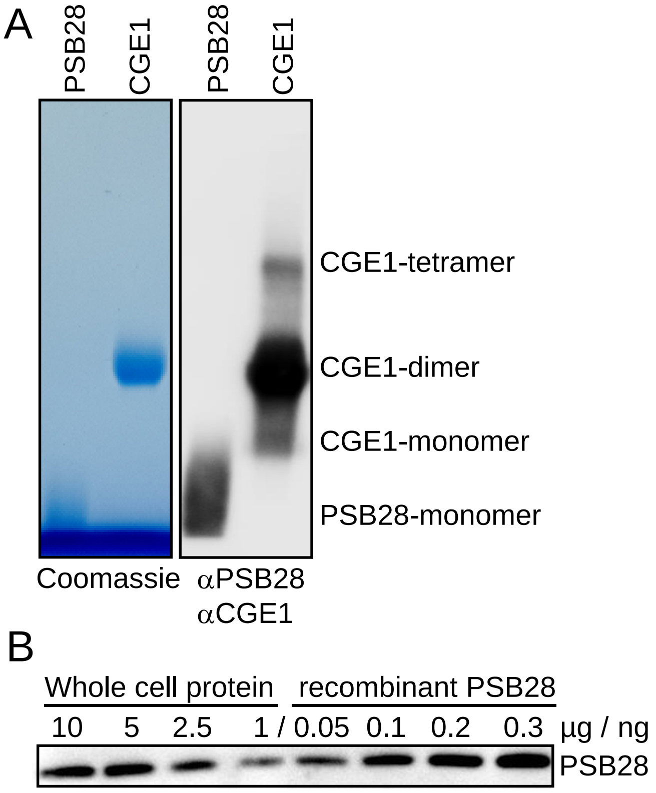


**Supplementary Figure S7. Analysis of oligomerization capacity of recombinant PSB28 and quantification of cellular PSB28 abundance.**
**(A)** 1 µg of recombinantly produced PSB28 and CGE1 were separated on a 4-15% BN gel and stained with Coomassie blue or subjected to immunoblot analysis using antisera against PSB28 and CGE1. The assignment of CGE1 oligomers is based on analyses by Schroda et al (2001) and Willmund et al. (2007).
**(B)** Quantitative immunoblot analysis. The indicated amounts of recombinant PSB28 and of *Chlamydomonas* total cell proteins were separated by SDS-PAGE and analyzed by immunoblotting using an antibody against PSB28. Shown is a representative experiment out of four. Quantification of the signals from four experiments indicated that PSB28 makes up 0.0034 ± 0.001 % of total cell proteins. Supports Figure 4.

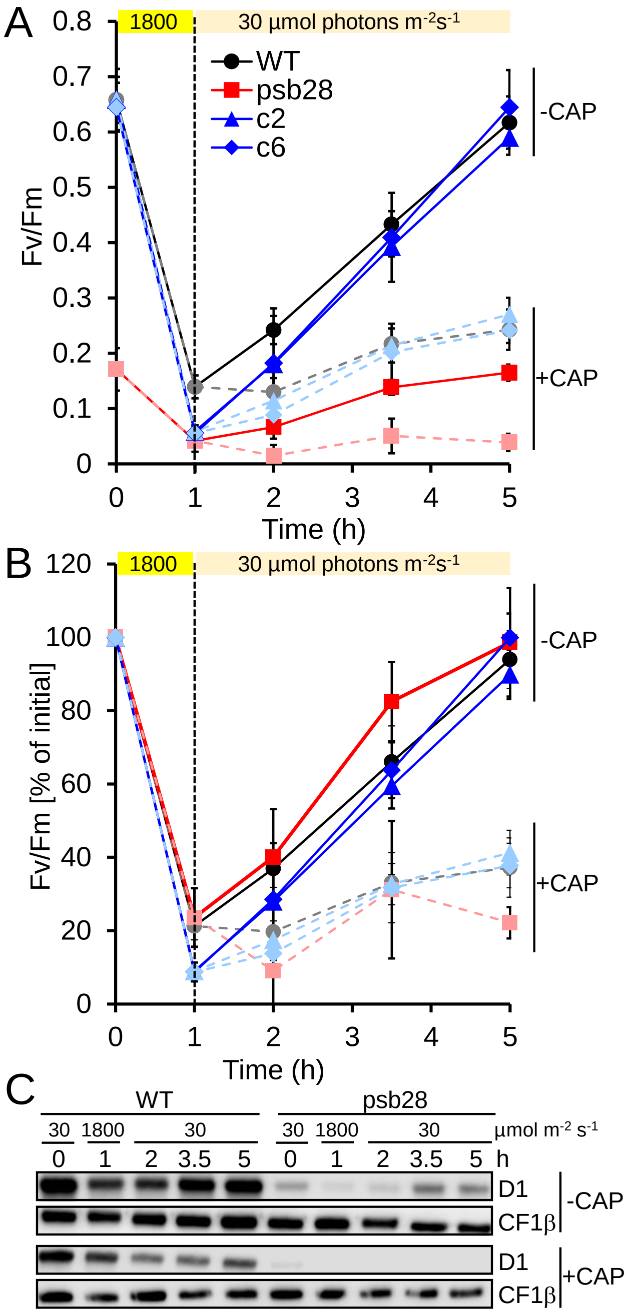


**Supplementary Figure S8. Monitoring kinetics of PSII repair after photoinhibition in the *psb28* mutant.**

**(A)** $F_v/F_m$ values of WT, *psb28* mutant, and complemented lines c2 and c6 after exposure to 1800 µmol photons $m^{-2}$ $s^{-1}$ for 1 h and recovery at 30 µmol photons $m^{-2}$ $s^{-1}$ for 5 h. Photoinhibition was performed in the presence of 100 µg/ml chloroplast translation inhibitor chloramphenicol (CAP), which was kept in half of the culture during recovery (pale colors, dashed lines) and removed from the other half (full colors, solid lines). Shown are averages from three independent experiments. Error bars represent standard deviation.

**(B)** $F_v/F_m$ values shown as % of initial values.

**(C)** Immunoblot analysis of 10 µg of whole cell proteins from one experiment to monitor the D1 levels. CF1β was used as loading control.

Supports Figure 4.

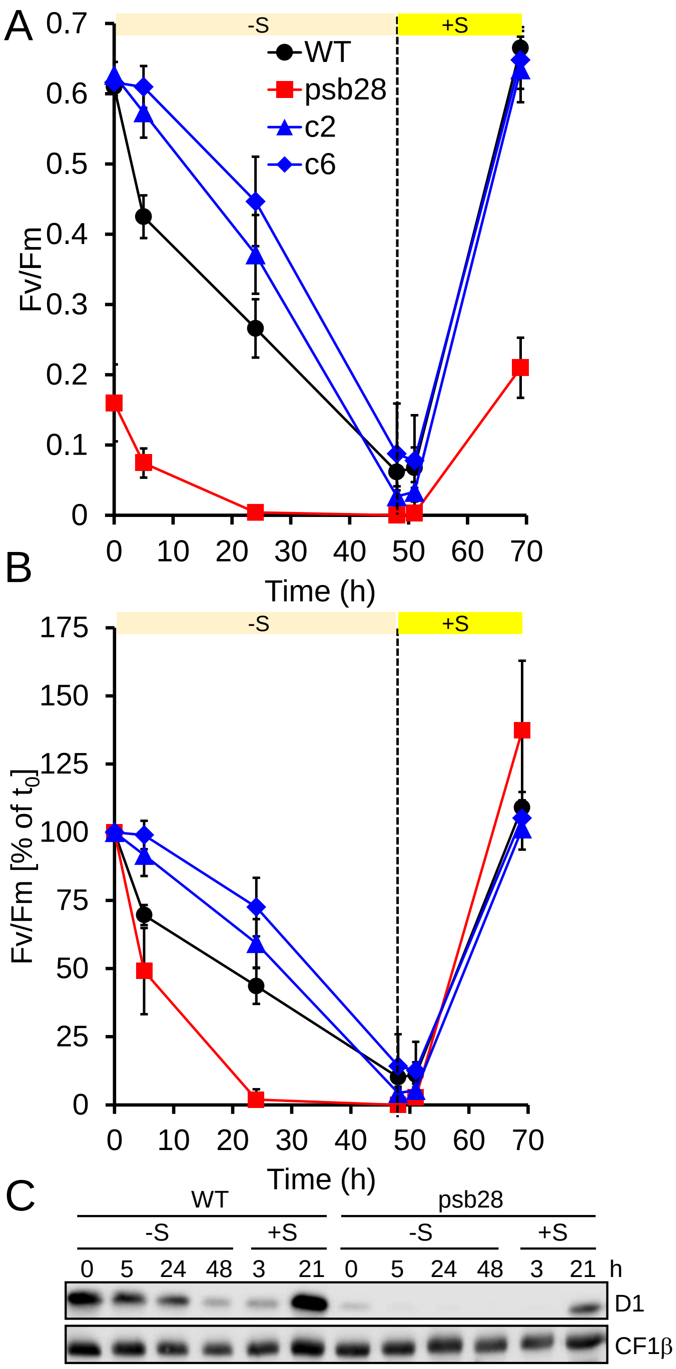


**Supplementary Figure S9. Monitoring kinetics of PSII re-synthesis in the *psb28* mutant after sulfur starvation.**

**(A)** $F_v/F_m$ values of WT, *psb28* mutant, and complemented lines c2 and c6 during cultivation in sulfur-depleted TAP medium for 48 h and during recovery in sulfur-replete TAP medium for 21 h. Shown are averages from three independent experiments. Error bars represent standard deviation.

**(B)** $F_v/F_m$ values shown as % of initial values.

**(C)** Immunoblot analysis of 10 µg of whole cell proteins from one experiment to monitor D1 levels. CF1β was used as loading control.

Supports Figure 4.

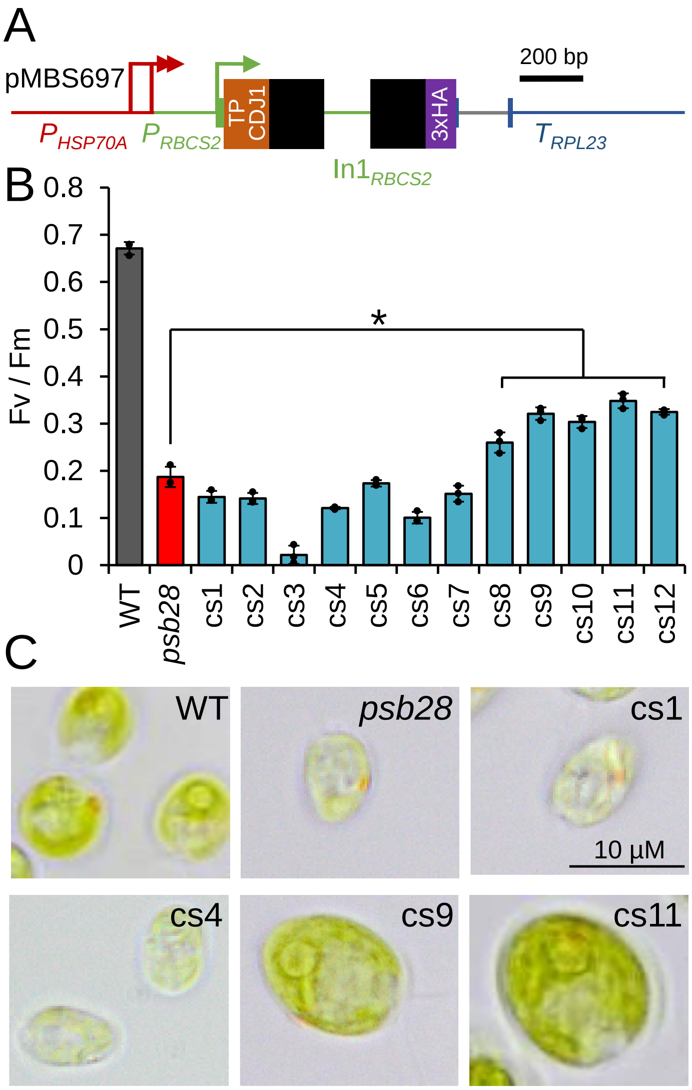


**Supplementary Figure S10. Construct for the expression of *Synechocystis* Psb28-1 and analysis of transformants in the *psb28* mutant background.**

**(A)** *psb28* mutant cells were transformed with construct pMBS697 containing an *aadA* resistance cassette (not shown). The codon-optimized sequence coding for *Synechocystis* Psb28-1 (black boxes) was fused to sequences encoding the CDJ1 chloroplast transit peptide (brown box), and the 3xHA tag (purple box). Expression is controlled by the *HSP70A* promoter (red) fused to the *RBCS2* promoter (green) and the *RPL23* terminator (blue). Untranslated regions are shown as bars, the 1$^{st}$ *RBCS2* intron (In1, green) and promoter regions as thin lines. Arrows indicate transcriptional start sites.

**(B)** $F_v/F_m$ values of WT (grey), *psb28* mutant (red), and 12 transformants in the *psb28* mutant background generated with the construct depicted in (A) (blue). Shown are averages from three independent experiments. Error bars represent standard deviation. The asterisk indicates significantly higher values for five transformants versus the *psb28* mutant (two-tailed, unpaired *t*-test with Bonferroni-Holm correction, $P < 0.05$).

**(C)** Light microscopy images of cells from WT, *psb28* mutant and transformants (cs1-12) generated with the construct shown in (A). The scale bar applies to all images displayed.

Supports Figure 5.

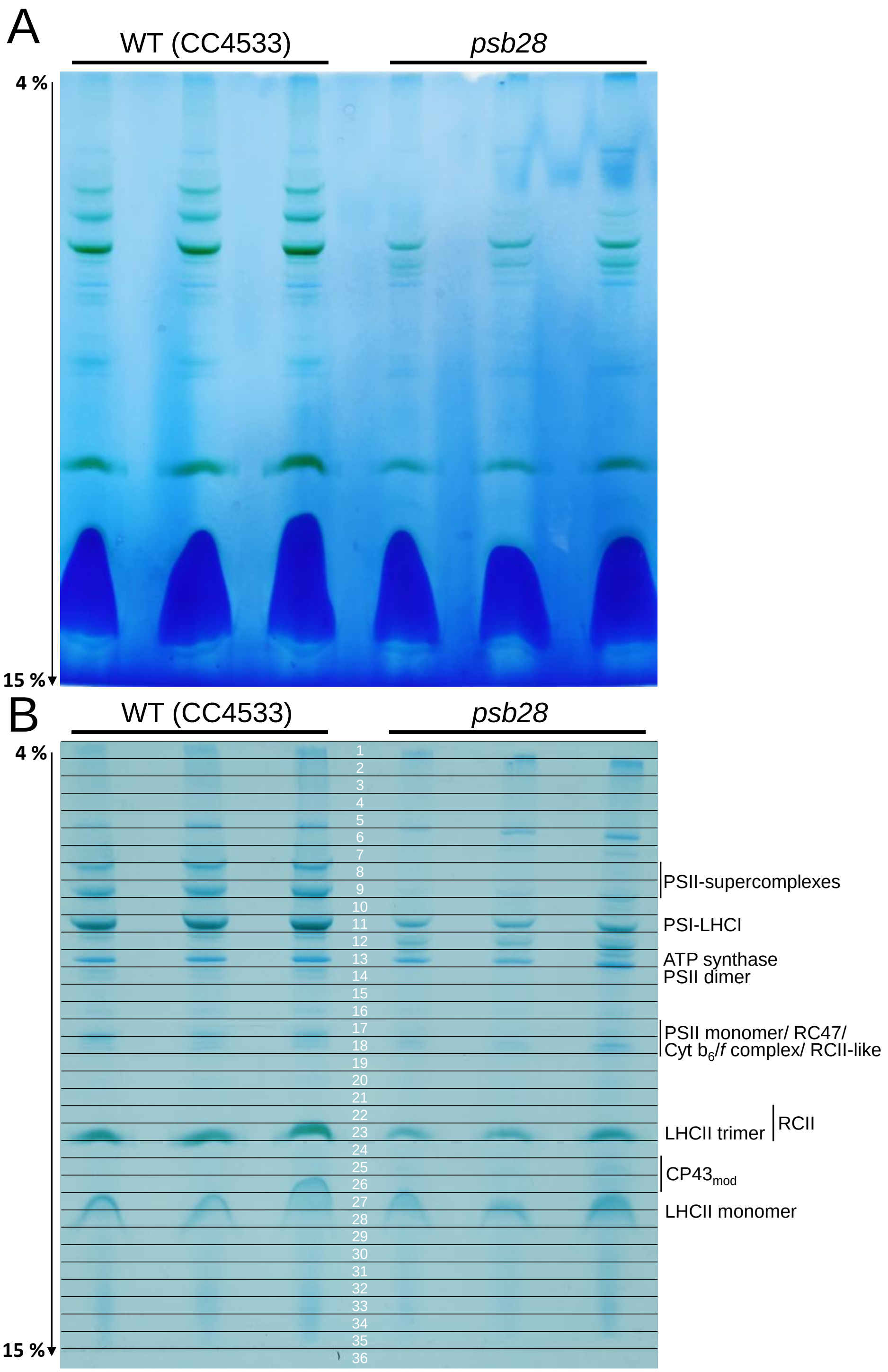


**Supplementary Figure S11. BN-PAGE for complexome profiling.** Thylakoid membranes were isolated from WT (CC4533) and *psb28* mutant cells, solubilized with n-dodecyl $\alpha$-D-maltoside, and separated on a 4% to 15 % BN gel. 60 µg of protein were loaded per lane.

**(A)** Photography of the gel right after the run.

**(B)** Coomassie staining of the gel shown in (A). Each lane was cut into 36 slices according to the grid shown and gel slices were subjected to tryptic in-gel digestion followed by LC-MS/MS. The identity of the indicated complexes derives from the mass spectrometry analysis (Supplementary Dataset S2).

Supports Figure 6.

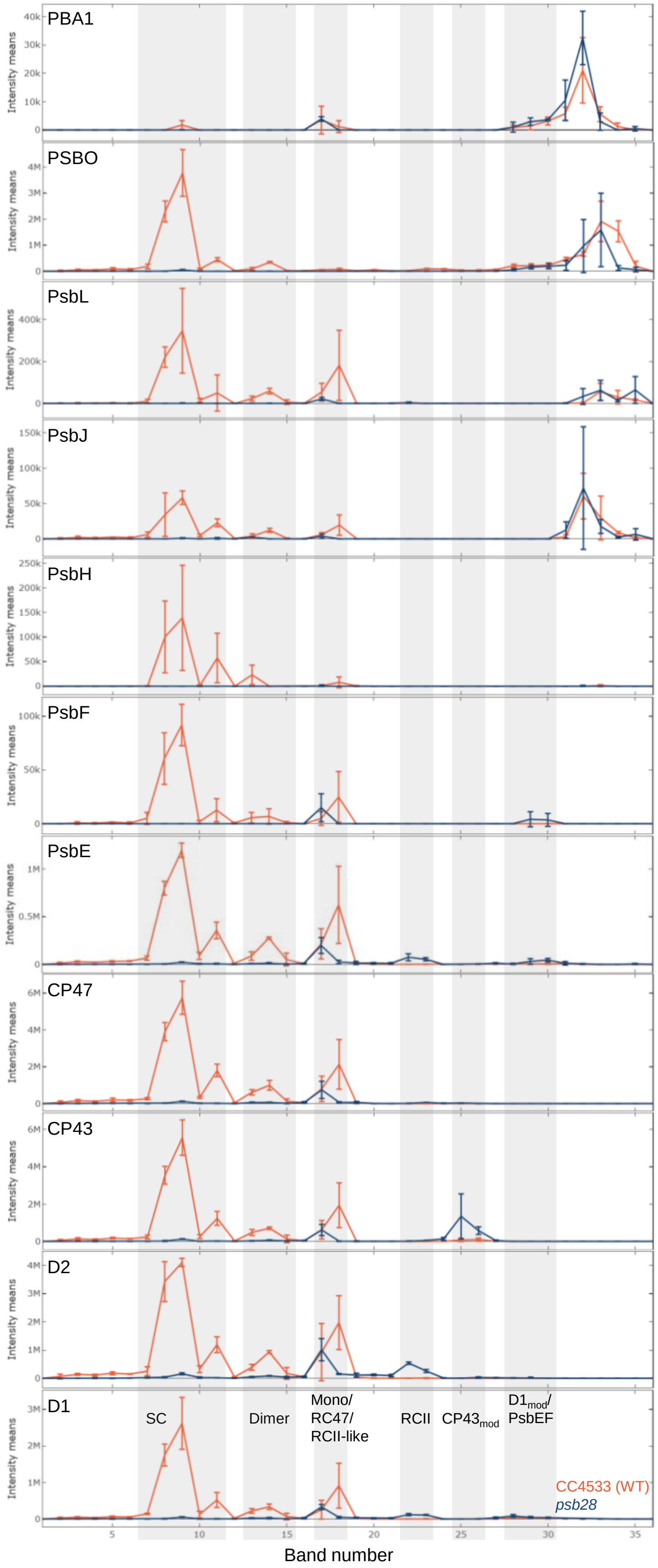


**Supplementary Figure S12. Comparison of BN-PAGE migration profiles of PSII core subunits and of putative novel PSII-associated protein PBA1.** Values for each protein from WT (red) and *psb28* mutant (blue) are derived from averaged peptide ion intensities from three biological replicates after normalization based on the abundance of ATP synthase subunits. Error bars represent SD. Individual profiles from each replicate before and after normalization and statistical analyses can be accessed in Supplementary Dataset S2. SC – supercomplexes; RC – reaction center. Supports Figure 6.

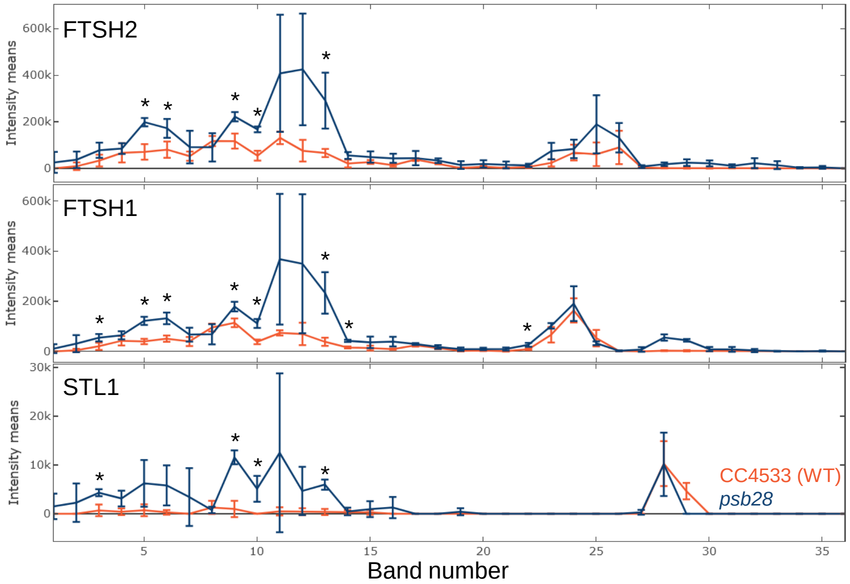


**Supplementary Figure S13. Comparison of BN-PAGE migration profiles of thylakoid membrane protease FTSH1/2 and kinase STL1.** Values for each protein from WT (red) and *psb28* mutant (blue) are derived from averaged peptide ion intensities from three biological replicates after normalization based on the ATP synthase. Error bars represent SD. Asterisks indicate significant differences of ion intensities between mutant and WT in the respective bands (two-tailed, unpaired *t*-test, $P < 0.05$).

Supports Figure 7.

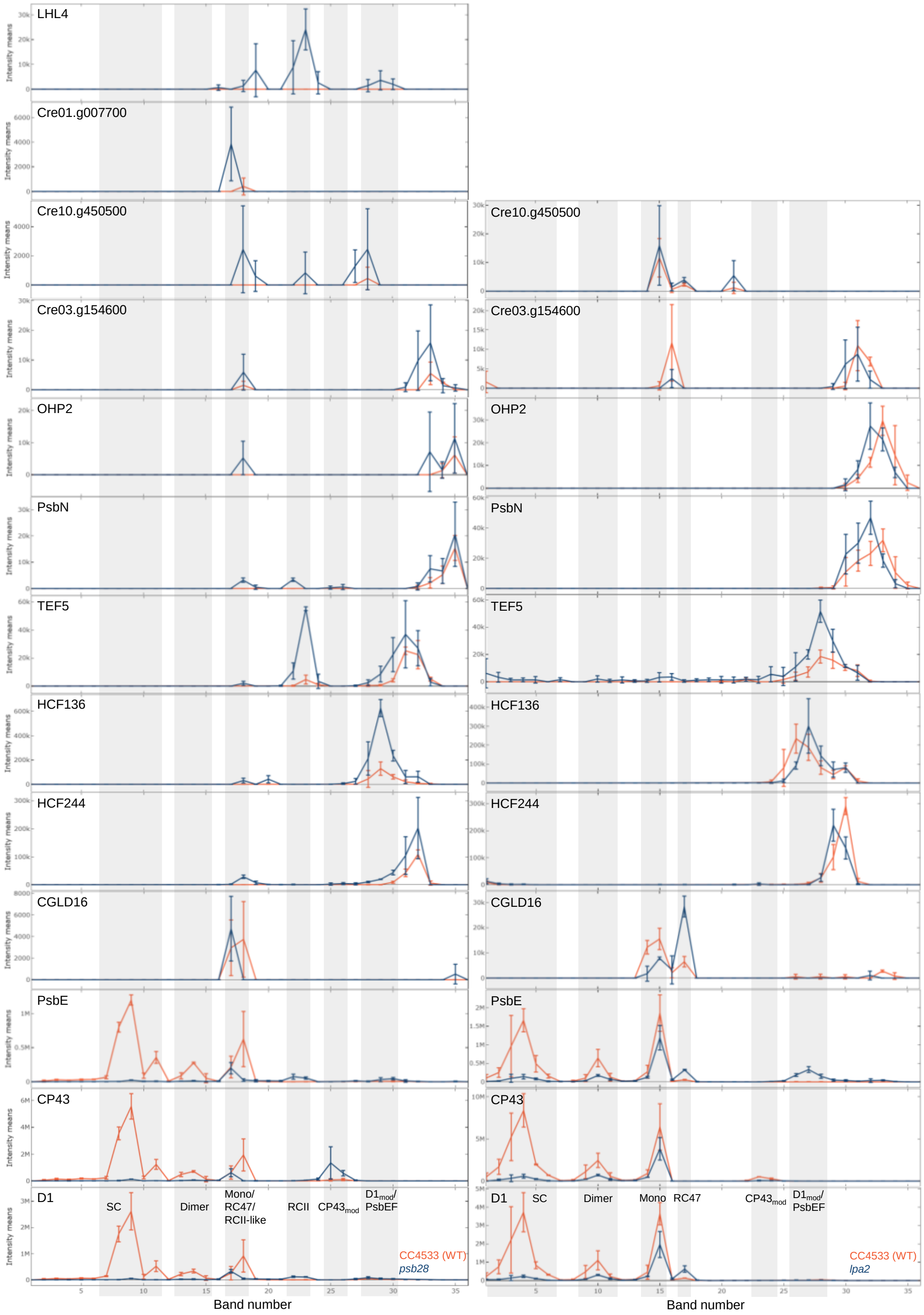


**Supplementary Figure S14. Comparison of BN-PAGE migration profiles of PSII core subunits, of known PSII auxiliary factors, and of putative novel auxiliary factors.** Putative novel auxiliary factors are chloroplast proteins accumulating in bands 17/18 (PSII monomers/RC47) and/or 22/23 (RC) only in the *psb28* mutant. Co-migration profiles on the left are from the *psb28* mutant (blue) and WT (red), those on the right from the *lpa2* mutant (blue) and WT (red) (Spaniol et al., 2022). Values for each protein are derived from averaged peptide ion intensities from three biological replicates after normalization. Error bars represent SD. SC – supercomplexes; RC – reaction centers. Supports Figure 7.

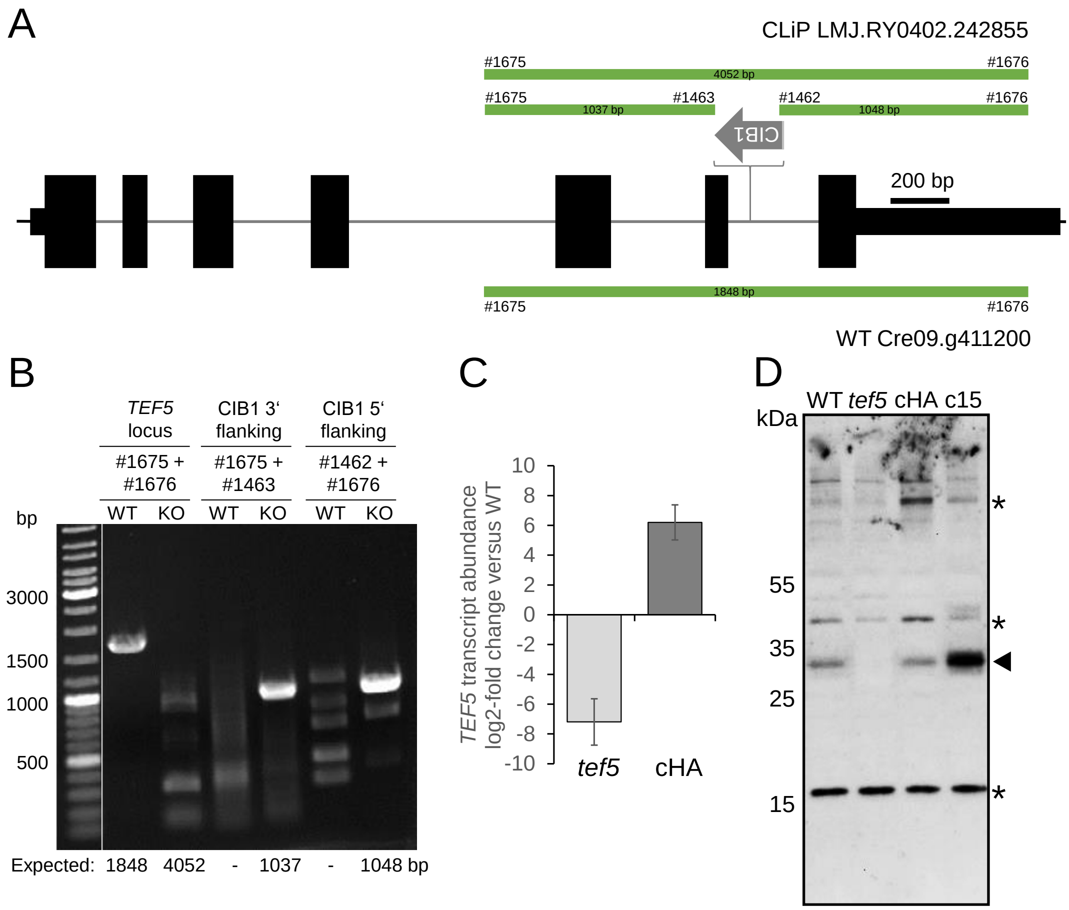


**Supplementary Figure S15. Analysis of the CIB1 integration site in the *TEF5* gene by PCR and of TEF5 protein in the *tef5* mutant and in complemented lines.**

**(A)** Gene model of the *TEF5* gene with exons shown as black boxes and introns as thin grey lines. The integration site of the CIB1 cassette (https://www.chlamylibrary.org/showCassette?cassette=CIB1) in the sixth intron is shown. Green bars indicate the expected PCR products on mutant DNA (CLiP, top) and WT DNA (bottom). Numbers in the bars indicate their sizes. Numbers flanking the bars are the primer numbers (Supplementary Table S1).

**(B)** PCR products on genomic DNA from WT and the *tef5* mutant from the CLiP collection (KO) were separated on an agarose gel and stained with Gel Red. Expected amplicon sizes are indicated below the gel.

**(C)** Analysis of *TEF5* transcript abundance in *tef5* mutant and transformant cHA generated with pMBS756 compared with the WT. *CBLP2* was used as a housekeeping control. Shown are qRT-PCR data from three biological replicates, each derived from 3 technical replicates. Error bars show SD.

**(D)** Immunoblot analysis to test the TEF5 peptide antibody. 10 µg of whole-cell proteins from WT, *tef5* mutant and complemented lines *tef5*-cHA and *tef5*-c15 were separated on a 12% SDS-polyacrylamide gel and analyzed by immunoblotting using a 1:500 dilution of the TEF5 antibody. Asterisks indicate cross-reactions, the arrowhead the expected position of the TEF5 protein band at ~27.5 kDa.

Supports Figure 8.

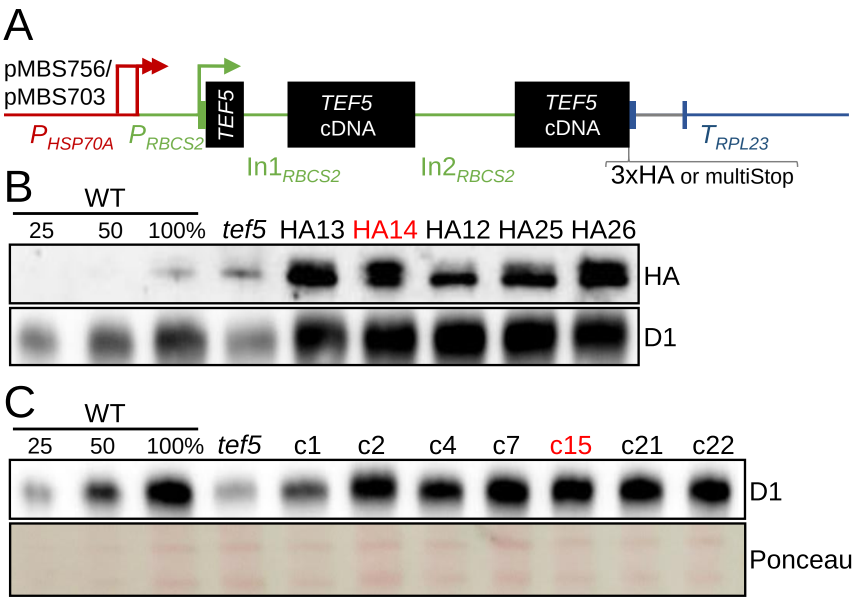


**Supplementary Figure S16. Screening for complemented *tef5* transformants.**
**(A)** Constructs for complementation (see legend of Figure 8C).
**(B)** The *tef5* mutant was transformed with construct pMBS756. Whole-cell proteins from WT, the *tef5* mutant and five spectinomycin-resistant transformants were analyzed by SDS-PAGE and immunoblotting using antibodies against the HA epitope and the D1 protein, respectively. Transformant HA14 (cHA) was used for further experiments.
**(C)** *tef5* mutant cells were transformed with construct pMBS703. Whole-cell proteins from WT, the *tef5* mutant and seven spectinomycin-resistant transformants were analyzed by SDS-PAGE and immunoblotting using a D1 antibody. Transformant c15 was used for further experiments.
Supports Figure 8.

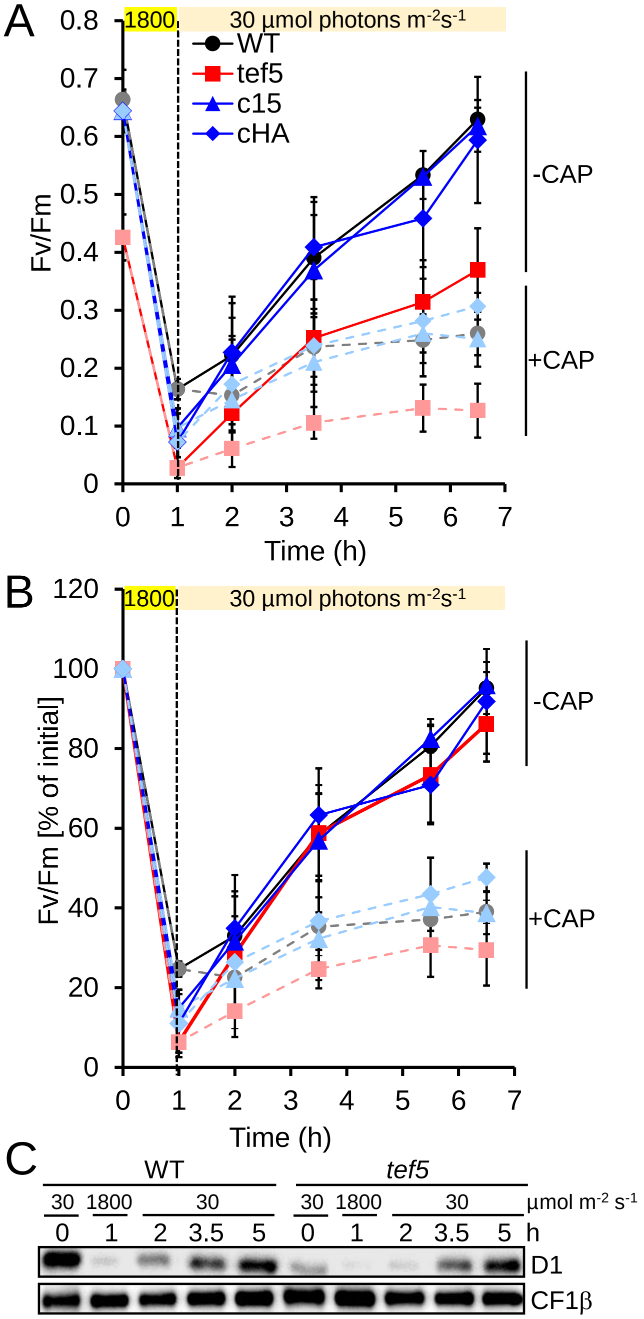


**Supplementary Figure S17. Monitoring kinetics of PSII repair after photoinhibition.**
**(A)** $F_v/F_m$ values of WT, *tef5* mutant, and complemented lines *tef5*-c15 and *tef5*-cHA after exposure to 1800 µmol photons $m^{-2}$ $s^{-1}$ for 1 h and recovery at 30 µmol photons $m^{-2}$ $s^{-1}$ for 6.5 h. Photoinhibition was performed in the presence of 100 µg/ml chloroplast translation inhibitor chloramphenicol (CAP), which was kept in half of the culture during recovery (pale colors, dashed lines) and removed from the other half (full colors, solid lines). Shown are averages from three independent experiments. Error bars represent standard deviation.
**(B)** $F_v/F_m$ values shown as % of initial values.
**(C)** Immunoblot analysis of 10 µg of whole cell proteins from one experiment with recovery in the absence of CAP to monitor the D1 levels. CF1β was used as loading control.
Supports Figure 11.

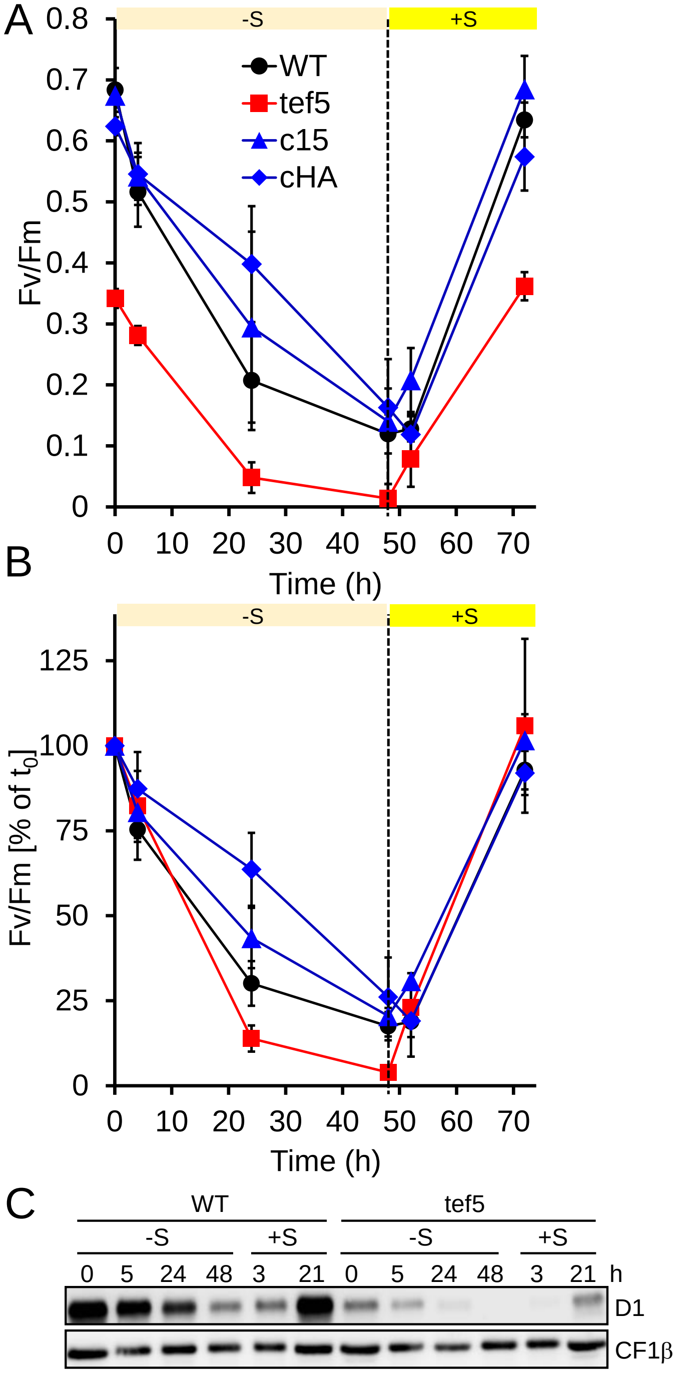


**Supplementary Figure S18. Monitoring kinetics of PSII re-synthesis in the *tef5* mutant after sulfur starvation.**

**(A)** $F_v/F_m$ values of WT, *tef5* mutant, and complemented lines *tef5*-c15 and *tef5*-cHA during cultivation in sulfur-depleted TAP medium for 48 h and during recovery in sulfur-replete TAP medium for 21 h. Shown are averages from three independent experiments. Error bars represent standard deviation.

**(B)** $F_v/F_m$ values shown as % of initial values.

**(C)** Immunoblot analysis of 10 µg of whole cell proteins from one experiment to monitor D1 levels. CF1β was used as loading control.

Supports Figure 11.

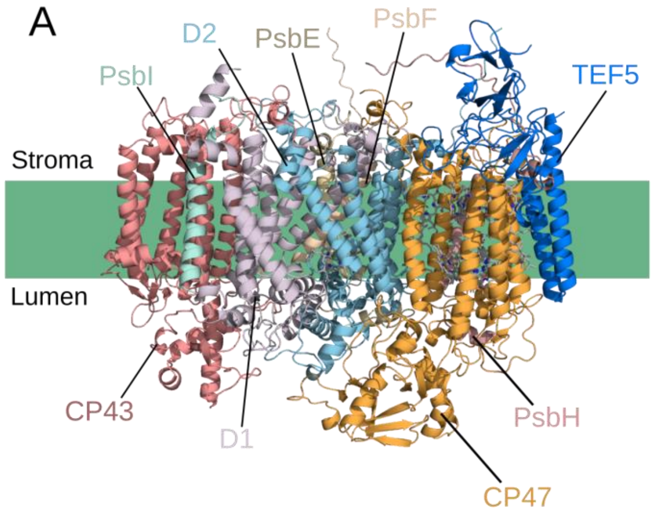


B

| TEF5 | CP47 | Interaction |
|---|---|---|
| Ala120 | Arg18 | spVDW |
| Phe170 | Tyr117 | spVDW |
| Asn214 | Asn14 | HBO (backbone-sidechain) |
| Phe216 | Asn14 | HBO (backbone-sidechain) |
| Phe216 | Arg476 | spVDW |
| Val218 | Asn14 | HBO (backbone-sidechain) |
| Trp282 | Phe151 | spVDW |
| **TEF5** | **D2** | **Interaction** |
| Asp239 | Lys11 | HBO (sidechain-sidechain) |
| **TEF5** | **PsbH** | **Interaction** |
| Tyr223 | Gly38 | HBO (backbone-sidechain) |
| Tyr223 | Val40 | HBO (backbone-backbone) |
| Glu225 | Lys39 | HBO (sidechain-sidechain) |

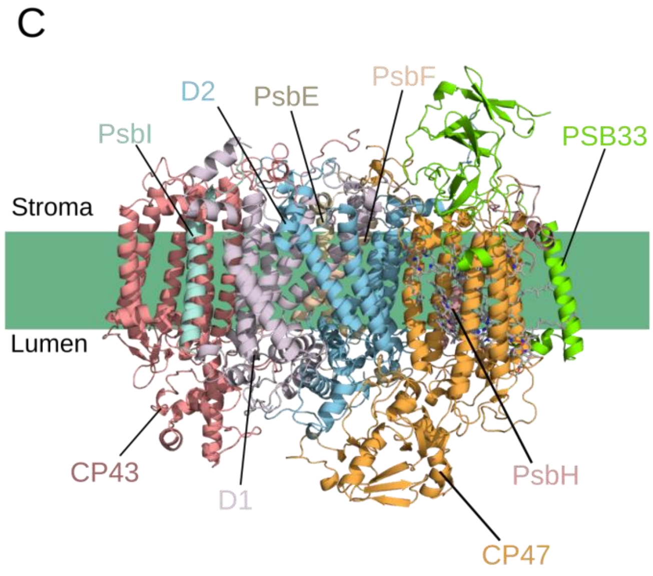


D

| PSB33 | D1 | Interaction |
|---|---|---|
| Thr219 | Asn230 | HBO (backbone-sidechain) |
| Val221 | Asn230 | HBO (backbone-sidechain) |
| **PSB33** | **Cp47** | **Interaction** |
| Ala126 | Asp15 | HBO (backbone-sidechain) |
| Tyr127 | Val11 | spVDW |
| Asn222 | Asn14 | HBO (backbone-sidechain) |
| Asp224 | Asn14 | HBO (backbone-sidechain) |
| Asp224 | Lys130 | HBO (sidechain-sidechain) |
| Glu225 | Arg230 | HBO (sidechain-sidechain) |
| Val226 | Tyr226 | HBO (backbone-sidechain) |
| Phe261 | Phe156 | spVDW |
| Thr273 | Trp185 | HBO (backbone-sidechain) |
| Phe275 | Trp185 | spVDW |
| **PSB33** | **PsbH** | **Interaction** |
| Glu103 | Arg11 | HBO (sidechain-sidechain) |
| Ile229 | Gly31 | HBO (backbone-backbone) |
| Ile229 | Val33 | HBO (backbone-backbone) |
| Asp231 | Val33 | HBO (backbone-backbone) |
| Lys242 | Glu29 | HBO (sidechain-sidechain) |

**Supplementary Figure S19. Predicted TEF5/PSB33-PSII core complexes.**
**(A)** Predicted structural model of TEF5 (blue) in complex with the PSII subunits D1 (light purple), D2 (light blue), CP47 (orange), CP43 (light red), PsbE (yellow), PsbF (light orange), PsbH (pink), and PsbI (cyan) in Chlamydomonas. **(B)** List of identified hydrogen bonds (HBO) and specific van-der-Waals interactions (spVDW) between TEF5 and PSII subunits. **(C)** Predicted structural model of PSB33 (green) in complex with PSII (colored as in (A)) in Arabidopsis. **(D)** List of identified hydrogen bonds (HBO) and specific van-der-Waals interactions (spVDW) between PSB33 and PSII subunits.
Supports Figure 11.

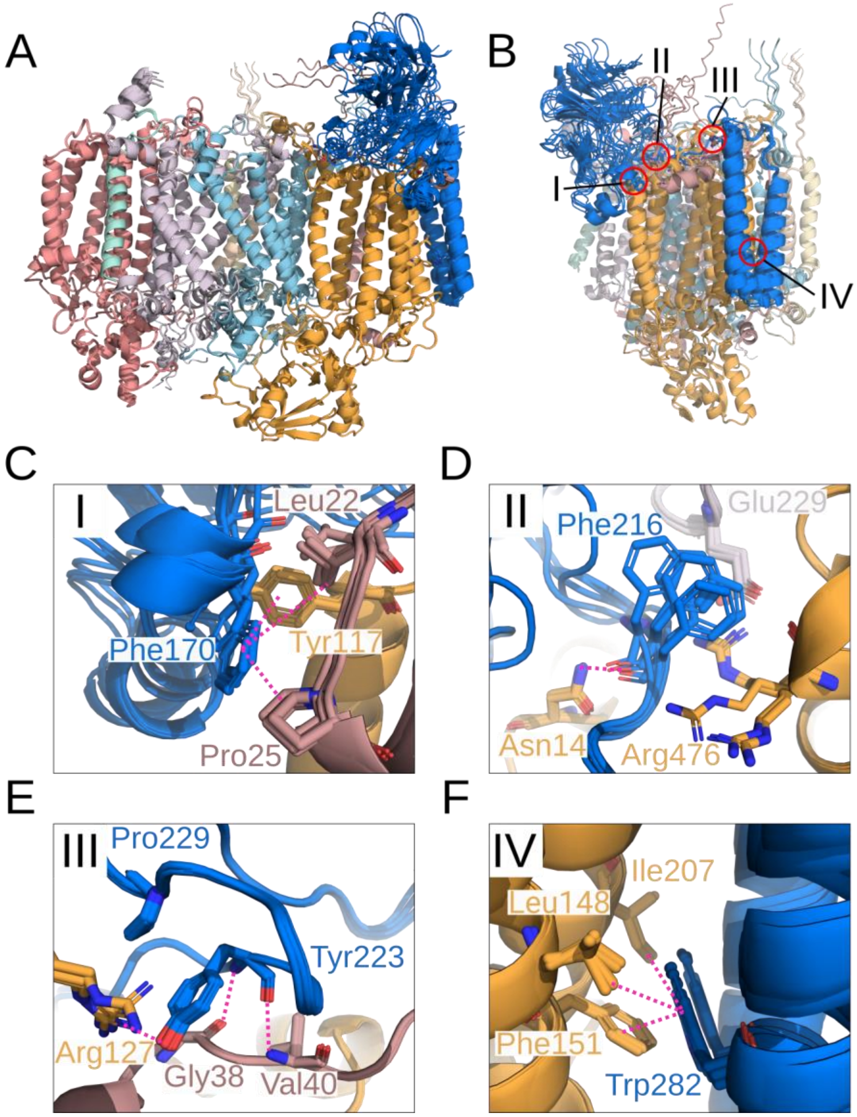


**Supplementary Figure S20. Predicted contact sites of TEF5 with CP47 and PsbH in Chlamydomonas PSII.**

**(A)** Front view of the superposition of the five predicted structural model of TEF5 (blue) in complex with the PSII subunits D1 (light purple), D2 (light blue), CP47 (orange), CP43 (light red), PsbE (yellow), PsbF (light orange), PsbH (pink), and PsbI (cyan) in Chlamydomonas.

**(B)** Side view of the superposition of the five predicted structural models of TEF5 in complex with the PSII intermediate (colored as in (A)). Highlighted are the identified four key specific interactions used to propose TEF5 destabilizing point mutants.

**(C,D,E,F)** Enlargement of the mutation sites. Interactions between the residues are highlighted in magenta. Supports Figure 11.

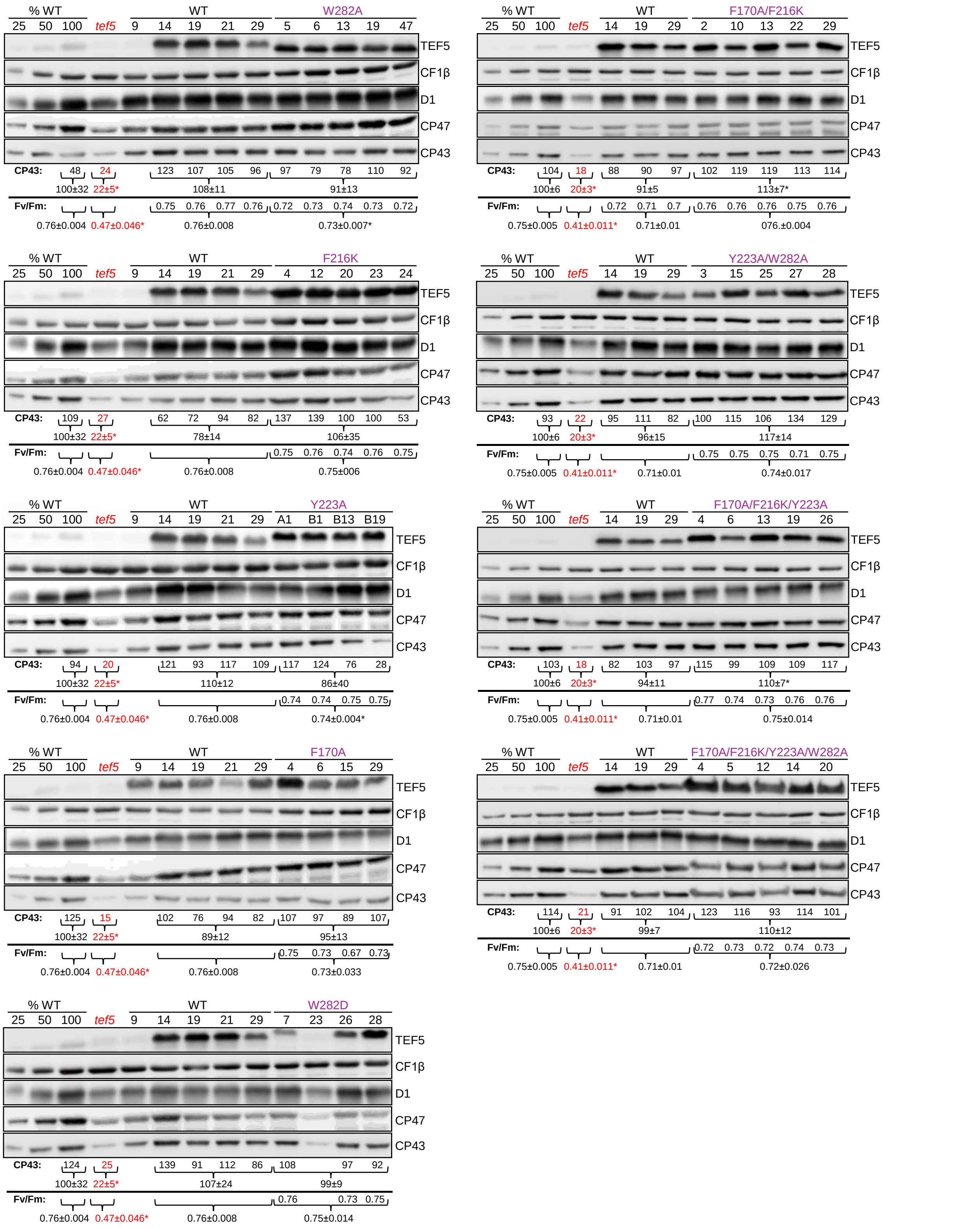


**Supplementary Figure S21. Complementation analysis of the *tef5* mutant with TEF5 carrying amino acid exchanges at residues predicted to mediate its interaction with CP47.**

The *tef5* mutant was transformed with pMBS703 (Figure 8C) carrying point mutations leading to the indicated amino acid exchanges (purple) in the TEF5 protein. The accumulation of D1, CP47, CP43, and

CF1β as loading control were analyzed in 3-5 transformants producing the TEF5 variants and WT TEF5. Moreover, Fv/Fm values were determined. The values below the immunoblots indicate the quantified CP43 signals for WT, *tef5* and the individual transformants after normalizing first by the median of all CP43 signals from the same blot and then by the mean signal obtained for WT. The values below the round brackets indicate the means (with SD) of five (left panels) and four (right panels) signals for WT and *tef5*, and the means of the values obtained for the 3-5 transformants producing WT and mutated TEF5. Values for Fv/Fm are means of five (left panels) and three (right panels) biological replicates of WT and *tef5* and means of the transformants producing the same TEF5 variant. Asterisks indicate significant differences with respect to the WT (two-tailed, unpaired t-test with Bonferroni-Holm correction, $P < 0.05$). The absence of an asterisk means that there were no significant differences.
Supports Figure 11.

```
CreLHL4 -----------------AEKSGFAKWADSVGMDSSDGVFGFTPFAETWVGRWSMMGFVSSIVVEFATGKGTLAQVGLD : 61
DsaLHL4 --------AQQSSEKPNAQNDPVLAYAESIGLPTEEGVFGFKPFSEIWCGRLAMMGFLVSIVEEFGTGQVGVYRLEVP : 70
CrePSBS AASTKVNPKLASKTEVERFKQATGLPAPAINGKQFPLKLGFTKTNELFVGRLAMVGFSASLIGEILTGKGALAQFGYE : 78
DsaPSBS ----------------AIFKGQDKKSKEAPPPKQKPLRLGFTKDNELFVGRAAMLGFAFSLIGEVLTGKGALAQLGYE : 62
SynHliA --------MTTRGFRLDQDNRLNNFAIEPEVYVDSSVQAGWTKYAEKMNGRFAMIGFASLLIMEVVTGHGVIGWLNSL : 70
SynHliB --------MTSRGFRLDQDNRLNNFAIEPPVYVDSSVQAGWTEYAEKMNGRFAMIGFVSLLAMEVITGHGIVGWLLSL : 70
SynHliC -------------------------------MNNENSKFGFTAFAENWNGRLAMIGFSSALILELVSGQGVLHFFGIL : 47
SynHliD ---------------------MSEELQPNQTPVQEDPKFGFNNYAEKLNGRAAMVGFLLILVIEYFTNQGVLAWLGLR : 57
                                                       ______TMH______
```

**Supplementary Figure S22. Alignment of N-terminal regions of green algal LHL4 and PSBS proteins with cyanobacterial HliA-D.**

Alignment of amino acid sequences of the N-terminal regions of LHL4 and PSBS sequences from *Chlamydomonas reinhardtii* (Cre) and *Dunaliella salina* (Dsa) lacking predicted transit peptides with HliA-D from *Synechocystis sp.* (strain PCC 6803) (Syn). Residues highlighted in black, dark gray and light gray are conserved in eight, six to seven, and five of the sequences, respectively. Residues in predicted transmembrane helices (TMH) are shown in blue. CreLHL4 (Cre17.g740950), DsaLHL4 (KAF5826794), CrePSBS (Cre01.g016600), DsaPSBS (KAF5841540), SynHliA (P73183), SynHliB (P73429), SynHliC (P73563), SynHliD (P72932).
Supports Discussion.

**Supplementary Table S1.** Proteins involved in PSII assembly, repair, or complex dynamics in Arabidopsis or *Synechocystis* that have clear homologs in Chlamydomonas and are present with three replicates each for WT and *psb28* mutant in the complexome profiling dataset. Ratios in bold with asterisk accumulate to significantly different levels (P < 0.05). Ath – *Arabidopsis thaliana*; Syn – *Synechocystis*; Cre – *Chlamydomonas reinhardtii*.

| Locus Ath | Name Ath (Syn) | Name Cre | Locus Cre | Ratio psb28/WT | Description from Lu (2016) for Ath homolog |
|---|---|---|---|---|---|
| At2g47450 | cpSRP43 | SRP43 | Cre04.g231026 | 0.52 | Insertion and assembly of PSII proteins such as D1, D2, and CP47, and LHCII subunits |
| At2g28800 | ALB3 | ALB3.1 | Cre06.g251900 | 0.48 | Insertion and assembly of PSII proteins such as D1, D2, and CP47, and LHCII subunits |
| | | ALB3.2 | Cre17.g729800 | **1.75*** | |
| At2g18710 | cpSecY1 | SECY1 | Cre16.g681900 | 1.53 | Insertion and assembly of PSII proteins such as PsbO |
| At5g28750 | Tha4 | TATA | Cre10.g438550 | 2.76 | Insertion and assembly of PSII proteins such as PsbP and PsbQ |
| At5g52440 | HCF106 | TATB | Cre08.g371650 | 2.71 | Insertion and assembly of PSII proteins such as PsbP and PsbQ |
| At1g05810 | cpRabA5e | RAB11 | Cre03.g189250 | 3.12 | Transport of PSII proteins such as LHCB1, LHCB3 and CP47 to and from thylakoids |
| At2g20890 | THF1 (Psb29) | THF1, PSB29 | Cre13.g562850 | 2.37 | Dynamics of PSII-LHCII supercomplexes |
| At5g12130 | TERC | TERC | Cre17.g712400 | 3.39 | Co-translational insertion of PSII proteins such as D1, D2, and CP43 |
| At5g01920 | STN8 | STL1 | Cre12.g483650 | **3.84*** | Phosphorylation of D1, D2, CP43, and PsbH |
| At4g17600 | SEP3.1/LIL3.1 | LHL3, LIL3 | Cre03.g199535 | 1.59 | Anchoring geranylgeranyl reductase to thylakoid membranes; stabilizing LHCII |
| At4g35250 | HCF244 (Ycf39) | HCF244, CGL102 | Cre02.g142146 | 2.54 | Translational initiation of the psbA mRNA |
| At1g02910 | LPA1 (PratA) | REP27, LPA1 | Cre10.g430150 | 1.72 | Biogenesis and assembly of the D1 protein |
| At1g55480 | MET1 | TEF30 | Cre01.g031100 | **0.28*** | Supercomplex formation in PSII repair |
| At1g77510 | PDI6/PDIL1-2 | RB60 | Cre02.g088200 | 2.49 | Regulation of D1 synthesis |
| At4g35760 | LTO1 | CPLD41, VKE1, LTO1 | Cre12.g493150 | **1.67*** | Disulfide bond formation in PsbO |
| At3g01480 | CYP38/TLP40 | CYN38, TLP40 | Cre03.g189800 | 3.55 | PPIase; Inhibiting dephosphorylation of PSII subunits during PSII repair; conversion of PSII core monomers to PSII supercomplexes |
| At1g54780 | TLP18.3 | TEF8, TLP18.3 | Cre03.g182150 | 2.51 | D1 degradation and PSII dimerization; dephosphorylation of PSII core proteins (e.g.,D1 and D2) |
| At1g50250 | FtsH1 | FTSH1 | Cre12.g485800 | **2.44*** | Degradation of photodamaged D1 |
| At2g30950 | FtsH2/VAR2 | FTSH2 | Cre17.g720050 | **2.52*** | Chloroplast biogenesis; thylakoid formation; degradation of photodamaged D1 |
| At1g03600 | PSB27 | CPLD45, PSB27 | Cre05.g243800 | 1.63 | C-terminal processing of D1 during PSII repair? |
| At5g23120 | HCF136 (Ycf48) | HCF136 | Cre06.g273700 | **4.87*** | Assembly of PSII reaction-center complexes such as RC, RC47a, and RC47b |
| AtCg00700 | PsbN/PBF1 | PsbN | cp-encoded | 1.87 | Assembly of PSII minimal reaction-center complexes; regulation of PSII core and antenna protein phosphorylation |
| At5g51545 | LPA2 | LPA2 | Cre02.g105650 | 0.98 | Synthesis and assembly of CP43 |
| At1g71500 | PSB33/LIL8 | TEF5 | Cre09.g411200 | 2.71 | Association of LHCII with PSII |
| At1g67700 | HHL1 | TEF10a | Cre03.g146167 | 3.09 | Reassembly of PSII core monomers and PSII-LHCII supercomplexes during PSII repair |
| At1g34000 | OHP2 | OHP2 | Cre06.g251150 | 3.39 | Stabilization of D1 |
| At5g38660 | APE1 (Slr0575) | TEF6 | Cre16.g665250 | 3.29 | Protects PSII from over-excitation |

| | | | | | |
|---|---|---|---|---|---|
| At4g02530 | MPH2 | TEF14 | Cre06.g256250 | 2.07 | Dissociation of PsbO from damaged PSII core complex, dissociation of PSII dimers into monomers |
| At5g07020 | MPH1 (Psb34) | PRF1 | Cre03.g164300 | 1.14 | Associating with CP47 and preventing peripheral LHCs such as CP29/Hlip from associating with the PSII core |
| | | PRF2, ULP1 | Cre01.g051500 | **0.1*** | Restriction of accessibility of QB site to PQ in PSII monomer to promote charge recombination between QA- and TyrZ/P60+ when donor and acceptor sides are inactivated |

**Supplementary Table S2.** Primers used for genotyping, cloning, and RT-PCR.

| Primer | Sequence | Target |
|---|---|---|
| 1462, OMJ913 | 5'-GCACCAATCATGTCAAGCCT-3' | CIB1 |
| 1463, OMJ944 | 5'-GACGTTACAGCACACCCTTG-3' | cassette |
| 1667, PSB28_for | 5'-TGCGCAGAGGCATACAATAG-3' | *PSB28* |
| 1668, PSB28_rev | 5'-TTACACCCCGCTAACTGACC-3' | |
| 1675, TEF5_for | 5'-AGGGGAAGAGGAAATGAGGA-3' | *TEF5* |
| 1676, TEF5_rev | 5'-TTTGCGTCATGTCCATGTTT-3' | |
| PSB28-1 | 5'-ttgaagaCAAAATGCAGTGCCTTTCCTCC-3' | *PSB28* |
| PSB28-2 | 5'-ttgaagaCAAcAGACCGGTGATATCGCCCATCTCGC-3' | |
| PSB28-3 | 5'-ttgaagaCGGTCTgTTCATGGTGGACGATGAGG-3' | *PSB28* |
| PSB28-4 | 5'-ttgaagacTCCgaaccCTTCTCGAAGCCCAGGTCGTTC-3' | |
| PSB28-Bam | 5'-ggctggatccGCTGCGTCTCTGCAGTTCA-3' | *PSB28* |
| PSB28-Hind | 5'-gcccaagctTGATAGCGTCCAAGGCCTGC-3' | |
| TEF5-qRTPCR_for | 5'-CCACGGTGTACTTTGAGGGC-3' | *TEF5* |
| TEF5-qRTPCR_rev | 5'-CAGCACCACCCAGAATGCAA-3' | |
| CBLP2-qRTPCR_for | 5'-GCCACACCGAGTGGTGTCGTGCG-3' | *CBLP2* |
| CBLP2-qRTPCR_rev | 5'-CCTTGCCGCCCGAGGCGCACAGCG-3' | |
| 1932, F170A-for | 5'-CACGgaagacaaGgcCTGCCGCCCCCTGGAGATCTA-3' | *TEF5* |
| 1933, F170A-rev | 5'-CAGGgaagacttGgcCTGGGAGGGCGTGAGGGTGCG-3' | |
| 1934, F216K/Y223A-for | 5'-GAACgaagacaacaagACGGTGCAGCCCACGGTGgcCTTTGAGGG CATGGACC-3' | *TEF5* |
| 1935, F216K-for | 5'-GAACgaagacaacaagACGGTGCAGCCCACGGTGTAC-3' | |
| 1936, F216K-rev | 5'-GGGCgaagacttcttgACGTTGTTGTTCTCGGCCGA-3' | |
| 1937, Y223A-for | 5'-CAGCgaagacaagcCTTTGAGGGCATGGACCCCAC-3' | *TEF5* |
| 1938, Y223A-rev | 5'-CCATgaagacttAGgcCACCGTGGGCTGCACCGTGAA-3' | |
| 1939, W282A-for | 5'-GCTCgaagacaaCgcGGTGGTGCTGGGCCTGGGCGTG-3' | *TEF5* |
| 1940, W282A-rev | 5'-GCCTgaagacttCgcGAATGCAATGAGCGCCGTCA-3' | |
| 1941, W282D-for | 5'-GCTCgaagacaaCgacGTGGTGCTGGGCCTGGGCGTG-3' | *TEF5* |
| 1942, W282D-rev | 5'-GCCTgaagacttgtcGAATGCAATGAGCGCCGTCA-3' | |

BpiI, BamHI, and HindIII recognition sites are underlined.

**Supplementary Table S3.** MoClo constructs employed and generated.

| Plasmid | Description | Level | Source |
|---|---|---|---|
| pMBS685 | *Chlamydomonas PSB28* genomic sequence | 0 | This study |
| pMBS695 | *Synechocystis psb28-1* CDS with *RBCS2* intron 1 and codon usage optimized for *Chlamydomonas* | 0 | This study |
| pMBS701 | *TEF5* CDS with *RBCS2* introns 1 and 2 | 0 | This study |
| pCM0-020 | *HSP70A-RBCS2* promoter + 5'UTR | 0 | Crozet et al., 2018 |
| pCM0-100 | 3xHA | 0 | Crozet et al., 2018 |
| pCM0-101 | MultiStop | 0 | Crozet et al., 2018 |
| pCM0-119 | *RPL23* 3' UTR | 0 | Crozet et al., 2018 |
| pMBS640 | CDJ1 chloroplast transit peptide | 0 | Niemeyer et al., 2021 |
| pMBS686 | *ARpro*::*PSB28*::*3xHA*::*RPL23-T* | 1 | This study |
| pMBS696 | *ARpro*::*cpTPCDJ1*::*psb28-1*::*3xHA*::*RPL23-T* | 1 | This study |
| pMBS702 | *ARpro*::*TEF5*::*MultiStop::RPL23-T* | 1 | This study |
| pMBS755 | *ARpro*::*TEF5*::*3xHA::RPL23-T* | 1 | This study |

| | | | |
|---|---|---|---|
| pCM1-01 | *PSADpro::aadA::PSAD-T* | 1 | Crozet et al., 2018 |
| pMBS687 | *PSADpro::aadA::PSAD-T::::*<br>*ARpro::PSB28::3xHA::RPL23-T* | 2 | This study |
| pMBS697 | *PSADpro::aadA::PSAD-T::::*<br>*ARpro::cpTPCDJ1::psb28-1::3xHA::RPL23-T* | 2 | This study |
| pMBS703 | *PSADpro::aadA::PSAD-T::::*<br>*ARpro::TEF5::MultiStop::RPL23-T* | 2 | This study |
| pMBS756 | *PSADpro::aadA::PSAD-T::::*<br>*ARpro::TEF5::3xHA::RPL23-T* | 2 | This study |
| pMBS1356 | *PSADpro::aadA::PSAD-T::::*<br>*ARpro::TEF5(W282A)::MultiStop::RPL23-T* | 2 | This study |
| pMBS1357 | *PSADpro::aadA::PSAD-T::::*<br>*ARpro::TEF5(Y223A)::MultiStop::RPL23-T* | 2 | This study |
| pMBS1358 | *PSADpro::aadA::PSAD-T::::*<br>*ARpro::TEF5(F216K)::MultiStop::RPL23-T* | 2 | This study |
| pMBS1359 | *PSADpro::aadA::PSAD-T::::*<br>*ARpro::TEF5(F170A)::MultiStop::RPL23-T* | 2 | This study |
| pMBS1360 | *PSADpro::aadA::PSAD-T::::*<br>*ARpro::TEF5(W282D)::MultiStop::RPL23-T* | 2 | This study |
| pMBS1361 | *PSADpro::aadA::PSAD-T::::*<br>*ARpro::TEF5(F170A/F216K)::MultiStop::RPL23-T* | 2 | This study |
| pMBS1363 | *PSADpro::aadA::PSAD-T::::*<br>*ARpro::TEF5(Y223A/W282A)::MultiStop::RPL23-T* | 2 | This study |
| pMBS1364 | *PSADpro::aadA::PSAD-T::::*<br>*ARpro::TEF5(F170A/F216K/Y223A)::MultiStop::RPL23-T* | 2 | This study |
| pMBS1371 | *PSADpro::aadA::PSAD-T::::*<br>*ARpro::TEF5(F170A/F216K/Y223A/W282A)::MultiStop::RPL23-T* | 2 | This study |
